# Elucidating the maternal and fetal metabolic and immune landscapes of gestational diabetes mellitus with a pan-organ transcriptomic atlas

**DOI:** 10.1016/j.gendis.2025.101551

**Published:** 2025-01-30

**Authors:** Duan Ni, Ralph Nanan

**Affiliations:** a Sydney Medical School Nepean, The University of Sydney, Sydney, New South Wales 2751, Australia; b Charles Perkins Centre, The University of Sydney, Sydney, New South Wales 2050, Australia; c Nepean Hospital, Nepean Blue Mountains Local Health District, Sydney, New South Wales 2751, Australia

Gestational diabetes mellitus (GDM) is the most common pregnancy-associated complication, not only increasing the risk of other pregnancy-related pathologies, but also predisposing both mother and offspring to metabolic disorders like diabetes, obesity, and cardiovascular diseases.^1^ GDM is characterized by abnormal gestational hyperglycemia and insulin-regulated metabolism. Previous studies have also found some GDM-induced changes in maternal organs, including reprogrammed metabolisms in the placenta^2^ and chronic inflammation in adipose tissue.^3^ The effect of GDM on the offspring is less well-described, apart from empirical observations on long-term metabolic conditions.^1^ There is also some previous genomic knowledge regarding GDM-induced changes, but they are generally limited to individual genes as potential biomarkers, lacking systematic insights at a pathway level. Moreover, most aforementioned findings are from stand-alone studies, a more comprehensive overview is warranted, covering different compartments and reconciling the maternal and fetal aspects.

Here, we surveyed most currently available studies about GDM on Gene Expression Omnibus (GEO) and constructed a pan-organ GDM transcriptomic atlas spanning maternal compartments including adipose tissues (subcutaneous and omental fat), peripheral blood mononuclear cells, placenta, and fetal tissues including umbilical vein endothelial cells, amniocytes, and cord blood mononuclear cells (CBMCs) (Fig. 1A and Table S1; 6 RNA sequencing (RNA-seq) datasets, 1 microarray dataset, and 2 single-cell RNA-seq datasets). Comparative analyses for healthy and GDM-affected pregnancies were conducted, profiling the metabolic and immune changes, given their implications in GDM.

**Figure 1:**
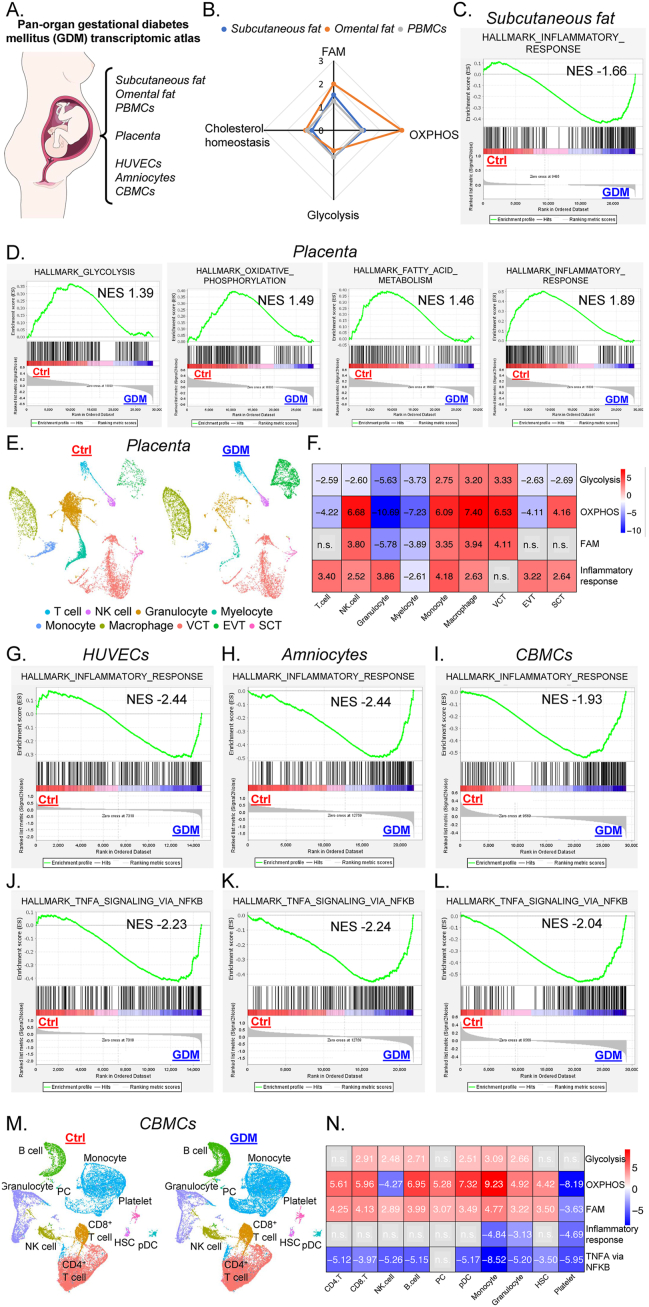
Pan-organ gestational diabetes mellitus (GDM) transcriptomic atlas revealed the metabolic and immune changes associated with GDM across multiple maternal and fetal compartments. **(A)** An overview of the pan-organ GDM transcriptomic atlas in the current study. **(B)** Radar plot for the gene set enrichment analysis (GSEA) enrichment scores for fatty acid metabolism (FAM), oxidative phosphorylation (OXPHOS), glycolysis, and cholesterol homeostasis gene sets in control compared with GDM subcutaneous fat (blue), omental fat (orange), and peripheral blood mononuclear cells (PBMCs, grey). **(C)** GSEA showed the enrichment of the inflammatory response gene set in GDM subcutaneous fat versus control. **(D)** GSEA showed the enrichment of glycolysis, OXPHOS, FAM, and inflammatory response gene set in control versus GDM-affected placenta. **(E)** The uniform manifold approximation and projection (UMAP) plots visualizing the control (left) and GDM (right) placental cellular compositions analyzed by single-cell RNA sequencing. **(F)** The heatmap visualizing the GSEA enrichment scores for glycolysis, OXPHOS, FAM, and inflammatory response gene sets in different populations from control versus GDM placenta. Enrichment scores were specified, with positive values denoted enrichment in control while negative values denoted enrichment in GDM sample; n.s. denoted no significance. **(G**–**I)** GSEA showed the enrichment of inflammatory response gene set in GDM human umbilical vein endothelial cells (HUVECs) (G), amniocytes (H), and cord blood mononuclear cells (CBMCs) (I). **(J**–**L)** GSEA showed the enrichment of the TNFA_signaling_via_NFKB gene set in GDM HUVECs (J), amniocytes (K), and CBMCs (L). **(M)** The UMAP plots visualizing the control (left) and GDM (right) CBMC compositions analyzed by single-cell RNA sequencing. **(N)** The heatmap visualizing the GSEA enrichment scores for glycolysis, OXPHOS, FAM, inflammatory response, and TNFA_signaling_via_NFKB gene sets in different populations from control versus GDM CBMCs. Enrichment scores were specified, with positive values denoted enrichment in control while negative values denoted enrichment in GDM samples; n.s. denoted no significance.

As shown in Figure 1B, gene set enrichment analysis (GSEA) found that relative to GDM, adipose tissues, and peripheral blood mononuclear cells from healthy mothers consistently exhibited enhanced oxidative phosphorylation and fatty acid metabolism signals, similar to a previous report^4^; in contrast, other metabolism-related gene sets like glycolysis and cholesterol homeostasis were less affected (Tables S2–8). One of the hallmarks of diabetes is chronic inflammation. Similarly, the inflammatory response gene set was enriched in GDM subcutaneous fat (Fig. 1C and Table S9), but not in omental fat. Together, these data revealed that GDM systematically shifted the maternal metabolic landscapes, dampening oxidative phosphorylation and fatty acid metabolism, but triggered inflammation solely in subcutaneous fat.

At the maternal-fetal interface, the placenta is likely to play a critical role in GDM manifestation. GSEA revealed that GDM placenta down-regulated most metabolic pathways, together with inflammatory signals (Fig. 1D and Tables S10–13). Furthermore, a single-cell RNA-seq dataset (Table S1) was leveraged to inspect at a single-cell level how GDM affected different cell subsets within the placenta. Single-cell RNA-seq data was clustered and grouped into 9 main cell types including macrophages, monocytes, myelocytes, granulocytes, T cells, natural killer cells, villous cytotrophoblast cells, extravillous trophoblast cells, and syncytiotrophoblast cells (Fig. 1E and Table S14). Similar to the original report, B cells were found in very low abundance. They were thus not included in further analysis, together with cells that could not be annotated to known cell types based on cell-type-specific marker genes (others) (Fig. S1). All populations were present in both healthy and GDM placenta. There was a higher proportion of extravillous trophoblast cells but lower granulocytes and myelocytes in GDM (Fig. 1E). GSEA was run for different populations (Fig. 1F). GDM generally resulted in up-regulation of glycolysis, except in monocytes, macrophages, and villous cytotrophoblast cells. It also promoted oxidative phosphorylation in T cells, granulocytes, myelocytes, and extravillous trophoblast cells, but suppressed oxidative phosphorylation in others. Fatty acid metabolism seemed to be less vulnerable to GDM and was increased in granulocytes and myelocytes but decreased in natural killer cells, monocytes, macrophages, and villous cytotrophoblast cells. Intriguingly, inflammatory signals were enriched in healthy controls when compared with GDM samples for most populations, except for myelocytes and villous cytotrophoblast cells. This was confirmed by gene set score calculation for the inflammatory response gene set as well (Fig. S2A). Collectively, our analyses revealed that GDM conferred multifaceted impacts on placental cells. It altered their metabolic and immune profiles in patterns distinct from the aforementioned maternal compartments.

We next probed into the impacts of GDM on the fetus, with a focus on metabolism and immunity. In contrast to the above maternal analyses, a minimal difference was found for metabolic pathways comparing healthy and GDM samples by GSEA in human umbilical vein endothelial cells, amniocytes, and CBMCs. Instead, enrichments in inflammation-related gene sets in GDM were consistently detected (Fig. 1G–I and Tables S15–20). This seems to be driven by tumor necrosis factor (TNF)-mediated responses, as the “TNFA_signaling_via_NFKB” signal was elevated (Fig. 1J–L and Tables S15–20).

A CBMC single-cell RNA-seq dataset was next analyzed for more in-depth insights (Table S1). As shown in Figure 1M, 10 cell subsets were identified, including CD4^+^ T cells, CD8^+^ T cells, B cells, plasma cells, natural killer cells, monocytes, granulocytes, plasmacytoid dendritic cells, hematopoietic stem cells, and platelets (Table S14). Similar to a prior report, frequencies of CBMC subsets were mildly affected. Single-cell RNA-seq analysis cast more detailed insights into the metabolic impacts conferred by GDM (Fig. 1N). GDM dampened glycolysis in CD8^+^ T cells, natural killer cells, B cells, plasmacytoid dendritic cells, monocytes, and granulocytes. For oxidative phosphorylation and fatty acid metabolism, they were generally higher in healthy controls in all subsets other than natural killer cells and platelets. Single-cell RNA-seq analysis supplied unprecedented insights into the aforementioned elevated inflammatory signals during GDM. It unraveled a profound effect on the innate immune system. GDM significantly promoted the inflammatory response gene set in monocytes and granulocytes, the main immune constituents in CBMCs. Notably, platelets from GDM-affected CBMCs exhibited enhanced inflammatory signals as well. More strikingly, all CBMC populations except plasma cells displayed elevated TNF-mediated signaling, consistent with the RNA-seq results, also validated by gene set score comparisons (Fig. S2B). Together, these results highlighted that GDM conferred less pronounced effects on the infant metabolism, but surprisingly promoted inflammatory signaling.

Here, our pan-organ GDM transcriptomic atlas analyses cast more insights into the influence of GDM on both the mother and the offspring. GDM differentially modified the metabolism networks in the mother but to a less extent in the offspring. At the maternal–fetal interface, the placental metabolisms were rewired in a cell type-specific way, but its inflammatory signal seemed to be blunted. Notably, inflammatory pathways were activated in GDM-affected offspring. Our study provides to the best of our knowledge the most thorough overview of how GDM shifts the immunometabolic landscapes in both mothers and offsprings, supplying in-depth mechanistic insights into the GDM pathophysiology.

Historically, probing GDM via approaches employing metabolomics generally retrieved inconclusive results. Here, our systematic transcriptomic analyses seemed to reveal some consistent immunometabolic alterations associated with GDM, warranting further confirmational studies.

We observed increased inflammation in maternal subcutaneous fat,^3^ although this did not extend to omental fat, possibly hinting at their differential physiological roles. Contrarily, inflammatory signals were consistently enhanced in GDM-impacted infants, possibly increasing their propensity to chronic inflammation and consequently predispositions to inflammatory diseases and metabolic disorders.^1^ It would be of interest to interrogate whether these changes are due to epigenetic modifications and how long they persist over the lifespan.

Our findings highlight the intermediate role of the placenta, separating the maternal and fetal distinct metabolic and immune landscapes. This potentially results in differential GDM-induced pathological sequels, requiring further investigations. Considering the heterogeneity of the placenta, future spatial-scale analysis is warranted for more thorough characterizations.

Finally, current offspring analyses substantially lack insights into other metabolic and immune compartments, like the gastroenteric system. Materials like exfoliated intestinal epithelia cells might provide novel opportunities for mechanistic interrogation.^5^

Collectively, we presented a pan-organ transcriptomic atlas interrogating the maternal and fetal metabolic and immune landscapes during GDM, supplying systematic insights for future related basic and clinical research.

## Funding

This project is supported by the Norman Ernest Bequest Fund.

## CRediT authorship contribution statement

**Duan Ni:** Writing – review & editing, Writing – original draft, Visualization, Validation, Methodology, Investigation, Formal analysis, Data curation, Conceptualization. **Ralph Nanan:** Writing – review & editing, Writing – original draft, Supervision, Resources, Investigation, Funding acquisition, Conceptualization.

## Conflict of interests

The authors have no competing interests to declare.
