# Supplementary material for: Elucidating the maternal and fetal metabolic and immune landscapes of gestational diabetes mellitus with a pan-organ transcriptomic atlas
Source: Genes Dis. 2025 Jan 30;13(1):101551. doi: 10.1016/j.gendis.2025.101551 (PMC12495274; doi:10.1016/j.gendis.2025.101551)
Supplement: Multimedia component 1 [file mmc1.pdf]

Supplementary information

Methods

Data curation and processing

We surveyed most currently available transcriptomic data for GDM in Gene Expression Omnibus. RNA-seq and single cell RNA-seq (scRNA-seq) data were curated from the previous publications as described in Table S1. All samples were used for analysis. RNA-seq data was normalized with DESeq2 (v1.34.0) [1]. The resulting data was used for further analysis. scRNA-seq data was analyzed with *Seurat* (v4) [2]. In brief, scRNA-seq data were obtained from the original publications, underwent quality check and read in *Seurat*. Then, control and GDM samples were integrated with *LIGER* [3] to minimize potential batch effects. In total, 21242 placental cells and 22717 CBMCs were used for downstream analyses.

Data analysis and statistics

Gene set enrichment analysis (GSEA) was carried out with GSEA (v4.3.2) software [4] and *fgsea* package following their tutorials. scRNA-seq data analysis was carried out with the *Seurat* (v4) package [2]. Data were clustered and annotated based on the marker genes adapted from their original publications [5, 6] (Table S14). Gene set score calculation was performed using the *CellCycleScoring* function in *Seurat* based on gene sets from GSEA (v4.3.2).

Codes used for analyses in present work is available: https://github.com/Nidane/GDM-scRNA-seq-

Supplementary Tables and Figure

Table S1. Overview of the transcriptomic data used in current study (N/A: not available).

Compartment	Analytical method	GDM management	Sample size	Sample collection time	GEO ID	Reference
**Subcutaneous fat**	RNA-seq	GDM: 1 without treatment, 3 insulin therapy, 1 metformin	Control: 5; GDM: 5	During caesarean	GSE188799	[7]
**Omental fat**	RNA-seq	GDM: 1 without treatment, 3 insulin therapy, 1 metformin	Control: 5; GDM: 5	During caesarean	GSE188799	[7]
**Peripheral blood mononuclear cell**	RNA-seq	N/A	Control: 8; GDM: 8	Pregnancy week 24-32	GSE92772	[8]
**Placenta**	RNA-seq	N/A	Control: 21; GDM: 18	After delivery	GSE203346	[9]
**Placenta**	scRNA-seq	GDM: 2 without insulin treatment	Control: 2; GDM: 2	During caesarean	GSE173193	[5]
**Umbilical vein endothelial cell**	Microarray	N/A	Control: 3; GDM: 3	After delivery	GSE49524	[10]
**Amniocyte**	RNA-seq	N/A	Control: 8; GDM: 6	Gestational age 16-18 weeks	GSE150621	[11]
**Cord blood mononuclear cell**	RNA-seq	N/A	Control: 20; GDM: 21	After delivery	GSE203346	[9]
**Cord blood mononuclear cell**	scRNA-seq	GDM: 4 without hypoglycemic agents	Control: 3; GDM: 4	After caesarean delivery	GSE212309	[6]

Table S2. Gene set enrichment analysis (GSEA) scores for fatty acid metabolism, oxidative phosphorylation, glycolysis, cholesterol homeostasis, and inflammatory response gene sets comparing control and gestational diabetes mellitus (GDM) subcutaneous fat, omental fat and peripheral blood mononuclear cells (PBMCs). Positive values denote gene set enrichment in control samples while negative values denote gene set enrichment in GDM samples. n.s.: not significant.

	**Fatty acid metabolism**	**Oxidative phosphorylation**	**Glycolysis**	**Cholesterol homeostasis**	**Inflammatory response**
**Subcutaneous fat**	1.58	1.32	n.s.	n.s.	-1.66
**Omental fat**	2.00	2.95	n.s.	1.21	n.s.
**PBMCs**	1.27	1.23	n.s.	n.s.	n.s.

Table S3. Gene set enrichment analysis details for HALLMARK_OXIDATIVE_PHOSPHORYLATION gene set comparing healthy Ctrl and GDM maternal omental fat.

Gene symbol	Rank in gene list	Rank metric score	Running enrichment score	Enrichment
SLC25A6	65	0.329	0.0134	↑ Ctrl
ATP5ME	167	0.264	0.0221	↑ Ctrl
NDUFA1	233	0.247	0.0315	↑ Ctrl
COX7A2	257	0.241	0.0424	↑ Ctrl
ATP5F1E	263	0.24	0.054	↑ Ctrl
COX17	272	0.237	0.0653	↑ Ctrl
COX7C	327	0.226	0.0742	↑ Ctrl
UQCRQ	330	0.226	0.0852	↑ Ctrl
NQO2	337	0.224	0.096	↑ Ctrl
NDUFA2	375	0.218	0.1051	↑ Ctrl
COX7B	402	0.215	0.1146	↑ Ctrl
NDUFB1	451	0.209	0.1229	↑ Ctrl
NDUFA4	456	0.209	0.133	↑ Ctrl
NDUFS4	514	0.202	0.1405	↑ Ctrl
UQCRB	542	0.199	0.1491	↑ Ctrl
NDUFB2	552	0.198	0.1585	↑ Ctrl
COX6C	553	0.198	0.1683	↑ Ctrl
COX5B	576	0.196	0.177	↑ Ctrl
NDUFB6	619	0.191	0.1846	↑ Ctrl
NDUFS6	621	0.191	0.194	↑ Ctrl
ATP5MG	646	0.189	0.2022	↑ Ctrl
NDUFB7	650	0.188	0.2114	↑ Ctrl
ATP5MC1	657	0.188	0.2204	↑ Ctrl
COX8A	692	0.185	0.2281	↑ Ctrl
ATP5MF	697	0.185	0.2371	↑ Ctrl
NDUFC1	703	0.185	0.246	↑ Ctrl
UQCRH	770	0.18	0.252	↑ Ctrl
NDUFA7	779	0.178	0.2604	↑ Ctrl

NDUFA6	781	0.178	0.2692	↑ Ctrl
POLR2F	791	0.178	0.2775	↑ Ctrl
NDUFA3	794	0.177	0.2862	↑ Ctrl
TIMM10	836	0.175	0.293	↑ Ctrl
NDUFB8	843	0.174	0.3013	↑ Ctrl
UQCR11	865	0.173	0.3089	↑ Ctrl
MPC1	885	0.172	0.3166	↑ Ctrl
NDUFAB1	945	0.168	0.3224	↑ Ctrl
COX4I1	1024	0.163	0.327	↑ Ctrl
MRPL11	1037	0.162	0.3345	↑ Ctrl
COX6A1	1040	0.162	0.3424	↑ Ctrl
COX6B1	1124	0.158	0.3466	↑ Ctrl
ETFB	1133	0.157	0.354	↑ Ctrl
NDUFS8	1173	0.155	0.36	↑ Ctrl
MGST3	1206	0.152	0.3661	↑ Ctrl
ECI1	1228	0.151	0.3726	↑ Ctrl
ATP5PF	1234	0.151	0.3798	↑ Ctrl
UQCR10	1264	0.149	0.386	↑ Ctrl
NDUFS3	1308	0.147	0.3914	↑ Ctrl
MRPL34	1318	0.147	0.3982	↑ Ctrl
ACAA2	1326	0.146	0.4051	↑ Ctrl
MRPS12	1343	0.146	0.4116	↑ Ctrl
MRPS15	1353	0.145	0.4184	↑ Ctrl
ATP6V1F	1419	0.142	0.4226	↑ Ctrl
ATP5PD	1423	0.142	0.4295	↑ Ctrl
ATP5PO	1431	0.142	0.4362	↑ Ctrl
NDUFB3	1503	0.139	0.44	↑ Ctrl
HSD17B10	1510	0.139	0.4466	↑ Ctrl
TIMM13	1531	0.138	0.4526	↑ Ctrl
ATP6V0B	1620	0.134	0.4554	↑ Ctrl
DECR1	1636	0.134	0.4614	↑ Ctrl
NDUFB5	1696	0.132	0.4653	↑ Ctrl
TIMM17A	1724	0.131	0.4706	↑ Ctrl
NDUFB4	1802	0.128	0.4736	↑ Ctrl
NDUFS7	1826	0.127	0.4789	↑ Ctrl
TIMM9	1838	0.127	0.4846	↑ Ctrl
NDUFV2	1842	0.127	0.4908	↑ Ctrl
MRPS11	1889	0.125	0.4949	↑ Ctrl
CYB5A	1899	0.125	0.5007	↑ Ctrl
MRPS22	1943	0.123	0.5049	↑ Ctrl
SURF1	1944	0.123	0.511	↑ Ctrl

NDUFC2	1960	0.122	0.5164	↑ Ctrl
ATP5MC2	1993	0.121	0.521	↑ Ctrl
NDUFA8	1994	0.121	0.5269	↑ Ctrl
SUCLG1	2024	0.12	0.5316	↑ Ctrl
MRPL15	2033	0.119	0.5371	↑ Ctrl
ATP6V1D	2077	0.118	0.5411	↑ Ctrl
ATP6V1E1	2087	0.118	0.5465	↑ Ctrl
FDX1	2090	0.118	0.5522	↑ Ctrl
ATP5MC3	2151	0.116	0.5554	↑ Ctrl
SDHB	2155	0.116	0.5609	↑ Ctrl
ATP5F1C	2163	0.116	0.5663	↑ Ctrl
VDAC3	2171	0.115	0.5717	↑ Ctrl
COX7A2L	2188	0.115	0.5767	↑ Ctrl
NDUFA9	2282	0.112	0.5782	↑ Ctrl
SLC25A5	2403	0.11	0.5785	↑ Ctrl
ATP6V0E1	2432	0.109	0.5826	↑ Ctrl
NDUFA5	2436	0.109	0.5879	↑ Ctrl
ETFA	2439	0.109	0.5932	↑ Ctrl
IDH3B	2479	0.108	0.5968	↑ Ctrl
TOMM22	2514	0.107	0.6006	↑ Ctrl
ISCU	2517	0.107	0.6058	↑ Ctrl
ECH1	2567	0.106	0.6089	↑ Ctrl
CYC1	2677	0.103	0.6093	↑ Ctrl
SUPV3L1	2740	0.101	0.6116	↑ Ctrl
ATP6V1G1	2815	0.099	0.6133	↑ Ctrl
PDHB	2884	0.098	0.6152	↑ Ctrl
BDH2	3061	0.094	0.6122	↑ Ctrl
UQCRFS1	3098	0.093	0.6152	↑ Ctrl
ATP5F1D	3131	0.092	0.6184	↑ Ctrl
ATP5PB	3138	0.092	0.6227	↑ Ctrl
FH	3167	0.091	0.626	↑ Ctrl
HTRA2	3182	0.091	0.6299	↑ Ctrl
UQCRC1	3468	0.085	0.6218	↑ Ctrl
PHB2	3473	0.085	0.6258	↑ Ctrl
VDAC2	3492	0.085	0.6292	↑ Ctrl
SLC25A11	3499	0.085	0.6331	↑ Ctrl
IDH2	3604	0.082	0.6327	↑ Ctrl
GPX4	3613	0.082	0.6364	↑ Ctrl
CYCS	3631	0.082	0.6397	↑ Ctrl
MTX2	3644	0.082	0.6433	↑ Ctrl
PRDX3	3660	0.082	0.6466	↑ Ctrl

COX5A	3788	0.079	0.6451	↑ Ctrl
HCCS	3828	0.079	0.6472	↑ Ctrl
MRPS30	3830	0.078	0.6511	↑ Ctrl
GRPEL1	3854	0.078	0.6539	↑ Ctrl
TIMM50	3888	0.077	0.6563	↑ Ctrl
PDK4	3920	0.077	0.6588	↑ Ctrl
MDH1	3924	0.077	0.6625	↑ Ctrl
ACAT1	3943	0.077	0.6655	↑ Ctrl
SDHD	3955	0.076	0.6687	↑ Ctrl
LDHB	4211	0.072	0.6613	↑ Ctrl
UQCRC2	4220	0.071	0.6644	↑ Ctrl
RHOT1	4234	0.071	0.6674	↑ Ctrl
SDHC	4238	0.071	0.6708	↑ Ctrl
ATP5F1B	4273	0.07	0.6728	↑ Ctrl
MRPL35	4336	0.07	0.6735	↑ Ctrl
BAX	4500	0.067	0.6698	-
IDH3G	4501	0.067	0.6731	-
NDUFV1	4692	0.063	0.668	-
ECHS1	4739	0.063	0.6692	-
ACAA1	4871	0.061	0.6665	-
ATP6V1H	4940	0.06	0.6666	-
LDHA	5030	0.059	0.6656	-
ATP6V1C1	5100	0.058	0.6655	-
IDH3A	5172	0.057	0.6652	-
PHYH	5207	0.056	0.6665	-
SLC25A4	5253	0.055	0.6673	-
NDUFS2	5277	0.055	0.669	-
SLC25A3	5310	0.055	0.6703	-
ATP5F1A	5492	0.052	0.6651	-
LRPPRC	5552	0.05	0.665	-
COX11	5770	0.048	0.658	-
SUCLA2	5941	0.045	0.6529	-
OAT	5951	0.045	0.6548	-
DLD	6165	0.044	0.6478	-
AFG3L2	6369	0.041	0.641	-
SLC25A20	6440	0.04	0.64	-
HADHA	6454	0.04	0.6414	-
IDH1	6462	0.04	0.643	-
AIFM1	6541	0.038	0.6415	-
MAOB	6574	0.038	0.642	-
ACADM	6602	0.038	0.6427	-

OXA1L	6754	0.036	0.638	-
PDHA1	6913	0.034	0.6328	-
HADHB	7171	0.031	0.6233	-
SLC25A12	7308	0.03	0.6189	-
HSPA9	7478	0.027	0.613	-
ISCA1	7543	0.026	0.6115	-
VDAC1	7552	0.026	0.6124	-
MTRF1	7575	0.026	0.6128	-
COX15	7716	0.024	0.6079	-
MDH2	7795	0.023	0.6057	-
ATP1B1	7895	0.022	0.6025	-
CASP7	8054	0.02	0.5967	-
ACADVL	8218	0.018	0.5906	-
BCKDHA	8313	0.017	0.5874	-
GLUD1	8343	0.016	0.5869	-
OPA1	8386	0.016	0.5859	-
ACADSB	8458	0.015	0.5836	-
ETFDH	8737	0.012	0.5722	-
DLAT	9032	0.008	0.5599	-
MTRR	9082	0.007	0.5582	-
PDP1	12707	-0.001	0.4021	-
DLST	12761	-0.001	0.3999	-
NDUFS1	13142	-0.006	0.3838	-
ATP6V0C	13206	-0.007	0.3814	-
SDHA	13246	-0.008	0.3801	-
TIMM8B	13446	-0.01	0.3721	-
TOMM70	13461	-0.011	0.372	-
IMMT	13684	-0.013	0.3631	-
PMPCA	13897	-0.016	0.3547	-
GOT2	14152	-0.019	0.3447	-
GPI	14183	-0.02	0.3444	-
CS	14891	-0.029	0.3154	-
ALAS1	15230	-0.033	0.3024	-
CYB5R3	15281	-0.033	0.3019	-
RETSAT	15912	-0.042	0.2769	-
PDHX	16559	-0.049	0.2514	-
ATP6AP1	16566	-0.049	0.2536	-
ALDH6A1	16683	-0.051	0.2511	-
ABCB7	16821	-0.052	0.2477	-
NNT	17020	-0.056	0.2419	-
TCIRG1	17081	-0.057	0.2422	-

ACO2	17168	-0.058	0.2413	-
RHOT2	17264	-0.059	0.2401	-
OGDH	17490	-0.062	0.2335	-
MFN2	17719	-0.065	0.2269	-
POR	17837	-0.067	0.2252	-
FXN	18133	-0.072	0.216	-
CPT1A	19783	-0.1	0.1499	-
COX10	21025	-0.13	0.1029	-

Table S4. Gene set enrichment analysis details for HALLMARK_FATTY_ACID_METABOLISM gene set comparing healthy Ctrl and GDM maternal omental fat.

Gene symbol	Rank in gene list	Rank metric score	Running enrichment score	Enrichment
ACSM3	250	0.244	0.0122	↑ Ctrl
PTS	313	0.229	0.0311	↑ Ctrl
HAO2	612	0.192	0.0364	↑ Ctrl
HPGD	774	0.179	0.0463	↑ Ctrl
BMPR1B	897	0.171	0.0572	↑ Ctrl
CIDEA	909	0.17	0.0727	↑ Ctrl
ECI1	1228	0.151	0.0733	↑ Ctrl
ACAA2	1326	0.146	0.0829	↑ Ctrl
OSTC	1340	0.146	0.0961	↑ Ctrl
IDI1	1345	0.146	0.1096	↑ Ctrl
CRYZ	1364	0.145	0.1225	↑ Ctrl
MIF	1444	0.141	0.1325	↑ Ctrl
INMT	1477	0.14	0.1443	↑ Ctrl
GSTZ1	1489	0.14	0.1569	↑ Ctrl
HSD17B10	1510	0.139	0.1692	↑ Ctrl
CBR3	1567	0.137	0.1796	↑ Ctrl
H2AZ1	1576	0.136	0.1921	↑ Ctrl
DECR1	1636	0.134	0.2022	↑ Ctrl
ERP29	1682	0.132	0.2127	↑ Ctrl
PRDX6	1703	0.131	0.2243	↑ Ctrl
LGALS1	1804	0.128	0.232	↑ Ctrl
PCBD1	1885	0.125	0.2404	↑ Ctrl
SUCLG1	2024	0.12	0.2457	↑ Ctrl
UROS	2250	0.113	0.2467	↑ Ctrl
AUH	2348	0.111	0.2529	↑ Ctrl
LTC4S	2447	0.108	0.2589	↑ Ctrl
IDH3B	2479	0.108	0.2677	↑ Ctrl
ECH1	2567	0.106	0.2739	↑ Ctrl
BLVRA	2571	0.106	0.2838	↑ Ctrl

NTHL1	2583	0.105	0.2932	↑ Ctrl
FMO1	2694	0.102	0.2981	↑ Ctrl
HMGCL	2713	0.102	0.3069	↑ Ctrl
PDHB	2884	0.098	0.3088	↑ Ctrl
MIX23	3064	0.094	0.3099	↑ Ctrl
FH	3167	0.091	0.3141	↑ Ctrl
GLUL	3251	0.089	0.319	↑ Ctrl
CA6	3322	0.088	0.3243	↑ Ctrl
ODC1	3423	0.086	0.3281	↑ Ctrl
PSME1	3476	0.085	0.3338	↑ Ctrl
UROD	3576	0.083	0.3374	↑ Ctrl
UGDH	3640	0.082	0.3424	↑ Ctrl
GPD2	3657	0.082	0.3494	↑ Ctrl
ADSL	3687	0.081	0.3557	↑ Ctrl
GRHPR	3697	0.081	0.363	↑ Ctrl
CBR1	3747	0.08	0.3684	↑ Ctrl
HCCS	3828	0.079	0.3723	↑ Ctrl
MDH1	3924	0.077	0.3755	↑ Ctrl
SDHD	3955	0.076	0.3814	↑ Ctrl
SMS	4062	0.074	0.3838	↑ Ctrl
S100A10	4075	0.074	0.3903	↑ Ctrl
RDH11	4223	0.071	0.3907	↑ Ctrl
SDHC	4238	0.071	0.3968	↑ Ctrl
NSDHL	4243	0.071	0.4033	↑ Ctrl
HSD17B11	4260	0.071	0.4093	↑ Ctrl
VNN1	4303	0.07	0.4141	↑ Ctrl
HSPH1	4319	0.07	0.42	↑ Ctrl
ACAT2	4361	0.069	0.4247	↑ Ctrl
NBN	4436	0.068	0.4279	↑ Ctrl
MAOA	4493	0.067	0.4318	↑ Ctrl
IDH3G	4501	0.067	0.4378	↑ Ctrl
GCDH	4511	0.066	0.4437	↑ Ctrl
HMGCS2	4598	0.065	0.4461	↑ Ctrl
ECI2	4607	0.065	0.4519	↑ Ctrl
ECHS1	4739	0.063	0.4522	↑ Ctrl
ACAA1	4871	0.061	0.4523	↑ Ctrl
HSP90AA1	4948	0.06	0.4547	↑ Ctrl
MCEE	4992	0.059	0.4584	↑ Ctrl
LDHA	5030	0.059	0.4624	↑ Ctrl
RAP1GDS1	5153	0.057	0.4625	↑ Ctrl
GABARAPL1	5445	0.052	0.4549	↑ Ctrl

HSDL2	5681	0.049	0.4494	↑ Ctrl
HSD17B4	5701	0.049	0.4531	↑ Ctrl
ACSL4	5715	0.048	0.4571	↑ Ctrl
BPHL	5840	0.047	0.4562	↑ Ctrl
ACADL	5859	0.047	0.4598	↑ Ctrl
SUCLA2	5941	0.045	0.4606	↑ Ctrl
CYP4A22	6035	0.045	0.4608	↑ Ctrl
METAP1	6043	0.045	0.4647	↑ Ctrl
HMGCS1	6162	0.044	0.4638	↑ Ctrl
DLD	6165	0.044	0.4678	↑ Ctrl
ACSL1	6235	0.043	0.4688	↑ Ctrl
IDH1	6462	0.04	0.4628	-
UBE2L6	6554	0.038	0.4625	-
ACADM	6602	0.038	0.464	-
ADIPOR2	6758	0.036	0.4607	-
PDHA1	6913	0.034	0.4573	-
HADHB	7171	0.031	0.4492	-
HADH	7348	0.029	0.4443	-
APEX1	7370	0.029	0.4461	-
MLYCD	7523	0.027	0.4421	-
ACOT8	7609	0.026	0.4408	-
ACADS	7644	0.025	0.4417	-
ALDOA	7659	0.025	0.4435	-
MDH2	7795	0.023	0.4399	-
ALDH1A1	7876	0.022	0.4385	-
CD36	8179	0.018	0.4273	-
ACADVL	8218	0.018	0.4273	-
CEL	8261	0.017	0.4272	-
YWHAH	8592	0.013	0.4142	-
PTPRG	8615	0.013	0.4145	-
ETFDH	8737	0.012	0.4104	-
CPOX	8880	0.01	0.4052	-
CYP1A1	9139	0.007	0.3948	-
CPT2	9243	0.005	0.3908	-
SERINC1	9542	0.002	0.3782	-
HIBCH	9606	0.001	0.3756	-
HSD17B7	9665	0.001	0.3732	-
G0S2	9688	0	0.3723	-
SLC22A5	9797	0	0.3676	-
GAD2	10988	0	0.3165	-
FABP1	12476	0	0.2525	-

ALDH9A1	12720	-0.001	0.2422	-
AQP7	12755	-0.001	0.2408	-
DLST	12761	-0.001	0.2407	-
BCKDHB	12878	-0.003	0.236	-
ME1	12980	-0.004	0.232	-
SDHA	13246	-0.008	0.2213	-
SUCLG2	13330	-0.009	0.2186	-
NCAPH2	13423	-0.01	0.2156	-
ENO2	13464	-0.011	0.2148	-
ALDH3A2	13504	-0.011	0.2142	-
ALDH3A1	13566	-0.012	0.2127	-
CA4	13571	-0.012	0.2136	-
ACOT2	13619	-0.012	0.2128	-
KMT5A	14039	-0.018	0.1964	-
RDH16	14149	-0.019	0.1935	-
REEP6	14206	-0.02	0.193	-
GAPDHS	14270	-0.021	0.1922	-
DHCR24	14367	-0.022	0.1902	-
PPARA	14630	-0.025	0.1813	-
ENO3	15011	-0.03	0.1678	-
ADH7	15050	-0.031	0.1691	-
EPHX1	15323	-0.034	0.1606	-
CA2	15401	-0.035	0.1605	-
ADH1C	15429	-0.035	0.1627	-
ACSL5	15655	-0.038	0.1566	-
GPD1	15674	-0.039	0.1595	-
RETSAT	15912	-0.042	0.1533	-
TDO2	17033	-0.056	0.1104	-
ACO2	17168	-0.058	0.1101	-
MGLL	17180	-0.058	0.115	-
ELOVL5	17187	-0.058	0.1202	-
CRAT	17697	-0.065	0.1045	-
TP53INP2	17740	-0.065	0.1088	-
ACSS1	17907	-0.069	0.1081	-
AADAT	17988	-0.07	0.1113	-
ACOX1	18237	-0.074	0.1075	-
AOC3	18522	-0.078	0.1027	-
FASN	18649	-0.08	0.1048	-
CD1D	18977	-0.086	0.0988	-
EHHADH	19241	-0.09	0.096	-
CPT1A	19783	-0.1	0.0822	-

CYP4A11	19928	-0.103	0.0857	-
IL4I1	20333	-0.112	0.0789	-
ALAD	21043	-0.13	0.0607	-
D2HGDH	21050	-0.131	0.0727	-
XIST	21416	-0.142	0.0704	-
FABP2	21923	-0.164	0.0641	-

Table S5. Gene set enrichment analysis details for HALLMARK_OXIDATIVE_PHOSPHORYLATION gene set comparing healthy Ctrl and GDM maternal subcutaneous fat.

Gene symbol	Rank in gene list	Rank metric score	Running enrichment score	Enrichment
MTRF1	347	0.113	0.0071	↑ Ctrl
SUCLA2	550	0.097	0.0173	↑ Ctrl
ALDH6A1	728	0.087	0.0267	↑ Ctrl
NQO2	739	0.087	0.0432	↑ Ctrl
PDK4	1076	0.075	0.0434	↑ Ctrl
MAOB	1391	0.067	0.043	↑ Ctrl
ACAT1	1523	0.064	0.0499	↑ Ctrl
SUCLG1	1623	0.063	0.0579	↑ Ctrl
ECI1	1643	0.062	0.0692	↑ Ctrl
CYB5A	1687	0.062	0.0793	↑ Ctrl
RETSAT	1752	0.06	0.0884	↑ Ctrl
PDHX	1778	0.06	0.099	↑ Ctrl
CASP7	1780	0.06	0.1106	↑ Ctrl
OPA1	1788	0.06	0.122	↑ Ctrl
LDHB	1928	0.057	0.1272	↑ Ctrl
UQCRB	1974	0.057	0.1363	↑ Ctrl
BDH2	2002	0.056	0.1461	↑ Ctrl
LRPPRC	2119	0.054	0.1517	↑ Ctrl
NDUFS4	2162	0.054	0.1603	↑ Ctrl
NDUFS1	2266	0.052	0.1661	↑ Ctrl
IDH1	2309	0.052	0.1743	↑ Ctrl
MRPS22	2347	0.051	0.1827	↑ Ctrl
DLD	2653	0.047	0.1788	↑ Ctrl
NNT	2696	0.046	0.186	↑ Ctrl
COX7A2	2708	0.046	0.1946	↑ Ctrl
ATP6V1D	2749	0.046	0.2018	↑ Ctrl
IDH2	2973	0.043	0.2006	↑ Ctrl
ATP5PO	3042	0.042	0.206	↑ Ctrl
RHOT1	3172	0.041	0.2084	↑ Ctrl
MPC1	3187	0.041	0.2158	↑ Ctrl
PRDX3	3236	0.041	0.2217	↑ Ctrl

ACADM	3316	0.04	0.2261	↑ Ctrl
NDUFV2	3490	0.038	0.226	↑ Ctrl
ATP6V1E1	3586	0.037	0.2292	↑ Ctrl
COX11	3712	0.036	0.2308	↑ Ctrl
ACAA2	3726	0.036	0.2373	↑ Ctrl
ATP6V1H	3963	0.034	0.2337	↑ Ctrl
ATP5PD	4038	0.033	0.2371	↑ Ctrl
NDUFA5	4063	0.033	0.2425	↑ Ctrl
NDUFA7	4068	0.033	0.2488	↑ Ctrl
SDHB	4256	0.032	0.247	↑ Ctrl
ACO2	4295	0.031	0.2514	↑ Ctrl
ACAA1	4462	0.03	0.2501	↑ Ctrl
MRPL15	4547	0.03	0.2523	↑ Ctrl
ATP6V1C1	4589	0.029	0.2562	↑ Ctrl
NDUFAB1	4643	0.029	0.2595	↑ Ctrl
HSD17B10	4658	0.029	0.2645	↑ Ctrl
PHYH	4681	0.028	0.2691	↑ Ctrl
ATP5PB	4683	0.028	0.2746	↑ Ctrl
COX7C	4788	0.028	0.2756	↑ Ctrl
ATP5MC1	4805	0.028	0.2803	↑ Ctrl
BCKDHA	4844	0.027	0.2839	↑ Ctrl
UQCR10	4877	0.027	0.2878	↑ Ctrl
MRPL34	4888	0.027	0.2927	↑ Ctrl
NDUFB6	4922	0.027	0.2965	↑ Ctrl
DLST	5030	0.026	0.2969	↑ Ctrl
NDUFB8	5089	0.026	0.2994	↑ Ctrl
ATP5F1E	5178	0.025	0.3005	↑ Ctrl
COX7A2L	5180	0.025	0.3053	↑ Ctrl
FH	5367	0.024	0.302	↑ Ctrl
ATP5PF	5498	0.023	0.3009	↑ Ctrl
NDUFA1	5591	0.023	0.3013	↑ Ctrl
ISCA1	5726	0.022	0.2998	↑ Ctrl
TOMM70	5763	0.021	0.3024	↑ Ctrl
NDUFA4	5821	0.021	0.304	↑ Ctrl
ETFA	5864	0.021	0.3063	↑ Ctrl
PDHB	5905	0.02	0.3085	↑ Ctrl
COX5B	5910	0.02	0.3123	↑ Ctrl
MRPS11	5991	0.02	0.3128	↑ Ctrl
HTRA2	6008	0.02	0.316	↑ Ctrl
ETFDH	6055	0.019	0.3178	↑ Ctrl
RHOT2	6304	0.018	0.3106	↑ Ctrl

NDUFB5	6357	0.017	0.3118	↑ Ctrl
NDUFC2	6477	0.017	0.31	↑ Ctrl
ATP5F1C	6481	0.017	0.3132	↑ Ctrl
POLR2F	6545	0.017	0.3138	↑ Ctrl
MTRR	6552	0.017	0.3168	↑ Ctrl
NDUFA2	6676	0.016	0.3146	↑ Ctrl
MRPS12	6717	0.016	0.316	↑ Ctrl
SLC25A12	6745	0.016	0.3179	↑ Ctrl
COX6A1	6793	0.015	0.3189	↑ Ctrl
NDUFS7	6805	0.015	0.3214	↑ Ctrl
ISCU	6991	0.014	0.3162	↑ Ctrl
TIMM50	7004	0.014	0.3184	↑ Ctrl
GPX4	7047	0.014	0.3193	↑ Ctrl
ATP5ME	7061	0.014	0.3214	↑ Ctrl
ATP5MG	7096	0.013	0.3225	↑ Ctrl
MGST3	7136	0.013	0.3234	↑ Ctrl
IDH3B	7194	0.013	0.3235	↑ Ctrl
COX15	7227	0.013	0.3246	↑ Ctrl
NDUFA9	7348	0.012	0.3218	-
HSPA9	7526	0.011	0.3163	-
NDUFB7	7645	0.01	0.3133	-
HADHB	7795	0.01	0.3087	-
MRPS15	7958	0.009	0.3034	-
TIMM10	7973	0.009	0.3045	-
IMMT	8051	0.008	0.3028	-
FDX1	8092	0.008	0.3026	-
COX4I1	8164	0.008	0.3011	-
ETFB	8224	0.007	0.2999	-
SLC25A4	8325	0.007	0.297	-
ACADVL	8461	0.006	0.2923	-
MRPL35	8487	0.006	0.2924	-
VDAC2	8491	0.006	0.2934	-
IDH3A	8571	0.005	0.2911	-
ABCB7	8606	0.005	0.2906	-
PHB2	8637	0.005	0.2902	-
VDAC3	8715	0.004	0.2878	-
OAT	8716	0.004	0.2886	-
NDUFB3	8788	0.004	0.2863	-
MRPL11	8864	0.004	0.2838	-
HADHA	9038	0.003	0.2769	-
COX10	9040	0.003	0.2774	-

ATP5F1B	9048	0.003	0.2776	-
NDUFC1	9055	0.003	0.2779	-
IDH3G	9066	0.003	0.2779	-
SDHA	9143	0.002	0.2751	-
UQCRC2	9146	0.002	0.2754	-
UQCRFS1	9189	0.002	0.274	-
GLUD1	9212	0.002	0.2734	-
ECHS1	9291	0.001	0.2702	-
UQCR11	10179	0	0.232	-
NDUFS8	12963	0	0.1122	-
CYCS	13114	0	0.1057	-
SURF1	13168	-0.001	0.1035	-
SUPV3L1	13293	-0.001	0.0984	-
VDAC1	13346	-0.001	0.0964	-
NDUFA6	13473	-0.002	0.0914	-
CYB5R3	13508	-0.002	0.0903	-
FXN	13567	-0.002	0.0883	-
NDUFS3	13756	-0.003	0.0809	-
COX17	13863	-0.004	0.0771	-
NDUFB4	13924	-0.004	0.0754	-
SLC25A3	13937	-0.005	0.0757	-
OGDH	13964	-0.005	0.0755	-
PDHA1	14054	-0.005	0.0727	-
NDUFV1	14128	-0.005	0.0706	-
ATP5F1A	14203	-0.006	0.0686	-
UQCRC1	14359	-0.007	0.0633	-
PMPCA	14380	-0.007	0.0638	-
ACADSB	14455	-0.007	0.0621	-
MDH2	14477	-0.008	0.0626	-
GPI	14528	-0.008	0.062	-
NDUFS2	14832	-0.01	0.0508	-
LDHA	14863	-0.01	0.0515	-
MFN2	14934	-0.01	0.0505	-
CYC1	14983	-0.011	0.0505	-
DLAT	15040	-0.011	0.0502	-
COX6B1	15067	-0.011	0.0512	-
NDUFS6	15072	-0.011	0.0532	-
OXA1L	15114	-0.011	0.0536	-
POR	15137	-0.011	0.0549	-
AFG3L2	15193	-0.012	0.0549	-
ATP6V0E1	15409	-0.013	0.0481	-

ATP6V1G1	15454	-0.013	0.0487	-
MDH1	15580	-0.014	0.046	-
GRPEL1	15587	-0.014	0.0484	-
SLC25A5	15758	-0.015	0.0439	-
ATP6V1F	15924	-0.016	0.0399	-
PDP1	16003	-0.016	0.0397	-
AIFM1	16042	-0.016	0.0412	-
UQCRQ	16050	-0.016	0.0441	-
ATP5MC2	16270	-0.017	0.0381	-
UQCRH	16339	-0.018	0.0386	-
BAX	16459	-0.018	0.037	-
NDUFB1	16519	-0.018	0.038	-
NDUFA3	16526	-0.018	0.0414	-
COX7B	16569	-0.019	0.0432	-
ATP5MC3	16806	-0.02	0.037	-
ATP5MF	16893	-0.021	0.0373	-
ATP5F1D	17056	-0.022	0.0346	-
ECH1	17168	-0.023	0.0343	-
SDHD	17213	-0.023	0.0368	-
NDUFA8	17287	-0.023	0.0382	-
NDUFB2	17338	-0.024	0.0407	-
SLC25A11	17680	-0.026	0.031	-
SDHC	17850	-0.027	0.029	-
CS	17908	-0.028	0.032	-
ALAS1	17916	-0.028	0.037	-
MRPS30	17953	-0.028	0.0409	-
GOT2	18036	-0.028	0.0429	-
ATP6AP1	18571	-0.032	0.0262	-
TIMM13	18590	-0.033	0.0318	-
COX8A	18874	-0.035	0.0264	-
COX5A	18878	-0.035	0.0331	-
COX6C	18983	-0.036	0.0357	-
TOMM22	19188	-0.037	0.0342	-
ATP6V0C	19231	-0.038	0.0397	-
CPT1A	19364	-0.039	0.0417	-
TIMM9	19611	-0.041	0.0392	-
DECR1	19819	-0.043	0.0387	-
TIMM17A	19829	-0.043	0.0468	-
ATP6V0B	19877	-0.044	0.0533	-
TIMM8B	20518	-0.051	0.0358	-
MTX2	20574	-0.052	0.0435	-

SLC25A20	20607	-0.052	0.0524	-
TCIRG1	20637	-0.053	0.0614	-
HCCS	20671	-0.053	0.0703	-
ATP1B1	22729	-0.104	0.002	-
SLC25A6	23186	-0.141	0.0098	-

Table S6. Gene set enrichment analysis details for HALLMARK_FATTY_ACID_METABOLISM gene set comparing healthy Ctrl and GDM maternal subcutaneous fat.

Gene symbol	Rank in gene list	Rank metric score	Running enrichment score	Enrichment
GLUL	161	0.144	0.0213	↑ Ctrl
ALDH1A1	181	0.14	0.0479	↑ Ctrl
CIDEA	219	0.133	0.0724	↑ Ctrl
MAOA	263	0.125	0.0951	↑ Ctrl
GAPDHS	484	0.102	0.1057	↑ Ctrl
SUCLA2	550	0.097	0.122	↑ Ctrl
ACSL1	597	0.094	0.1385	↑ Ctrl
MCEE	654	0.092	0.1541	↑ Ctrl
ACADL	695	0.089	0.1699	↑ Ctrl
FASN	828	0.083	0.1805	↑ Ctrl
ADH1C	850	0.082	0.1958	↑ Ctrl
BCKDHB	1324	0.069	0.189	↑ Ctrl
DHCR24	1440	0.066	0.197	↑ Ctrl
ACSM3	1473	0.065	0.2084	↑ Ctrl
HMGCS1	1587	0.063	0.216	↑ Ctrl
SUCLG1	1623	0.063	0.2268	↑ Ctrl
FMO1	1642	0.062	0.2382	↑ Ctrl
ECI1	1643	0.062	0.2505	↑ Ctrl
HPGD	1707	0.061	0.2598	↑ Ctrl
RETSAT	1752	0.06	0.2698	↑ Ctrl
ENO3	1757	0.06	0.2815	↑ Ctrl
PRDX6	1796	0.059	0.2915	↑ Ctrl
GCDH	1924	0.057	0.2973	↑ Ctrl
GABARAPL1	2080	0.055	0.3015	↑ Ctrl
CYP4A22	2127	0.054	0.3101	↑ Ctrl
ACOT8	2128	0.054	0.3208	↑ Ctrl
HADH	2183	0.053	0.3289	↑ Ctrl
IDH1	2309	0.052	0.3337	↑ Ctrl
CRYZ	2316	0.051	0.3435	↑ Ctrl
HSDL2	2421	0.05	0.3489	↑ Ctrl
TDO2	2467	0.049	0.3567	↑ Ctrl
XIST	2633	0.047	0.3589	↑ Ctrl

DLD	2653	0.047	0.3673	↑ Ctrl
HIBCH	2658	0.047	0.3764	↑ Ctrl
CYP1A1	3177	0.041	0.3622	↑ Ctrl
AOC3	3242	0.04	0.3674	↑ Ctrl
ACADM	3316	0.04	0.372	↑ Ctrl
IDI1	3531	0.038	0.3702	↑ Ctrl
OSTC	3539	0.038	0.3773	↑ Ctrl
SERINC1	3583	0.037	0.3828	↑ Ctrl
ACAA2	3726	0.036	0.3837	↑ Ctrl
HSD17B4	3752	0.036	0.3897	↑ Ctrl
ACO2	4295	0.031	0.3725	↑ Ctrl
MLYCD	4296	0.031	0.3787	↑ Ctrl
AUH	4386	0.031	0.3809	↑ Ctrl
BPHL	4459	0.03	0.3837	↑ Ctrl
ACAA1	4462	0.03	0.3895	↑ Ctrl
H2AZ1	4583	0.029	0.39	↑ Ctrl
GSTZ1	4642	0.029	0.3932	↑ Ctrl
HSD17B10	4658	0.029	0.3982	↑ Ctrl
ACSS1	4992	0.026	0.389	-
DLST	5030	0.026	0.3926	-
SLC22A5	5279	0.024	0.3867	-
FH	5367	0.024	0.3876	-
HSP90AA1	5474	0.023	0.3877	-
VNN1	5751	0.021	0.38	-
PDHB	5905	0.02	0.3775	-
ODC1	5979	0.02	0.3783	-
UROS	6005	0.02	0.3811	-
ETFDH	6055	0.019	0.3828	-
AQP7	6143	0.019	0.3828	-
GPD1	6220	0.018	0.3831	-
NBN	6380	0.017	0.3797	-
ME1	6518	0.017	0.3771	-
HSD17B11	6565	0.017	0.3784	-
ADH7	6603	0.016	0.38	-
BMPR1B	6972	0.014	0.367	-
ADIPOR2	7068	0.014	0.3656	-
ACAT2	7117	0.013	0.3662	-
IDH3B	7194	0.013	0.3654	-
SUCLG2	7200	0.013	0.3677	-
EPHX1	7298	0.012	0.366	-
CYP4A11	7483	0.011	0.3603	-

HADHB	7795	0.01	0.3488	-
HMGCL	8166	0.008	0.3344	-
ELOVL5	8308	0.007	0.3297	-
ACADVL	8461	0.006	0.3243	-
ACADS	8472	0.006	0.3251	-
BLVRA	8557	0.005	0.3225	-
ERP29	8685	0.005	0.318	-
CBR1	8693	0.005	0.3186	-
PTPRG	8830	0.004	0.3135	-
IDH3G	9066	0.003	0.3039	-
SDHA	9143	0.002	0.301	-
MGLL	9220	0.002	0.2981	-
LGALS1	9283	0.001	0.2957	-
ECHS1	9291	0.001	0.2956	-
CA6	9572	0	0.2836	-
KMT5A	10239	0	0.255	-
CPT2	10505	0	0.2436	-
METAP1	10573	0	0.2407	-
HMGCS2	10780	0	0.2318	-
FABP1	12881	0	0.1415	-
MIF	13136	0	0.1307	-
NSDHL	13172	-0.001	0.1293	-
CBR3	13298	-0.001	0.1241	-
CPOX	13311	-0.001	0.1239	-
ECI2	13385	-0.002	0.121	-
YWHAH	13436	-0.002	0.1192	-
PPARA	13481	-0.002	0.1177	-
HSPH1	13606	-0.003	0.1129	-
ACSL4	13981	-0.005	0.0977	-
ACOX1	13987	-0.005	0.0984	-
PDHA1	14054	-0.005	0.0966	-
CEL	14319	-0.007	0.0866	-
ALDOA	14433	-0.007	0.0832	-
MDH2	14477	-0.008	0.0828	-
ALDH9A1	14516	-0.008	0.0827	-
CD36	14530	-0.008	0.0837	-
LDHA	14863	-0.01	0.0713	-
EHHADH	14866	-0.01	0.0732	-
ALDH3A2	14879	-0.01	0.0746	-
TP53INP2	14904	-0.01	0.0756	-
HAO2	15082	-0.011	0.0702	-

NCAPH2	15178	-0.012	0.0684	-
ADSL	15403	-0.013	0.0613	-
MDH1	15580	-0.014	0.0564	-
INMT	15737	-0.015	0.0525	-
LTC4S	15915	-0.016	0.048	-
GRHPR	15985	-0.016	0.0482	-
RAP1GDS1	16029	-0.016	0.0495	-
FABP2	16117	-0.016	0.049	-
AADAT	16205	-0.017	0.0486	-
GPD2	16259	-0.017	0.0497	-
APEX1	16484	-0.018	0.0437	-
D2HGDH	16537	-0.019	0.0451	-
S100A10	16555	-0.019	0.048	-
GAD2	16593	-0.019	0.0501	-
RDH11	16674	-0.019	0.0504	-
PCBD1	16902	-0.021	0.0448	-
ECH1	17168	-0.023	0.0378	-
UGDH	17175	-0.023	0.042	-
SDHD	17213	-0.023	0.0449	-
PSME1	17286	-0.023	0.0464	-
ALDH3A1	17325	-0.024	0.0494	-
CA4	17653	-0.026	0.0404	-
SDHC	17850	-0.027	0.0373	-
REEP6	18585	-0.033	0.0121	-
SMS	18778	-0.034	0.0105	-
HSD17B7	18781	-0.034	0.0172	-
ALAD	18793	-0.034	0.0234	-
ACSL5	18908	-0.035	0.0254	-
UBE2L6	19114	-0.037	0.0239	-
UROD	19185	-0.037	0.0282	-
CRAT	19318	-0.039	0.0301	-
ACOT2	19354	-0.039	0.0363	-
CPT1A	19364	-0.039	0.0436	-
ENO2	19461	-0.04	0.0473	-
CD1D	19616	-0.042	0.0488	-
DECR1	19819	-0.043	0.0487	-
PTS	20107	-0.046	0.0454	-
NTHL1	20191	-0.047	0.0511	-
RDH16	20432	-0.05	0.0506	-
HCCS	20671	-0.053	0.0508	-
G0S2	21044	-0.059	0.0463	-

CA2	21357	-0.063	0.0454	-
MIX23	21828	-0.073	0.0396	-
IL4I1	23215	-0.145	0.0085	-

Table S7. Gene set enrichment analysis details for HALLMARK_ OXIDATIVE_PHOSPHORYLATION gene set comparing healthy Ctrl and GDM maternal peripheral blood mononuclear cells (PBMCs).

Gene symbol	Rank in gene list	Rank metric score	Running enrichment score	Enrichment
RETSAT	11	1.073	0.0158	↑ Ctrl
IDH3B	33	0.961	0.0289	↑ Ctrl
NDUFV1	91	0.859	0.0373	↑ Ctrl
POR	148	0.814	0.0451	↑ Ctrl
PDP1	395	0.711	0.0344	↑ Ctrl
UQCRC1	399	0.71	0.0452	↑ Ctrl
SLC25A4	400	0.71	0.0563	↑ Ctrl
UQCRB	470	0.689	0.0609	↑ Ctrl
HCCS	581	0.663	0.0616	↑ Ctrl
ECHS1	640	0.652	0.0666	↑ Ctrl
NDUFB7	720	0.635	0.0695	↑ Ctrl
FDX1	729	0.633	0.0787	↑ Ctrl
GOT2	802	0.62	0.082	↑ Ctrl
SUCLA2	857	0.611	0.0868	↑ Ctrl
ATP6V1H	866	0.61	0.0956	↑ Ctrl
POLR2F	960	0.595	0.0966	↑ Ctrl
MRPS22	989	0.59	0.1034	↑ Ctrl
PHB2	995	0.589	0.1121	↑ Ctrl
HSD17B10	1011	0.587	0.1199	↑ Ctrl
ACAA1	1016	0.586	0.1287	↑ Ctrl
PDHA1	1022	0.586	0.1375	↑ Ctrl
SUCLG1	1029	0.585	0.1461	↑ Ctrl
MTRR	1079	0.577	0.1507	↑ Ctrl
RHOT2	1124	0.572	0.1558	↑ Ctrl
ETFDH	1162	0.567	0.1613	↑ Ctrl
AIFM1	1210	0.562	0.166	↑ Ctrl
MDH2	1255	0.557	0.1708	↑ Ctrl
ISCU	1329	0.551	0.1729	↑ Ctrl
NDUFA9	1356	0.548	0.1792	↑ Ctrl
ACAT1	1407	0.544	0.1832	↑ Ctrl
ETFB	1419	0.542	0.1907	↑ Ctrl
PDHX	1566	0.526	0.186	↑ Ctrl
ATP6AP1	1626	0.52	0.1889	↑ Ctrl
SLC25A5	1769	0.508	0.1843	↑ Ctrl

ACO2	1813	0.504	0.1883	↑ Ctrl
SDHA	1882	0.499	0.1901	↑ Ctrl
COX11	1920	0.496	0.1946	↑ Ctrl
ATP5MF	1985	0.493	0.1966	↑ Ctrl
TIMM17A	2007	0.491	0.2024	↑ Ctrl
OGDH	2229	0.473	0.1902	↑ Ctrl
IMMT	2272	0.47	0.1939	↑ Ctrl
IDH3G	2305	0.468	0.1983	↑ Ctrl
MRPL35	2308	0.467	0.2055	↑ Ctrl
TIMM50	2313	0.467	0.2124	↑ Ctrl
NDUFS8	2434	0.459	0.2089	↑ Ctrl
ATP5MG	2711	0.441	0.1914	↑ Ctrl
NNT	2739	0.439	0.1959	↑ Ctrl
NDUFB5	2829	0.434	0.1948	↑ Ctrl
HSPA9	2859	0.432	0.199	↑ Ctrl
ALAS1	2883	0.43	0.2036	↑ Ctrl
NDUFS1	2892	0.43	0.2096	↑ Ctrl
OXA1L	2900	0.43	0.2157	↑ Ctrl
NDUFB4	2990	0.424	0.2145	↑ Ctrl
ATP5F1A	2998	0.424	0.2205	↑ Ctrl
OAT	3003	0.423	0.2267	↑ Ctrl
DLD	3045	0.421	0.2297	↑ Ctrl
ACADSB	3152	0.416	0.2268	↑ Ctrl
MPC1	3167	0.415	0.232	↑ Ctrl
AFG3L2	3208	0.412	0.2349	↑ Ctrl
NDUFA1	3222	0.411	0.2402	↑ Ctrl
GRPEL1	3329	0.404	0.2371	↑ Ctrl
MGST3	3349	0.403	0.2417	↑ Ctrl
COX6C	3392	0.401	0.2443	↑ Ctrl
COX10	3415	0.399	0.2485	↑ Ctrl
NDUFV2	3470	0.396	0.25	↑ Ctrl
VDAC1	3553	0.392	0.2488	↑ Ctrl
NDUFA5	3628	0.388	0.2483	↑ Ctrl
NDUFAB1	3689	0.385	0.249	↑ Ctrl
GPI	3720	0.384	0.2524	↑ Ctrl
NDUFC2	3800	0.379	0.2513	↑ Ctrl
LDHB	3844	0.378	0.2534	↑ Ctrl
SDHB	3974	0.371	0.2477	↑ Ctrl
NDUFS4	3982	0.371	0.2529	↑ Ctrl
COX5B	4029	0.368	0.2546	↑ Ctrl
ETFA	4073	0.366	0.2565	↑ Ctrl

MTRF1	4078	0.366	0.2619	↑ Ctrl
UQCRH	4080	0.366	0.2675	↑ Ctrl
RHOT1	4177	0.361	0.2646	↑ Ctrl
SUPV3L1	4184	0.361	0.2697	↑ Ctrl
CASP7	4218	0.359	0.2724	↑ Ctrl
ATP6V1D	4262	0.357	0.2742	↑ Ctrl
GLUD1	4345	0.353	0.2724	↑ Ctrl
IDH1	4384	0.351	0.2745	↑ Ctrl
VDAC2	4407	0.35	0.278	↑ Ctrl
TIMM8B	4449	0.348	0.2799	↑ Ctrl
DECR1	4535	0.344	0.2777	↑ Ctrl
HTRA2	4552	0.343	0.2817	↑ Ctrl
SDHD	4598	0.341	0.283	↑ Ctrl
ATP5MC2	4657	0.339	0.2832	↑ Ctrl
COX17	4669	0.338	0.2875	↑ Ctrl
NDUFS2	4744	0.335	0.2861	↑ Ctrl
TOMM70	4781	0.334	0.2882	↑ Ctrl
PRDX3	4799	0.333	0.2919	↑ Ctrl
ACADM	4807	0.332	0.2964	↑ Ctrl
TCIRG1	4978	0.325	0.2865	↑ Ctrl
MRPS15	5028	0.324	0.2872	↑ Ctrl
SLC25A3	5067	0.322	0.2889	↑ Ctrl
UQCR11	5075	0.322	0.2933	↑ Ctrl
FH	5099	0.321	0.2962	↑ Ctrl
PMPCA	5123	0.32	0.2992	↑ Ctrl
COX15	5187	0.318	0.2986	↑ Ctrl
ABCB7	5247	0.315	0.2983	↑ Ctrl
CS	5252	0.315	0.3028	↑ Ctrl
LRPPRC	5288	0.313	0.3046	↑ Ctrl
NDUFS6	5332	0.312	0.3057	↑ Ctrl
COX4I1	5601	0.301	0.2867	-
MDH1	5659	0.298	0.2863	-
HADHB	5663	0.298	0.2907	-
PDHB	5665	0.298	0.2952	-
ATP5PB	5693	0.297	0.2975	-
NDUFB8	5804	0.294	0.2923	-
CYC1	5877	0.291	0.2905	-
NDUFB6	6176	0.279	0.2685	-
NDUFS3	6190	0.279	0.2717	-
ATP5PF	6196	0.279	0.2756	-
COX7A2L	6222	0.277	0.2777	-

ATP5MC1	6226	0.277	0.2818	-
ATP6V1E1	6240	0.276	0.285	-
UQCRC2	6274	0.275	0.2863	-
ECH1	6391	0.27	0.2803	-
COX5A	6428	0.269	0.2813	-
BCKDHA	6432	0.269	0.2852	-
SDHC	6443	0.268	0.2885	-
ATP5F1C	6445	0.268	0.2926	-
ATP6V1F	6485	0.267	0.2933	-
DLST	6531	0.265	0.2935	-
NQO2	6719	0.257	0.281	-
CYCS	6747	0.256	0.2826	-
CYB5R3	6758	0.255	0.2857	-
ALDH6A1	6811	0.253	0.285	-
ATP5PO	6821	0.253	0.2882	-
NDUFA6	6919	0.249	0.2835	-
ATP5F1B	7254	0.236	0.2576	-
SLC25A12	7283	0.235	0.2588	-
ACAA2	7364	0.232	0.2553	-
TIMM9	7393	0.23	0.2564	-
LDHA	7423	0.23	0.2574	-
NDUFB3	7551	0.225	0.2497	-
UQCRFS1	7706	0.219	0.2395	-
UQCR10	7716	0.218	0.2421	-
NDUFA2	7788	0.216	0.2392	-
MRPS30	7973	0.209	0.2262	-
ACADVL	8109	0.203	0.2174	-
ATP5PD	8149	0.202	0.2171	-
HADHA	8365	0.193	0.2011	-
NDUFA4	8411	0.191	0.2001	-
ATP6V1C1	8483	0.189	0.1968	-
FXN	8541	0.186	0.1946	-
DLAT	8585	0.185	0.1937	-
SLC25A6	8652	0.182	0.1907	-
IDH3A	8757	0.179	0.1843	-
ATP6V1G1	8768	0.178	0.1862	-
IDH2	8993	0.168	0.169	-
ATP5MC3	9076	0.165	0.1643	-
NDUFB1	9230	0.158	0.1532	-
ATP6V0E1	9329	0.152	0.147	-
NDUFS7	9348	0.151	0.1477	-

NDUFA7	9425	0.147	0.1433	-
OPA1	9527	0.142	0.1366	-
COX6A1	9532	0.141	0.1384	-
TOMM22	9867	0.124	0.1108	-
COX8A	9873	0.124	0.1123	-
ATP6V0B	9915	0.121	0.1105	-
COX6B1	9952	0.118	0.1092	-
ATP6V0C	9982	0.116	0.1085	-
PDK4	10000	0.115	0.1088	-
ISCA1	10024	0.114	0.1085	-
VDAC3	10032	0.113	0.1096	-
NDUFB2	10088	0.109	0.1065	-
SLC25A11	10123	0.105	0.1051	-
BAX	10284	0.093	0.0924	-
GPX4	10288	0.092	0.0936	-
NDUFC1	10289	0.092	0.095	-
MFN2	10317	0.089	0.094	-
NDUFA3	10503	0.071	0.0787	-
CPT1A	10545	0.066	0.0761	-
MTX2	10558	0.064	0.0761	-
MRPL34	10581	0.062	0.0751	-
ATP5F1D	10767	0.036	0.0593	-
NDUFA8	10948	0.005	0.0435	-
UQCRQ	11026	-0.011	0.0368	-
ATP5F1E	11027	-0.011	0.037	-
SURF1	11049	-0.015	0.0354	-
COX7C	11056	-0.017	0.0351	-
COX7A2	11073	-0.022	0.034	-
MRPS11	11210	-0.069	0.0231	-
SLC25A20	11253	-0.087	0.0207	-

Table S8. Gene set enrichment analysis details for HALLMARK_FATTY_ACID_METABOLISM gene set comparing healthy Ctrl and GDM maternal peripheral blood mononuclear cells (PBMCs).

Gene symbol	Rank in gene list	Rank metric score	Running enrichment score	Enrichment
RETSAT	11	1.073	0.0264	↑ Ctrl
HADH	14	1.039	0.0527	↑ Ctrl
MLYCD	24	1.005	0.0776	↑ Ctrl
IDH3B	33	0.961	0.1014	↑ Ctrl
BLVRA	69	0.883	0.1208	↑ Ctrl
UGDH	120	0.833	0.1376	↑ Ctrl
RAP1GDS1	233	0.766	0.1473	↑ Ctrl

ACSS1	252	0.759	0.1651	↑ Ctrl
HCCS	581	0.663	0.1532	↑ Ctrl
ECHS1	640	0.652	0.1647	↑ Ctrl
SUCLA2	857	0.611	0.1613	↑ Ctrl
HSD17B10	1011	0.587	0.1628	↑ Ctrl
ACAA1	1016	0.586	0.1774	↑ Ctrl
PDHA1	1022	0.586	0.1919	↑ Ctrl
SUCLG1	1029	0.585	0.2063	↑ Ctrl
ENO2	1132	0.57	0.2119	↑ Ctrl
ETFDH	1162	0.567	0.2238	↑ Ctrl
CPT2	1173	0.565	0.2373	↑ Ctrl
MDH2	1255	0.557	0.2444	↑ Ctrl
EPHX1	1305	0.553	0.2542	↑ Ctrl
IDI1	1455	0.538	0.2549	↑ Ctrl
HMGCL	1576	0.525	0.2577	↑ Ctrl
METAP1	1596	0.523	0.2694	↑ Ctrl
PPARA	1637	0.52	0.2791	↑ Ctrl
ACAT2	1639	0.519	0.2923	↑ Ctrl
GRHPR	1799	0.505	0.2912	↑ Ctrl
ACO2	1813	0.504	0.3029	↑ Ctrl
SDHA	1882	0.499	0.3096	↑ Ctrl
SUCLG2	2126	0.481	0.3005	↑ Ctrl
APEX1	2179	0.477	0.3081	↑ Ctrl
ACSL5	2274	0.47	0.3118	↑ Ctrl
IDH3G	2305	0.468	0.3211	↑ Ctrl
ALDH1A1	2826	0.434	0.2865	↑ Ctrl
IL4I1	2908	0.429	0.2903	↑ Ctrl
ALDH3A2	2914	0.429	0.3008	↑ Ctrl
HIBCH	3007	0.423	0.3035	↑ Ctrl
DLD	3045	0.421	0.311	↑ Ctrl
UBE2L6	3064	0.42	0.3202	↑ Ctrl
RDH11	3401	0.4	0.3008	↑ Ctrl
UROS	3682	0.385	0.286	↑ Ctrl
SLC22A5	3799	0.379	0.2855	↑ Ctrl
ALDOA	4005	0.369	0.2769	↑ Ctrl
KMT5A	4146	0.362	0.2739	↑ Ctrl
ERP29	4329	0.354	0.2669	↑ Ctrl
MIF	4342	0.353	0.2748	↑ Ctrl
IDH1	4384	0.351	0.2802	↑ Ctrl
HSD17B4	4458	0.348	0.2826	↑ Ctrl
ADSL	4518	0.345	0.2862	↑ Ctrl

DECR1	4535	0.344	0.2936	↑ Ctrl
SDHD	4598	0.341	0.2969	↑ Ctrl
YWHAH	4650	0.339	0.301	↑ Ctrl
ECI2	4726	0.336	0.303	↑ Ctrl
ACADM	4807	0.332	0.3044	↑ Ctrl
FH	5099	0.321	0.287	↑ Ctrl
NBN	5195	0.317	0.2868	↑ Ctrl
NCAPH2	5207	0.317	0.2939	↑ Ctrl
S100A10	5223	0.316	0.3006	↑ Ctrl
ALDH9A1	5585	0.302	0.2766	↑ Ctrl
MGLL	5598	0.301	0.2832	↑ Ctrl
CD1D	5640	0.299	0.2873	↑ Ctrl
PSME1	5650	0.299	0.2941	↑ Ctrl
HPGD	5655	0.299	0.3013	↑ Ctrl
MDH1	5659	0.298	0.3087	↑ Ctrl
HADHB	5663	0.298	0.316	↑ Ctrl
PDHB	5665	0.298	0.3236	↑ Ctrl
HSPH1	5701	0.297	0.3281	↑ Ctrl
GPD2	5850	0.292	0.3225	-
ECH1	6391	0.27	0.2819	-
CPOX	6401	0.27	0.288	-
SDHC	6443	0.268	0.2912	-
DLST	6531	0.265	0.2904	-
CBR1	6625	0.261	0.2888	-
ADIPOR2	6657	0.259	0.2927	-
HSP90AA1	6667	0.259	0.2986	-
ACAA2	7364	0.232	0.2433	-
LDHA	7423	0.23	0.244	-
HSD17B11	7458	0.228	0.2469	-
HSDL2	7621	0.222	0.2383	-
ACSL4	8106	0.204	0.201	-
ACADVL	8109	0.203	0.206	-
ELOVL5	8258	0.197	0.198	-
CD36	8270	0.197	0.202	-
SMS	8569	0.185	0.1806	-
AUH	8657	0.182	0.1776	-
H2AZ1	8680	0.181	0.1803	-
BCKDHB	8960	0.17	0.1601	-
LGALS1	9186	0.16	0.1444	-
XIST	9297	0.154	0.1386	-
ACSL1	9475	0.144	0.1268	-

VNN1	9484	0.144	0.1297	-
ODC1	9577	0.139	0.1252	-
CA4	9625	0.137	0.1246	-
SERINC1	9833	0.126	0.1096	-
ACOX1	10064	0.11	0.0922	-
GABARAPL1	10069	0.11	0.0946	-
HSD17B7	10248	0.095	0.0814	-
CRAT	10391	0.083	0.0711	-
GLUL	10398	0.082	0.0726	-
PRDX6	10438	0.078	0.0712	-
ALAD	10446	0.078	0.0726	-
HMGCS1	10520	0.069	0.0679	-
CPT1A	10545	0.066	0.0675	-
TP53INP2	10585	0.062	0.0656	-
AOC3	10607	0.058	0.0653	-
CRYZ	10953	0.004	0.0351	-
OSTC	11207	-0.066	0.0145	-
ACOT8	11251	-0.085	0.0129	-
CA2	11336	-0.141	0.0091	-
UROD	11356	-0.158	0.0115	-

Table S9. Gene set enrichment analysis details for HALLMARK_INFLAMMATORY_RESPONSE gene set comparing healthy Ctrl and GDM maternal subcutaneous fat.

Gene symbol	Rank in gene list	Rank metric score	Running enrichment score	Enrichment
CSF3	10	0.295	0.0267	-
SELE	29	0.217	0.0458	-
IL6	214	0.133	0.0502	-
CLEC5A	415	0.108	0.0514	-
MET	490	0.101	0.0576	-
F3	594	0.094	0.0618	-
INHBA	675	0.09	0.0667	-
OSM	805	0.084	0.0688	-
CDKN1A	906	0.08	0.0719	-
EBI3	997	0.077	0.0751	-
KCNJ2	1020	0.077	0.0812	-
SLC28A2	1109	0.074	0.0843	-
SLC31A2	1200	0.072	0.087	-
SLC4A4	1250	0.071	0.0914	-
CALCRL	1259	0.07	0.0975	-
CCL17	1290	0.07	0.1026	-
IL15RA	1387	0.067	0.1047	-

CX3CL1	1668	0.062	0.0983	-
TLR2	1672	0.062	0.1038	-
CXCL10	1911	0.058	0.0989	-
EREG	1968	0.057	0.1017	-
CXCL9	1997	0.056	0.1056	-
LDLR	2146	0.054	0.1042	-
NFKBIA	2242	0.053	0.1049	-
TNFSF10	2283	0.052	0.108	-
BTG2	2304	0.052	0.1119	-
MYC	2423	0.05	0.1114	-
IL1A	2559	0.048	0.11	-
LCK	3063	0.042	0.0922	-
SRI	3094	0.042	0.0948	-
OPRK1	3162	0.041	0.0957	-
FZD5	3208	0.041	0.0975	-
MEFV	3391	0.039	0.0932	-
ABCA1	3486	0.038	0.0927	-
MSR1	3534	0.038	0.0941	-
IRF1	3872	0.035	0.0828	-
IL7R	4247	0.032	0.0696	-
GP1BA	4715	0.028	0.0521	-
EIF2AK2	4749	0.028	0.0532	-
NDP	5039	0.026	0.0432	-
CXCL11	5073	0.026	0.0441	-
CXCL6	5221	0.025	0.04	-
GABBR1	5880	0.021	0.0136	-
ITGB8	6058	0.019	0.0078	-
KIF1B	6260	0.018	0.0008	-
ACVR2A	6288	0.018	0.0013	-
DCBLD2	6370	0.017	-0.0006	-
CCL20	6500	0.017	-0.0046	-
KLF6	6645	0.016	-0.0093	-
SLC7A2	6668	0.016	-0.0088	-
CXCL8	6821	0.015	-0.0139	-
NFKB1	6903	0.015	-0.0161	-
IFNAR1	7015	0.014	-0.0196	-
RIPK2	7035	0.014	-0.0191	-
ICOSLG	7193	0.013	-0.0247	-
RTP4	7535	0.011	-0.0384	-
IL18RAP	7679	0.01	-0.0436	-
ACVR1B	7748	0.01	-0.0456	-

STAB1	8185	0.008	-0.0637	-
GPC3	8201	0.007	-0.0637	-
AXL	8634	0.005	-0.0818	-
CCL2	9090	0.002	-0.1012	-
IRAK2	9202	0.002	-0.1058	-
CD69	9272	0.001	-0.1087	-
IL18R1	9398	0.001	-0.114	-
ATP2A2	9471	0	-0.1171	-
LTA	10409	0	-0.1575	-
TACR3	11334	0	-0.1973	-
SCN1B	13167	-0.001	-0.2761	-
NAMPT	13267	-0.001	-0.2803	-
TAPBP	13656	-0.003	-0.2968	-
TLR1	13661	-0.003	-0.2967	-
IFNGR2	13801	-0.004	-0.3023	-
SELENOS	13805	-0.004	-0.3021	-
CCL5	14258	-0.006	-0.321	-
RAF1	14565	-0.008	-0.3334	-
ITGA5	15182	-0.012	-0.3589	-
HPN	15237	-0.012	-0.3601	-
IL12B	15366	-0.013	-0.3645	-
NOD2	15390	-0.013	-0.3643	-
SLAMF1	15568	-0.014	-0.3707	-
ADRM1	15695	-0.014	-0.3748	-
PDE4B	15794	-0.015	-0.3776	-
ROS1	16118	-0.016	-0.39	-
SELL	16169	-0.017	-0.3906	-
SPHK1	16199	-0.017	-0.3903	-
CXCR6	16506	-0.018	-0.4018	-
PVR	16725	-0.02	-0.4094	-
MXD1	16828	-0.02	-0.4119	-
GPR183	16853	-0.02	-0.4111	-
ATP2C1	16940	-0.021	-0.4129	-
MEP1A	16960	-0.021	-0.4118	-
CD40	16975	-0.021	-0.4104	-
TACR1	17322	-0.023	-0.4232	-
RGS16	17404	-0.024	-0.4244	-
PDPN	17450	-0.024	-0.4241	-
IL10RA	17466	-0.024	-0.4225	-
TPBG	17662	-0.026	-0.4286	-
RELA	17684	-0.026	-0.4271	-

ABI1	17756	-0.026	-0.4277	-
AHR	17942	-0.028	-0.4332	-
LYN	18072	-0.029	-0.4361	↑ GDM
P2RX7	18122	-0.029	-0.4355	↑ GDM
CD70	18156	-0.029	-0.4343	↑ GDM
CHST2	18160	-0.029	-0.4317	↑ GDM
IL15	18192	-0.03	-0.4303	↑ GDM
IL1R1	18219	-0.03	-0.4287	↑ GDM
BDKRB1	18230	-0.03	-0.4264	↑ GDM
KCNMB2	18240	-0.03	-0.424	↑ GDM
NLRP3	18251	-0.03	-0.4217	↑ GDM
GPR132	18355	-0.031	-0.4233	↑ GDM
RASGRP1	18361	-0.031	-0.4207	↑ GDM
PSEN1	18388	-0.031	-0.419	↑ GDM
SCARF1	18391	-0.031	-0.4163	↑ GDM
TLR3	18644	-0.033	-0.4241	↑ GDM
HRH1	18836	-0.035	-0.4291	↑ GDM
P2RX4	18880	-0.035	-0.4278	↑ GDM
TNFRSF9	19040	-0.036	-0.4313	↑ GDM
CSF1	19112	-0.037	-0.4309	↑ GDM
GCH1	19135	-0.037	-0.4285	↑ GDM
TNFAIP6	19175	-0.037	-0.4268	↑ GDM
BST2	19178	-0.037	-0.4234	↑ GDM
PROK2	19301	-0.038	-0.4251	↑ GDM
LIF	19387	-0.039	-0.4252	↑ GDM
CCRL2	19460	-0.04	-0.4246	↑ GDM
NMI	19551	-0.041	-0.4247	↑ GDM
CD14	19577	-0.041	-0.422	↑ GDM
RNF144B	19613	-0.042	-0.4197	↑ GDM
ADORA2B	19644	-0.042	-0.4172	↑ GDM
EMP3	19704	-0.042	-0.4158	↑ GDM
BEST1	19774	-0.043	-0.4148	↑ GDM
ADM	19776	-0.043	-0.4109	↑ GDM
PTGER2	19875	-0.044	-0.4111	↑ GDM
HIF1A	19910	-0.044	-0.4085	↑ GDM
HBEGF	19955	-0.045	-0.4063	↑ GDM
SLC11A2	20035	-0.046	-0.4055	↑ GDM
MMP14	20043	-0.046	-0.4017	↑ GDM
PTAFR	20111	-0.046	-0.4003	↑ GDM
GNAI3	20315	-0.049	-0.4046	↑ GDM
CD48	20379	-0.049	-0.4027	↑ GDM

SLC1A2	20400	-0.05	-0.399	↑ GDM
RHOG	20668	-0.053	-0.4057	↑ GDM
PCDH7	20762	-0.054	-0.4047	↑ GDM
PTGIR	20766	-0.055	-0.3998	↑ GDM
CMKLR1	20789	-0.055	-0.3957	↑ GDM
ICAM1	20825	-0.055	-0.3921	↑ GDM
SEMA4D	20987	-0.058	-0.3937	↑ GDM
PIK3R5	21098	-0.059	-0.393	↑ GDM
TIMP1	21259	-0.062	-0.3942	↑ GDM
LCP2	21264	-0.062	-0.3887	↑ GDM
CSF3R	21323	-0.063	-0.3855	↑ GDM
IL1B	21392	-0.064	-0.3825	↑ GDM
PTGER4	21411	-0.065	-0.3773	↑ GDM
PTPRE	21427	-0.065	-0.372	↑ GDM
ATP2B1	21460	-0.066	-0.3673	↑ GDM
EDN1	21518	-0.067	-0.3637	↑ GDM
SLC31A1	21548	-0.067	-0.3587	↑ GDM
IFITM1	21570	-0.068	-0.3534	↑ GDM
ICAM4	21584	-0.068	-0.3477	↑ GDM
OSMR	21646	-0.069	-0.344	↑ GDM
TNFRSF1B	21734	-0.071	-0.3412	↑ GDM
CCR7	21778	-0.072	-0.3365	↑ GDM
C3AR1	21799	-0.073	-0.3306	↑ GDM
C5AR1	21902	-0.075	-0.3281	↑ GDM
TNFSF15	21962	-0.077	-0.3236	↑ GDM
FFAR2	21985	-0.077	-0.3175	↑ GDM
IL4R	22008	-0.078	-0.3113	↑ GDM
CYBB	22090	-0.08	-0.3074	↑ GDM
CCL7	22135	-0.081	-0.3018	↑ GDM
IL18	22143	-0.081	-0.2947	↑ GDM
CD55	22258	-0.085	-0.2917	↑ GDM
CD82	22311	-0.087	-0.286	↑ GDM
LY6E	22315	-0.087	-0.2782	↑ GDM
IRF7	22331	-0.088	-0.2708	↑ GDM
IL10	22338	-0.088	-0.263	↑ GDM
NPFFR2	22442	-0.092	-0.259	↑ GDM
VIP	22458	-0.093	-0.2511	↑ GDM
HAS2	22504	-0.094	-0.2444	↑ GDM
FPR1	22532	-0.096	-0.2368	↑ GDM
RGS1	22542	-0.096	-0.2283	↑ GDM
NMUR1	22643	-0.101	-0.2234	↑ GDM

IL2RB	22672	-0.102	-0.2152	↑ GDM
SGMS2	22692	-0.102	-0.2067	↑ GDM
SERPINE1	22804	-0.107	-0.2016	↑ GDM
OLR1	22840	-0.109	-0.193	↑ GDM
MARCO	22842	-0.109	-0.1831	↑ GDM
LPAR1	22881	-0.111	-0.1745	↑ GDM
TNFSF9	22911	-0.113	-0.1654	↑ GDM
KCNA3	23002	-0.12	-0.1582	↑ GDM
PLAUR	23027	-0.122	-0.148	↑ GDM
CCL24	23097	-0.13	-0.139	↑ GDM
ITGB3	23111	-0.132	-0.1275	↑ GDM
AQP9	23145	-0.136	-0.1164	↑ GDM
ADGRE1	23157	-0.137	-0.1042	↑ GDM
GNA15	23181	-0.14	-0.0923	↑ GDM
P2RY2	23276	-0.156	-0.082	↑ GDM
SLC7A1	23367	-0.194	-0.0681	↑ GDM
APLNR	23368	-0.194	-0.0503	↑ GDM
LAMP3	23382	-0.213	-0.0313	↑ GDM
CCL22	23410	-0.355	0.0001	↑ GDM

Table S10. Gene set enrichment analysis details for HALLMARK_GLYCOLYSIS gene set comparing healthy Ctrl and GDM placenta.

Gene symbol	Rank in gene list	Rank metric score	Running enrichment score	Enrichment
COL5A1	251	0.345	0.0052	↑ Ctrl
IL13RA1	496	0.315	0.0093	↑ Ctrl
GAL3ST1	535	0.313	0.0206	↑ Ctrl
HK2	993	0.284	0.0161	↑ Ctrl
VEGFA	1040	0.282	0.0259	↑ Ctrl
SOX9	1065	0.281	0.0363	↑ Ctrl
SDC2	1208	0.275	0.0424	↑ Ctrl
CD44	1381	0.269	0.0472	↑ Ctrl
ENO1	1428	0.267	0.0563	↑ Ctrl
TFF3	1506	0.264	0.0643	↑ Ctrl
AGRN	1547	0.262	0.0734	↑ Ctrl
VCAN	1563	0.262	0.0834	↑ Ctrl
IRS2	1645	0.259	0.091	↑ Ctrl
ALDOA	1825	0.253	0.0949	↑ Ctrl
CHST2	1893	0.251	0.1027	↑ Ctrl
GPR87	1926	0.25	0.1116	↑ Ctrl
PFKP	2031	0.247	0.1179	↑ Ctrl
ME2	2436	0.236	0.1134	↑ Ctrl

CDK1	2465	0.235	0.1219	↑ Ctrl
HS2ST1	2693	0.23	0.1232	↑ Ctrl
TPST1	2738	0.228	0.1309	↑ Ctrl
DDIT4	2927	0.224	0.1333	↑ Ctrl
ENO2	2943	0.223	0.1418	↑ Ctrl
NANP	3157	0.22	0.1432	↑ Ctrl
DLD	3221	0.218	0.1498	↑ Ctrl
GOT2	3321	0.216	0.155	↑ Ctrl
LDHA	3325	0.216	0.1636	↑ Ctrl
RPE	3386	0.215	0.1702	↑ Ctrl
SAP30	3604	0.211	0.1711	↑ Ctrl
PKP2	3703	0.209	0.1761	↑ Ctrl
MET	4032	0.202	0.1728	↑ Ctrl
RRAGD	4043	0.202	0.1806	↑ Ctrl
TGFA	4070	0.202	0.1878	↑ Ctrl
IGFBP3	4279	0.198	0.1885	↑ Ctrl
TKTL1	4397	0.195	0.1923	↑ Ctrl
ISG20	4420	0.195	0.1993	↑ Ctrl
TPI1	4625	0.191	0.1999	↑ Ctrl
TGFBI	4704	0.19	0.2049	↑ Ctrl
TXN	4723	0.19	0.2118	↑ Ctrl
SDC3	4767	0.189	0.2179	↑ Ctrl
IER3	4871	0.187	0.2218	↑ Ctrl
GUSB	4916	0.186	0.2278	↑ Ctrl
EXT1	5153	0.182	0.2269	↑ Ctrl
ARPP19	5172	0.181	0.2336	↑ Ctrl
CLDN3	5217	0.181	0.2393	↑ Ctrl
PGK1	5413	0.178	0.2397	↑ Ctrl
CHST6	5451	0.177	0.2455	↑ Ctrl
B3GAT3	5694	0.173	0.244	↑ Ctrl
UGP2	5784	0.171	0.2478	↑ Ctrl
ME1	5800	0.171	0.2542	↑ Ctrl
HSPA5	5811	0.171	0.2607	↑ Ctrl
PKM	5900	0.169	0.2644	↑ Ctrl
SRD5A3	5917	0.169	0.2706	↑ Ctrl
NOL3	6011	0.168	0.2741	↑ Ctrl
ALG1	6116	0.166	0.2772	↑ Ctrl
MXI1	6126	0.166	0.2835	↑ Ctrl
DCN	6148	0.165	0.2894	↑ Ctrl
PPIA	6191	0.164	0.2946	↑ Ctrl
DSC2	6197	0.164	0.301	↑ Ctrl

AK4	6229	0.164	0.3065	↑ Ctrl
HOMER1	6309	0.163	0.3103	↑ Ctrl
CITED2	6519	0.159	0.3094	↑ Ctrl
CTH	6671	0.156	0.3104	↑ Ctrl
SLC25A10	6685	0.156	0.3162	↑ Ctrl
IDH1	6687	0.156	0.3224	↑ Ctrl
CHST1	6733	0.155	0.3271	↑ Ctrl
CHPF2	6812	0.154	0.3306	↑ Ctrl
SDHC	6942	0.152	0.3322	↑ Ctrl
SLC25A13	6953	0.152	0.3379	↑ Ctrl
CLDN9	7069	0.15	0.34	↑ Ctrl
ALDH9A1	7395	0.145	0.3345	↑ Ctrl
RARS1	7396	0.145	0.3403	↑ Ctrl
GCLC	7720	0.14	0.3347	↑ Ctrl
GYS1	7792	0.139	0.3378	↑ Ctrl
XYLT2	7814	0.139	0.3427	↑ Ctrl
MPI	8262	0.131	0.3324	↑ Ctrl
B4GALT4	8506	0.128	0.3292	↑ Ctrl
EFNA3	8528	0.128	0.3336	↑ Ctrl
HAX1	8885	0.122	0.3261	↑ Ctrl
GALK1	8956	0.121	0.3285	↑ Ctrl
GALK2	9015	0.12	0.3313	↑ Ctrl
P4HA1	9202	0.117	0.3296	↑ Ctrl
MERTK	9280	0.116	0.3316	↑ Ctrl
FBP2	9373	0.115	0.333	↑ Ctrl
ABCB6	9427	0.114	0.3357	↑ Ctrl
ANG	9451	0.113	0.3395	↑ Ctrl
LDHC	9483	0.113	0.3429	↑ Ctrl
CXCR4	9491	0.113	0.3472	↑ Ctrl
PYGL	9497	0.113	0.3516	↑ Ctrl
FAM162A	9514	0.112	0.3555	↑ Ctrl
MIF	9595	0.111	0.3572	↑ Ctrl
PPP2CB	9628	0.111	0.3605	↑ Ctrl
IDUA	9744	0.109	0.3609	↑ Ctrl
SPAG4	9813	0.108	0.3629	↑ Ctrl
PLOD2	9827	0.107	0.3668	↑ Ctrl
ERO1A	9960	0.105	0.3664	↑ Ctrl
STC1	10086	0.103	0.3662	↑ Ctrl
SLC16A3	10146	0.102	0.3683	↑ Ctrl
STC2	10320	0.1	0.3663	-
PGLS	10476	0.097	0.3648	-

PGAM1	10544	0.096	0.3664	-
EGLN3	10928	0.09	0.3567	-
CASP6	11009	0.089	0.3575	-
FKBP4	11295	0.085	0.351	-
CYB5A	11420	0.083	0.3501	-
LCT	11507	0.081	0.3503	-
GNPDA1	11528	0.081	0.3529	-
PRPS1	11622	0.079	0.3528	-
CAPN5	11678	0.078	0.3541	-
CLN6	11742	0.077	0.355	-
COG2	11849	0.076	0.3544	-
AKR1A1	12168	0.071	0.3462	-
ALDH7A1	12335	0.068	0.3432	-
PC	12352	0.068	0.3453	-
CENPA	12357	0.067	0.3479	-
PAM	12550	0.064	0.3438	-
P4HA2	12877	0.059	0.3349	-
SLC35A3	12897	0.059	0.3366	-
VLDLR	13319	0.052	0.3241	-
LHPP	13347	0.052	0.3252	-
PHKA2	13373	0.051	0.3264	-
COPB2	13557	0.048	0.322	-
PLOD1	13779	0.044	0.3161	-
POLR3K	13805	0.044	0.317	-
PPFIA4	14005	0.041	0.3118	-
ANKZF1	14054	0.04	0.3117	-
PMM2	14084	0.04	0.3123	-
NSDHL	14238	0.037	0.3085	-
TPBG	14339	0.036	0.3065	-
NT5E	14587	0.032	0.2992	-
MDH2	14802	0.028	0.2929	-
GPC3	14819	0.028	0.2934	-
G6PD	14844	0.027	0.2937	-
CHST12	14852	0.027	0.2946	-
STMN1	14922	0.026	0.2932	-
ZNF292	14957	0.026	0.2931	-
ECD	15057	0.024	0.2906	-
KDELR3	15154	0.023	0.2882	-
GPC1	15173	0.022	0.2885	-
EXT2	15545	0.017	0.2763	-
PGM2	15691	0.015	0.2719	-

LHX9	15788	0.014	0.2691	-
B4GALT7	15899	0.012	0.2658	-
MDH1	16101	0.01	0.2592	-
B4GALT2	16155	0.009	0.2578	-
GMPPA	16412	0.006	0.2491	-
DPYSL4	16423	0.006	0.249	-
BPNT1	16519	0.004	0.2459	-
HDLBP	16648	0.003	0.2416	-
AK3	16678	0.002	0.2406	-
B3GALT6	16804	0	0.2363	-
TALDO1	20508	-0.009	0.1083	-
CHST4	20597	-0.01	0.1056	-
ALDOB	20922	-0.015	0.095	-
EGFR	21215	-0.018	0.0856	-
HMMR	21312	-0.02	0.0831	-
GAPDHS	21399	-0.021	0.0809	-
B4GALT1	21413	-0.021	0.0813	-
NDUFV3	21479	-0.022	0.0799	-
SLC37A4	21622	-0.024	0.076	-
FUT8	21951	-0.03	0.0658	-
MED24	22006	-0.031	0.0652	-
BIK	22227	-0.036	0.059	-
GLRX	22601	-0.043	0.0478	-
PFKFB1	22621	-0.043	0.0489	-
KIF20A	22942	-0.051	0.0398	-
SOD1	23083	-0.054	0.0371	-
GMPPB	23155	-0.056	0.0369	-
NDST3	23196	-0.058	0.0379	-
GFPT1	23591	-0.067	0.0269	-
GFUS	23938	-0.076	0.0179	-
GPC4	23951	-0.076	0.0206	-
PYGB	24012	-0.078	0.0216	-
CACNA1H	24087	-0.08	0.0222	-
HS6ST2	24125	-0.081	0.0242	-
B3GAT1	24212	-0.083	0.0245	-
CHPF	24653	-0.096	0.0131	-
PSMC4	24732	-0.099	0.0144	-
ANGPTL4	24796	-0.1	0.0162	-
ADORA2B	24850	-0.102	0.0185	-
GOT1	25038	-0.107	0.0163	-
ARTN	25058	-0.108	0.02	-

PAXIP1	25154	-0.111	0.0211	-
AURKA	25289	-0.115	0.0211	-
GYS2	25385	-0.118	0.0225	-
DEPDC1	25428	-0.119	0.0259	-
AGL	25540	-0.123	0.0269	-
PGAM2	25692	-0.128	0.0269	-
RBCK1	25819	-0.133	0.0278	-
GLCE	25868	-0.135	0.0316	-
PDK3	26241	-0.149	0.0247	-
SDC1	26333	-0.152	0.0277	-
MIOX	26668	-0.165	0.0227	-
QSOX1	27177	-0.189	0.0127	-
ELF3	27295	-0.195	0.0164	-
GNE	27535	-0.208	0.0165	-
B3GNT3	27545	-0.209	0.0246	-
NASP	27600	-0.213	0.0312	-
GALE	27820	-0.229	0.0328	-
KIF2A	27988	-0.239	0.0366	-

Table S11. Gene set enrichment analysis details for HALLMARK_OXIDATIVE_PHOSPHORYLATION gene set comparing healthy Ctrl and GDM placenta.

Gene symbol	Rank in gene list	Rank metric score	Running enrichment score	Enrichment
DECR1	191	0.358	0.0084	↑ Ctrl
MRPS30	265	0.343	0.0203	↑ Ctrl
BDH2	418	0.324	0.0287	↑ Ctrl
TIMM9	556	0.31	0.0369	↑ Ctrl
NNT	884	0.289	0.0378	↑ Ctrl
ACAT1	1524	0.263	0.0267	↑ Ctrl
RETSAT	2078	0.246	0.0178	↑ Ctrl
NDUFB5	2185	0.243	0.0243	↑ Ctrl
VDAC1	2227	0.242	0.0331	↑ Ctrl
NDUFA5	2364	0.238	0.0383	↑ Ctrl
MAOB	2617	0.232	0.0393	↑ Ctrl
LRPPRC	2696	0.23	0.0463	↑ Ctrl
ISCA1	2810	0.227	0.0519	↑ Ctrl
CYB5R3	2907	0.224	0.058	↑ Ctrl
ACADSB	2942	0.223	0.0662	↑ Ctrl
OPA1	2994	0.222	0.0738	↑ Ctrl
ACADM	3021	0.222	0.0822	↑ Ctrl
DLD	3221	0.218	0.0844	↑ Ctrl
ATP5MC1	3274	0.217	0.0918	↑ Ctrl

GOT2	3321	0.216	0.0993	↑ Ctrl
LDHA	3325	0.216	0.1082	↑ Ctrl
COX6C	3357	0.216	0.1162	↑ Ctrl
SUPV3L1	3700	0.209	0.1131	↑ Ctrl
ALDH6A1	3831	0.206	0.1173	↑ Ctrl
GPI	3903	0.205	0.1234	↑ Ctrl
HADHB	4041	0.202	0.1272	↑ Ctrl
OXA1L	4059	0.202	0.1351	↑ Ctrl
UQCRB	4135	0.2	0.1409	↑ Ctrl
UQCRC2	4245	0.198	0.1455	↑ Ctrl
PDP1	4412	0.195	0.1479	↑ Ctrl
TIMM17A	4446	0.194	0.1549	↑ Ctrl
DLST	4536	0.193	0.1599	↑ Ctrl
SLC25A12	4686	0.19	0.1628	↑ Ctrl
SUCLG1	4842	0.187	0.1653	↑ Ctrl
ECHS1	4908	0.186	0.1708	↑ Ctrl
NDUFA8	5027	0.184	0.1745	↑ Ctrl
NDUFA9	5088	0.183	0.1801	↑ Ctrl
VDAC2	5096	0.183	0.1875	↑ Ctrl
NDUFB2	5145	0.182	0.1935	↑ Ctrl
NDUFS4	5376	0.178	0.193	↑ Ctrl
CYCS	5489	0.176	0.1965	↑ Ctrl
OAT	5498	0.176	0.2037	↑ Ctrl
HADHA	5521	0.176	0.2103	↑ Ctrl
HCCS	5573	0.175	0.2159	↑ Ctrl
NDUFV2	5596	0.174	0.2224	↑ Ctrl
MRPL35	5642	0.174	0.2282	↑ Ctrl
TIMM50	6158	0.165	0.2172	↑ Ctrl
GLUD1	6370	0.161	0.2167	↑ Ctrl
ATP6V1D	6502	0.159	0.2188	↑ Ctrl
MRPL34	6652	0.156	0.2202	↑ Ctrl
IDH1	6687	0.156	0.2256	↑ Ctrl
NDUFB3	6693	0.156	0.232	↑ Ctrl
TOMM70	6810	0.154	0.2344	↑ Ctrl
SDHC	6942	0.152	0.2363	↑ Ctrl
TIMM8B	7018	0.151	0.24	↑ Ctrl
ATP5PD	7051	0.15	0.2452	↑ Ctrl
HSPA9	7080	0.15	0.2505	↑ Ctrl
NDUFA4	7116	0.149	0.2556	↑ Ctrl
ATP6V1C1	7151	0.149	0.2606	↑ Ctrl
NDUFB8	7189	0.148	0.2656	↑ Ctrl

RHOT1	7415	0.144	0.2638	↑ Ctrl
COX10	7469	0.144	0.268	↑ Ctrl
ACAA2	7517	0.143	0.2724	↑ Ctrl
NDUFAB1	7578	0.142	0.2763	↑ Ctrl
ATP5F1C	7641	0.141	0.28	↑ Ctrl
NDUFA1	7679	0.14	0.2846	↑ Ctrl
VDAC3	7818	0.138	0.2857	↑ Ctrl
ATP5PF	7938	0.137	0.2873	↑ Ctrl
PHYH	8041	0.135	0.2894	↑ Ctrl
AFG3L2	8108	0.134	0.2928	↑ Ctrl
CS	8147	0.133	0.297	↑ Ctrl
PRDX3	8187	0.133	0.3012	↑ Ctrl
COX7A2	8202	0.132	0.3063	↑ Ctrl
ABCB7	8365	0.13	0.3062	↑ Ctrl
ATP5ME	8382	0.13	0.3111	↑ Ctrl
GRPEL1	8436	0.129	0.3146	↑ Ctrl
COX5B	8482	0.128	0.3185	↑ Ctrl
ATP5MC3	8507	0.128	0.323	↑ Ctrl
MTRR	8509	0.128	0.3284	↑ Ctrl
SLC25A3	8584	0.127	0.3311	↑ Ctrl
HTRA2	8877	0.122	0.3261	↑ Ctrl
ATP5F1A	8919	0.122	0.3298	↑ Ctrl
CASP7	8944	0.121	0.3341	↑ Ctrl
NDUFB1	8990	0.121	0.3376	↑ Ctrl
LDHB	9085	0.119	0.3393	↑ Ctrl
UQCRH	9087	0.119	0.3443	↑ Ctrl
HSD17B10	9218	0.117	0.3447	↑ Ctrl
PDK4	9231	0.117	0.3492	↑ Ctrl
COX11	9267	0.116	0.3529	↑ Ctrl
TOMM22	9288	0.116	0.357	↑ Ctrl
SDHD	9320	0.115	0.3608	↑ Ctrl
AIFM1	9329	0.115	0.3654	↑ Ctrl
ATP5MG	9398	0.114	0.3678	↑ Ctrl
ATP5F1E	9435	0.114	0.3713	↑ Ctrl
ATP6V0E1	9577	0.111	0.3711	↑ Ctrl
SDHB	9685	0.11	0.372	↑ Ctrl
NDUFC2	9710	0.11	0.3758	↑ Ctrl
TIMM13	9730	0.109	0.3798	↑ Ctrl
ATP6V1H	9757	0.109	0.3834	↑ Ctrl
COX7B	9817	0.108	0.3859	↑ Ctrl
ATP6V0B	9972	0.105	0.385	↑ Ctrl

ATP1B1	10138	0.102	0.3836	↑ Ctrl
GPX4	10149	0.102	0.3875	↑ Ctrl
ATP6V1G1	10174	0.102	0.391	↑ Ctrl
PDHB	10330	0.099	0.3898	↑ Ctrl
COX15	10351	0.099	0.3932	↑ Ctrl
NDUFS3	10439	0.098	0.3943	↑ Ctrl
MPC1	10603	0.095	0.3927	↑ Ctrl
ISCU	10716	0.094	0.3927	↑ Ctrl
MRPS22	10781	0.093	0.3944	↑ Ctrl
ATP6V0C	10984	0.09	0.3912	-
COX7C	11007	0.089	0.3942	-
ATP5F1B	11244	0.085	0.3896	-
CYB5A	11420	0.083	0.387	-
ATP6V1F	11748	0.077	0.3789	-
NQO2	12058	0.072	0.3712	-
ETFB	12087	0.072	0.3733	-
COX6B1	12156	0.071	0.3739	-
BAX	12179	0.07	0.3761	-
SUCLA2	12326	0.068	0.3739	-
MRPL11	12366	0.067	0.3753	-
SLC25A6	12500	0.065	0.3735	-
NDUFA6	12554	0.064	0.3744	-
NDUFB6	12620	0.063	0.3748	-
MFN2	12635	0.063	0.3769	-
COX7A2L	12833	0.06	0.3726	-
NDUFV1	12927	0.059	0.3719	-
COX6A1	13041	0.057	0.3703	-
ATP5MF	13399	0.051	0.3601	-
BCKDHA	13477	0.049	0.3595	-
NDUFB4	13486	0.049	0.3613	-
CYC1	13489	0.049	0.3633	-
UQCR10	13491	0.049	0.3653	-
COX4I1	13514	0.049	0.3666	-
NDUFA2	13572	0.048	0.3666	-
ATP5PO	13687	0.046	0.3646	-
TIMM10	13736	0.045	0.3648	-
PDHX	14087	0.04	0.3544	-
NDUFS1	14303	0.036	0.3484	-
UQCRQ	14337	0.036	0.3488	-
ACAA1	14442	0.034	0.3466	-
ETFDH	14459	0.034	0.3475	-

NDUFA7	14464	0.034	0.3487	-
COX8A	14599	0.032	0.3454	-
MTRF1	14731	0.029	0.3421	-
MDH2	14802	0.028	0.3409	-
ATP5PB	14969	0.025	0.3362	-
FH	15016	0.025	0.3356	-
NDUFS2	15077	0.024	0.3346	-
MRPS11	15098	0.024	0.3349	-
NDUFS6	15126	0.023	0.3349	-
NDUFS7	15461	0.018	0.3241	-
COX17	15716	0.015	0.3159	-
PHB2	15827	0.013	0.3126	-
SDHA	15841	0.013	0.3127	-
IMMT	15921	0.012	0.3105	-
ATP6V1E1	16064	0.01	0.306	-
MDH1	16101	0.01	0.3052	-
ATP6AP1	16118	0.01	0.305	-
IDH3A	16306	0.007	0.2989	-
ATP5MC2	16337	0.007	0.2981	-
DLAT	16535	0.004	0.2914	-
NDUFC1	16594	0.003	0.2896	-
MRPS15	16750	0.001	0.2842	-
MRPL15	20053	-0.003	0.1699	-
UQCRFS1	20568	-0.01	0.1525	-
UQCRC1	20989	-0.015	0.1386	-
SURF1	21104	-0.017	0.1353	-
POLR2F	21406	-0.021	0.1258	-
NDUFB7	21510	-0.022	0.1231	-
MGST3	21522	-0.023	0.1237	-
CPT1A	21656	-0.025	0.1201	-
MTX2	21867	-0.028	0.114	-
SLC25A5	21944	-0.03	0.1127	-
COX5A	22147	-0.034	0.1071	-
SLC25A20	22423	-0.039	0.0992	-
MRPS12	22722	-0.046	0.0908	-
UQCR11	22756	-0.047	0.0916	-
IDH3B	22994	-0.052	0.0856	-
ETFA	23350	-0.061	0.0759	-
TCIRG1	23368	-0.062	0.0779	-
IDH3G	23386	-0.062	0.0799	-
ATP5F1D	23680	-0.069	0.0726	-

SLC25A11	24321	-0.087	0.0541	-
NDUFS8	24616	-0.095	0.0479	-
FXN	24750	-0.099	0.0474	-
ALAS1	24919	-0.104	0.0459	-
ECI1	25039	-0.107	0.0463	-
RHOT2	25045	-0.108	0.0507	-
NDUFA3	25352	-0.117	0.045	-
ACO2	25581	-0.125	0.0423	-
SLC25A4	25645	-0.127	0.0454	-
FDX1	26072	-0.143	0.0367	-
PMPCA	26283	-0.151	0.0357	-
PDHA1	26364	-0.154	0.0394	-
ECH1	26893	-0.175	0.0285	-
ACADVL	27884	-0.232	0.0039	-
POR	28242	-0.26	0.0024	-
OGDH	28543	-0.295	0.0044	-
IDH2	28630	-0.308	0.0144	-

Table S12. Gene set enrichment analysis details for HALLMARK_FATTY_ACID_METABOLISM gene set comparing healthy Ctrl and GDM placenta.

Gene symbol	Rank in gene list	Rank metric score	Running enrichment score	Enrichment
DECR1	191	0.358	0.0097	↑ Ctrl
ACSL1	605	0.307	0.0093	↑ Ctrl
GLUL	720	0.299	0.0189	↑ Ctrl
RDH16	759	0.297	0.0311	↑ Ctrl
LGALS1	787	0.295	0.0435	↑ Ctrl
HSD17B11	801	0.294	0.0565	↑ Ctrl
NBN	968	0.285	0.0637	↑ Ctrl
ELOVL5	1367	0.269	0.0621	↑ Ctrl
MIX23	1545	0.262	0.0679	↑ Ctrl
CRYZ	1552	0.262	0.0796	↑ Ctrl
HSDL2	1815	0.254	0.0821	↑ Ctrl
ALDOA	1825	0.253	0.0933	↑ Ctrl
HSP90AA1	1851	0.252	0.1039	↑ Ctrl
ODC1	1998	0.248	0.1101	↑ Ctrl
RETSAT	2078	0.246	0.1185	↑ Ctrl
HSPH1	2230	0.241	0.1243	↑ Ctrl
ADH1C	2366	0.238	0.1304	↑ Ctrl
SERINC1	2398	0.237	0.1401	↑ Ctrl
TP53INP2	2451	0.236	0.149	↑ Ctrl
HMGCL	2501	0.234	0.158	↑ Ctrl

ACOT8	2768	0.228	0.1591	↑ Ctrl
YWHAH	2895	0.224	0.165	↑ Ctrl
ENO2	2943	0.223	0.1735	↑ Ctrl
ACADM	3021	0.222	0.1809	↑ Ctrl
PCBD1	3048	0.221	0.1901	↑ Ctrl
SMS	3205	0.219	0.1946	↑ Ctrl
DLD	3221	0.218	0.204	↑ Ctrl
ERP29	3240	0.218	0.2133	↑ Ctrl
RAP1GDS1	3263	0.217	0.2224	↑ Ctrl
IL4I1	3282	0.217	0.2316	↑ Ctrl
S100A10	3312	0.216	0.2405	↑ Ctrl
LDHA	3325	0.216	0.2499	↑ Ctrl
ADSL	3402	0.215	0.257	↑ Ctrl
UBE2L6	3446	0.214	0.2652	↑ Ctrl
CYP4A11	3472	0.214	0.2741	↑ Ctrl
RDH11	3982	0.203	0.2657	↑ Ctrl
HADHB	4041	0.202	0.2729	↑ Ctrl
PPARA	4136	0.2	0.2788	↑ Ctrl
EHHADH	4501	0.193	0.2749	↑ Ctrl
ALDH3A2	4523	0.193	0.283	↑ Ctrl
DLST	4536	0.193	0.2913	↑ Ctrl
BPHL	4746	0.189	0.2927	↑ Ctrl
SUCLG1	4842	0.187	0.2979	↑ Ctrl
CBR3	4878	0.187	0.3052	↑ Ctrl
CYP1A1	4887	0.187	0.3134	↑ Ctrl
ECHS1	4908	0.186	0.3212	↑ Ctrl
ADIPOR2	5230	0.181	0.3182	↑ Ctrl
PSME1	5284	0.18	0.3246	↑ Ctrl
IDI1	5460	0.177	0.3266	↑ Ctrl
INMT	5483	0.176	0.3338	↑ Ctrl
HCCS	5573	0.175	0.3387	↑ Ctrl
CD1D	5708	0.173	0.3419	↑ Ctrl
ME1	5800	0.171	0.3465	↑ Ctrl
PTS	5996	0.168	0.3474	↑ Ctrl
GPD2	6079	0.166	0.3521	↑ Ctrl
SUCLG2	6222	0.164	0.3546	↑ Ctrl
CA4	6440	0.16	0.3544	↑ Ctrl
ACSL5	6618	0.157	0.3554	↑ Ctrl
BLVRA	6629	0.157	0.3622	↑ Ctrl
IDH1	6687	0.156	0.3673	↑ Ctrl
APEX1	6724	0.155	0.3731	↑ Ctrl

SDHC	6942	0.152	0.3725	↑ Ctrl
CPOX	7087	0.15	0.3743	↑ Ctrl
UGDH	7381	0.145	0.3707	↑ Ctrl
ALDH9A1	7395	0.145	0.3768	↑ Ctrl
G0S2	7504	0.143	0.3796	↑ Ctrl
ACAA2	7517	0.143	0.3857	↑ Ctrl
METAP1	7829	0.138	0.3812	↑ Ctrl
CD36	7843	0.138	0.387	↑ Ctrl
UROS	8258	0.131	0.3786	↑ Ctrl
H2AZ1	8319	0.131	0.3825	↑ Ctrl
MAOA	8546	0.127	0.3805	↑ Ctrl
AUH	8766	0.124	0.3785	↑ Ctrl
ALDH1A1	8771	0.124	0.384	↑ Ctrl
HIBCH	8800	0.123	0.3886	↑ Ctrl
ACSM3	8911	0.122	0.3903	↑ Ctrl
HSD17B10	9218	0.117	0.385	-
SDHD	9320	0.115	0.3868	-
MIF	9595	0.111	0.3824	-
ACAT2	9724	0.109	0.3829	-
ECI2	9882	0.107	0.3823	-
HADH	10266	0.1	0.3736	-
PDHB	10330	0.099	0.3759	-
EPHX1	10792	0.093	0.3642	-
HSD17B4	10848	0.092	0.3664	-
OSTC	11075	0.088	0.3626	-
AOC3	11177	0.087	0.3631	-
D2HGDH	11180	0.086	0.3669	-
BMPR1B	11563	0.08	0.3574	-
HAO2	11832	0.076	0.3515	-
SUCLA2	12326	0.068	0.3376	-
AADAT	12339	0.068	0.3402	-
XIST	12374	0.067	0.3421	-
REEP6	12678	0.062	0.3344	-
NTHL1	13356	0.051	0.3133	-
VNN1	13402	0.051	0.3141	-
ACSL4	13523	0.048	0.3121	-
HMGCS1	13744	0.045	0.3066	-
LTC4S	13907	0.042	0.3029	-
NSDHL	14238	0.037	0.2931	-
ACAA1	14442	0.034	0.2877	-
ETFDH	14459	0.034	0.2887	-

MDH2	14802	0.028	0.2781	-
GRHPR	14930	0.026	0.2749	-
FH	15016	0.025	0.2731	-
HSD17B7	15302	0.02	0.2641	-
MGLL	15450	0.018	0.2599	-
SDHA	15841	0.013	0.247	-
MDH1	16101	0.01	0.2384	-
UROD	16136	0.009	0.2377	-
MLYCD	16351	0.007	0.2306	-
GAD2	16465	0.005	0.2269	-
FABP2	16487	0.005	0.2264	-
CIDEA	17973	0	0.175	-
ADH7	18622	0	0.1526	-
FABP1	19791	0	0.1121	-
BCKDHB	20823	-0.013	0.077	-
ALAD	20844	-0.013	0.077	-
CYP4A22	20981	-0.015	0.0729	-
GAPDHS	21399	-0.021	0.0595	-
GCDH	21519	-0.023	0.0564	-
CPT1A	21656	-0.025	0.0528	-
ACADS	21980	-0.03	0.043	-
GABARAPL1	22108	-0.033	0.0401	-
CA6	22321	-0.037	0.0344	-
FASN	22526	-0.041	0.0293	-
KMT5A	22551	-0.042	0.0303	-
PTPRG	22709	-0.046	0.027	-
MCEE	22937	-0.051	0.0214	-
IDH3B	22994	-0.052	0.0219	-
PRDX6	23254	-0.059	0.0156	-
IDH3G	23386	-0.062	0.0138	-
ACOX1	23436	-0.063	0.015	-
GSTZ1	23773	-0.071	0.0066	-
CA2	24122	-0.08	-0.0018	-
HPGD	24127	-0.081	0.0017	-
CBR1	24682	-0.097	-0.013	-
FMO1	24825	-0.101	-0.0134	-
TDO2	24843	-0.101	-0.0094	-
ENO3	24849	-0.102	-0.0049	-
ECI1	25039	-0.107	-0.0066	-
CRAT	25353	-0.117	-0.0121	-
SLC22A5	25384	-0.118	-0.0078	-

ACO2	25581	-0.125	-0.0089	-
GPD1	25689	-0.128	-0.0068	-
CPT2	26084	-0.144	-0.0139	-
NCAPH2	26316	-0.152	-0.015	-
PDHA1	26364	-0.154	-0.0096	-
ECH1	26893	-0.175	-0.02	-
DHCR24	27172	-0.188	-0.021	-
ALDH3A1	27259	-0.193	-0.0152	-
CEL	27594	-0.212	-0.0172	-
ACSS1	27608	-0.213	-0.0079	-
HMGCS2	27870	-0.231	-0.0064	-
ACADVL	27884	-0.232	0.0037	-
ACOT2	28182	-0.255	0.005	-
AQP7	28347	-0.27	0.0116	-
ACADL	28399	-0.276	0.0224	-

Table S13. Gene set enrichment analysis details for HALLMARK_INFLAMMATORY_RESPONSE gene set comparing healthy Ctrl and GDM placenta.

Gene symbol	Rank in gene list	Rank metric score	Running enrichment score	Enrichment
GPR183	68	0.408	0.0097	↑ Ctrl
MSR1	113	0.381	0.0194	↑ Ctrl
RGS16	144	0.372	0.0293	↑ Ctrl
TLR2	155	0.369	0.0399	↑ Ctrl
CSF3	189	0.358	0.0493	↑ Ctrl
LPAR1	228	0.349	0.0583	↑ Ctrl
TNFSF9	328	0.334	0.0647	↑ Ctrl
CD40	372	0.329	0.0729	↑ Ctrl
BEST1	387	0.327	0.0821	↑ Ctrl
CD69	449	0.32	0.0894	↑ Ctrl
CX3CL1	524	0.313	0.0961	↑ Ctrl
CYBB	652	0.303	0.1006	↑ Ctrl
NAMPT	683	0.301	0.1084	↑ Ctrl
CLEC5A	740	0.297	0.1153	↑ Ctrl
NOD2	748	0.297	0.1238	↑ Ctrl
KLF6	820	0.293	0.13	↑ Ctrl
CXCL8	874	0.289	0.1367	↑ Ctrl
IRF7	905	0.288	0.1441	↑ Ctrl
CCL2	908	0.288	0.1525	↑ Ctrl
IL15	920	0.287	0.1606	↑ Ctrl
SPHK1	950	0.286	0.168	↑ Ctrl
HIF1A	985	0.284	0.1752	↑ Ctrl

C5AR1	1098	0.279	0.1796	↑ Ctrl
RGS1	1111	0.279	0.1874	↑ Ctrl
C3AR1	1120	0.278	0.1953	↑ Ctrl
DCBLD2	1214	0.275	0.2002	↑ Ctrl
PDE4B	1277	0.272	0.2061	↑ Ctrl
CD14	1293	0.272	0.2136	↑ Ctrl
NMUR1	1482	0.265	0.2149	↑ Ctrl
RTP4	1500	0.264	0.2221	↑ Ctrl
CALCRL	1587	0.261	0.2268	↑ Ctrl
F3	1624	0.26	0.2332	↑ Ctrl
CCRL2	1668	0.258	0.2393	↑ Ctrl
CHST2	1893	0.251	0.239	↑ Ctrl
IL7R	1900	0.251	0.2462	↑ Ctrl
MYC	1911	0.251	0.2532	↑ Ctrl
HBEGF	1928	0.25	0.26	↑ Ctrl
PLAUR	1942	0.25	0.2669	↑ Ctrl
ROS1	2043	0.247	0.2707	↑ Ctrl
KIF1B	2088	0.246	0.2765	↑ Ctrl
ITGB8	2109	0.245	0.283	↑ Ctrl
TLR1	2159	0.243	0.2885	↑ Ctrl
IL10	2165	0.243	0.2955	↑ Ctrl
OSM	2187	0.243	0.3019	↑ Ctrl
PTPRE	2214	0.242	0.3081	↑ Ctrl
EREG	2368	0.238	0.3098	↑ Ctrl
GNAI3	2418	0.237	0.3151	↑ Ctrl
PTAFR	2449	0.236	0.321	↑ Ctrl
BTG2	2590	0.232	0.323	↑ Ctrl
LCP2	2735	0.229	0.3248	↑ Ctrl
CMKLR1	2756	0.228	0.3308	↑ Ctrl
ICAM1	2820	0.227	0.3353	↑ Ctrl
ACVR1B	2851	0.226	0.3409	↑ Ctrl
TNFRSF1B	3059	0.221	0.3403	↑ Ctrl
EIF2AK2	3198	0.219	0.3419	↑ Ctrl
GP1BA	3271	0.217	0.3458	↑ Ctrl
EMP3	3277	0.217	0.3521	↑ Ctrl
SEMA4D	3290	0.217	0.358	↑ Ctrl
IFNGR2	3504	0.213	0.3569	↑ Ctrl
ATP2B1	3547	0.212	0.3617	↑ Ctrl
CXCL6	3559	0.212	0.3676	↑ Ctrl
PCDH7	3599	0.211	0.3725	↑ Ctrl
AXL	3729	0.208	0.3741	↑ Ctrl

CD82	3799	0.207	0.3778	↑ Ctrl
OSMR	3910	0.205	0.3801	↑ Ctrl
GCH1	3938	0.204	0.3852	↑ Ctrl
MET	4032	0.202	0.3879	↑ Ctrl
AQP9	4107	0.201	0.3913	↑ Ctrl
IL1A	4128	0.201	0.3965	↑ Ctrl
ADGRE1	4145	0.2	0.4018	↑ Ctrl
ABCA1	4218	0.199	0.4052	↑ Ctrl
SELENOS	4346	0.196	0.4066	↑ Ctrl
CSF1	4393	0.195	0.4108	↑ Ctrl
MMP14	4472	0.194	0.4138	↑ Ctrl
SERPINE1	4493	0.194	0.4188	↑ Ctrl
ATP2C1	4663	0.19	0.4185	↑ Ctrl
CDKN1A	4766	0.189	0.4206	↑ Ctrl
IFITM1	4834	0.188	0.4238	↑ Ctrl
CXCL9	5033	0.184	0.4223	↑ Ctrl
IL18	5115	0.183	0.4249	↑ Ctrl
NFKB1	5120	0.183	0.4302	↑ Ctrl
CXCL11	5126	0.182	0.4354	↑ Ctrl
IL1B	5177	0.181	0.439	↑ Ctrl
NLRP3	5205	0.181	0.4434	↑ Ctrl
LAMP3	5226	0.181	0.448	↑ Ctrl
TIMP1	5304	0.179	0.4506	↑ Ctrl
ATP2A2	5321	0.179	0.4554	↑ Ctrl
CXCL10	5571	0.175	0.4519	↑ Ctrl
IFNAR1	5627	0.174	0.4551	↑ Ctrl
NMI	5677	0.173	0.4585	↑ Ctrl
NDP	5707	0.173	0.4626	↑ Ctrl
FPR1	5997	0.168	0.4575	↑ Ctrl
IRAK2	5999	0.168	0.4624	↑ Ctrl
HRH1	6034	0.167	0.4662	↑ Ctrl
CD48	6214	0.164	0.4648	↑ Ctrl
LYN	6313	0.162	0.4662	↑ Ctrl
ACVR2A	6318	0.162	0.4709	↑ Ctrl
GNA15	6332	0.162	0.4752	↑ Ctrl
SLC31A1	6352	0.161	0.4793	↑ Ctrl
CD55	6538	0.158	0.4775	↑ Ctrl
LY6E	6769	0.155	0.4741	↑ Ctrl
RIPK2	6977	0.151	0.4714	↑ Ctrl
ABI1	7020	0.151	0.4744	↑ Ctrl
ICOSLG	7089	0.15	0.4765	↑ Ctrl

HPN	7155	0.148	0.4786	↑ Ctrl
PDPN	7280	0.147	0.4786	↑ Ctrl
IL4R	7328	0.146	0.4813	↑ Ctrl
IL1R1	7345	0.145	0.485	↑ Ctrl
PTGER2	7484	0.143	0.4844	↑ Ctrl
CCR7	7537	0.142	0.4868	↑ Ctrl
TNFSF15	7594	0.142	0.4891	↑ Ctrl
FZD5	7868	0.138	0.4837	↑ Ctrl
TACR1	7880	0.137	0.4873	↑ Ctrl
SLC7A1	7898	0.137	0.4908	↑ Ctrl
LCK	7985	0.136	0.4918	↑ Ctrl
SELL	7997	0.136	0.4954	↑ Ctrl
BDKRB1	8071	0.134	0.4969	↑ Ctrl
IL18R1	8122	0.133	0.4991	↑ Ctrl
TAPBP	8148	0.133	0.5021	↑ Ctrl
NFKBIA	8194	0.132	0.5045	↑ Ctrl
PSEN1	8395	0.129	0.5014	-
CXCR6	8523	0.128	0.5007	-
IL15RA	8532	0.128	0.5042	-
SGMS2	8859	0.122	0.4965	-
ADM	9326	0.115	0.4838	-
MARCO	9884	0.107	0.4676	-
SLC11A2	10040	0.104	0.4653	-
SRI	10238	0.101	0.4614	-
EDN1	10526	0.096	0.4543	-
SCN1B	10662	0.094	0.4524	-
KCNJ2	10763	0.093	0.4517	-
RHOG	10947	0.09	0.448	-
LIF	10972	0.09	0.4498	-
ICAM4	11137	0.087	0.4467	-
TLR3	11343	0.084	0.4421	-
PIK3R5	11664	0.079	0.4333	-
RELA	11965	0.074	0.4251	-
ITGA5	12171	0.07	0.4201	-
IL10RA	12420	0.066	0.4134	-
SLC1A2	12438	0.066	0.4148	-
RNF144B	12691	0.062	0.4079	-
PTGER4	12862	0.06	0.4038	-
SLC31A2	13132	0.055	0.3961	-
PTGIR	13520	0.049	0.3841	-
OLR1	13821	0.044	0.375	-

PVR	14150	0.039	0.3647	-
RAF1	14279	0.037	0.3614	-
CCL7	14327	0.036	0.3608	-
TPBG	14339	0.036	0.3615	-
TNFRSF9	14651	0.031	0.3516	-
GPC3	14819	0.028	0.3466	-
IL18RAP	14874	0.027	0.3456	-
IRF1	15204	0.022	0.3348	-
INHBA	15360	0.02	0.33	-
P2RX7	15406	0.019	0.329	-
STAB1	15619	0.016	0.3221	-
PROK2	16153	0.009	0.3039	-
GPR132	21013	-0.016	0.1359	-
GABBR1	21280	-0.019	0.1273	-
MEFV	21324	-0.02	0.1264	-
VIP	21421	-0.021	0.1237	-
IL6	22003	-0.031	0.1044	-
SLC28A2	22018	-0.031	0.1049	-
CCL5	22138	-0.034	0.1018	-
NPFFR2	22188	-0.035	0.1011	-
CCL24	22363	-0.038	0.0962	-
CCL22	22502	-0.041	0.0926	-
P2RY2	22749	-0.047	0.0854	-
APLNR	22761	-0.047	0.0864	-
TNFSF10	22975	-0.052	0.0806	-
SCARF1	22981	-0.052	0.0819	-
FFAR2	23007	-0.052	0.0826	-
LTA	23220	-0.058	0.077	-
TACR3	23336	-0.061	0.0748	-
KCNA3	23351	-0.061	0.0761	-
BST2	23485	-0.064	0.0734	-
RASGRP1	23730	-0.07	0.067	-
SLC7A2	23843	-0.073	0.0653	-
MXD1	24048	-0.079	0.0605	-
ITGB3	24071	-0.079	0.0621	-
HAS2	24166	-0.082	0.0612	-
CCL20	24515	-0.092	0.0519	-
TNFAIP6	24530	-0.092	0.0541	-
CSF3R	24812	-0.1	0.0473	-
ADORA2B	24850	-0.102	0.049	-
P2RX4	25639	-0.126	0.0255	-

SLC4A4	25928	-0.137	0.0195	-
LDLR	26108	-0.145	0.0176	-
EBI3	26187	-0.147	0.0192	-
SELE	26874	-0.174	0.0006	-
CCL17	27195	-0.189	-0.0049	-
CD70	27321	-0.196	-0.0035	-
IL12B	27377	-0.199	0.0005	-
ADRM1	27630	-0.215	-0.0019	-
AHR	27640	-0.215	0.0041	-
KCNMB2	28199	-0.257	-0.0077	-
IL2RB	28285	-0.264	-0.0028	-
SLAMF1	28691	-0.316	-0.0075	-
OPRK1	28708	-0.318	0.0013	-
MEP1A	28842	-0.352	0.007	-

Table S14. Marker genes for single cell RNA-seq (scRNA-seq) analyses to cluster and annotate cell types in placenta and cord blood mononuclear cells (CBMCs).

**Placenta**	
**Cell type**	**Marker gene**
Villous cytotrophoblast cell (VCT)	*CDH1, MET, CCNB2, NRP2, PARP1, INSL4*
Syncytiotrophoblast cell (SCT)	*CYP19A1, CGA, ERVFRD-1, LGALS13, EGFR*
Extravillous trophoblast cell (EVT)	*HLA-G, PAPPA2, MMP2, TGFB1, CXCR6, MMP11*
Granulocyte	*FCGR3B, CXCL8, MNDA, SELL*
Myelocyte	*TCN1, CEACAM8, S100A8, MMP8, DEFA4, CAMP*
T cell	*CD3G, CD3D,*
Natural killer (NK) cell	*GNLY, NKG7*
B cell	*CD19, CD79A, CD79B, FCER2, MS4A1*
Monocyte	*CD14, CD300E, CD244, HLA-DRA, CLEC12A, FCN1*
Macrophage	*CD14, CD68, AIF1, CD163, CD209, CSFIR*
**Cord blood mononuclear cell (CBMC)**	
**Cell type**	**Marker gene**

B cell	*CD19, MS4A1*
Plasma cell (PC)	*CD19, MZB1, CD27*
CD4$^+$ T cell	*CD3D, CD4*
CD8$^+$ T cell	*CD3D, CD8A*
NK cell	*NKG7*
Granulocyte	*CEACAM3*
Monocyte	*CD14*
Platelet	*ITGA2B*
Plasmacytoid dendritic cell (pDC)	*IL2RA, IL3RA*
Hematopoietic stem cell (HSC)	*CD34*

Table S15. Gene set enrichment analysis details for HALLMARK_INFLAMMATORY_RESPONSE gene set comparing healthy Ctrl and GDM human umbilical vein endothelial cells (HUVECs).

Gene symbol	Rank in gene list	Rank metric score	Running enrichment score	Enrichment
TLR1	12	0.708	0.033	-
CCRL2	23	0.599	0.061	-
SEMA4D	182	0.35	0.0668	-
GPR132	226	0.326	0.0795	-
TNFSF15	234	0.322	0.0944	-
SLC7A1	289	0.3	0.1051	-
MARCO	316	0.291	0.1172	-
CD69	470	0.25	0.1187	-
ADGRE1	526	0.237	0.1262	-
GPR183	546	0.234	0.1361	-
TLR3	582	0.227	0.1446	-
CALCRL	954	0.178	0.1277	-
ACVR2A	955	0.178	0.1362	-
MSR1	1013	0.173	0.1406	-
GABBR1	1062	0.168	0.1453	-
NDP	1094	0.165	0.1511	-
NAMPT	1103	0.164	0.1584	-
P2RY2	1107	0.164	0.166	-
RGS1	1128	0.162	0.1724	-

RGS16	1271	0.153	0.17	-
TPBG	1415	0.145	0.1671	-
CCR7	1613	0.134	0.1601	-
CD40	1665	0.132	0.1629	-
LCK	1768	0.127	0.162	-
IL7R	2005	0.114	0.1513	-
PROK2	2048	0.112	0.1538	-
AQP9	2337	0.1	0.1388	-
TAPBP	2340	0.1	0.1435	-
SLC11A2	2414	0.098	0.1431	-
CMKLR1	2418	0.098	0.1476	-
BTG2	2518	0.094	0.1453	-
SPHK1	2574	0.092	0.146	-
LYN	2910	0.083	0.127	-
KCNMB2	2978	0.081	0.1262	-
PIK3R5	2982	0.081	0.1299	-
GCH1	3026	0.079	0.1307	-
BEST1	3037	0.079	0.1338	-
IRAK2	3049	0.079	0.1369	-
KCNJ2	3126	0.077	0.1353	-
OLR1	3216	0.075	0.1328	-
ATP2B1	3375	0.07	0.1253	-
APLNR	3378	0.07	0.1285	-
IRF1	3478	0.067	0.125	-
CD70	3629	0.064	0.1177	-
IL2RB	3663	0.063	0.1185	-
ADM	3695	0.062	0.1193	-
PDPN	3706	0.061	0.1215	-
GP1BA	3719	0.061	0.1236	-
C5AR1	3834	0.059	0.1186	-
SRI	3964	0.055	0.1124	-
ITGB3	3973	0.055	0.1145	-
SLC1A2	4081	0.053	0.1097	-
GPC3	4333	0.048	0.0948	-
PTGER2	4338	0.048	0.0968	-
CYBB	4456	0.045	0.0909	-
CD55	4519	0.044	0.0888	-
IL18	4652	0.042	0.0817	-
SCARF1	4713	0.041	0.0796	-
RHOG	4794	0.039	0.076	-
RNF144B	4837	0.039	0.0749	-

TNFRSF1B	4890	0.038	0.0732	-
ROS1	4918	0.037	0.0731	-
PTGER4	5054	0.035	0.0655	-
AXL	5217	0.032	0.056	-
CHST2	5232	0.032	0.0565	-
SGMS2	5322	0.03	0.0519	-
RAF1	5431	0.029	0.0459	-
ITGA5	5499	0.028	0.0426	-
MET	5517	0.027	0.0427	-
LAMP3	5525	0.027	0.0436	-
NMI	5587	0.026	0.0406	-
SLC4A4	5594	0.026	0.0415	-
SELL	5679	0.025	0.0369	-
HPN	5733	0.024	0.0344	-
PDE4B	5813	0.022	0.0301	-
CSF3R	5848	0.022	0.0288	-
NOD2	5938	0.02	0.0236	-
AHR	5987	0.019	0.0213	-
RELA	6075	0.018	0.0162	-
CCL5	6086	0.018	0.0164	-
PLAUR	6166	0.017	0.0118	-
SLAMF1	6389	0.014	-0.0028	-
LY6E	6809	0.008	-0.0311	-
CXCL9	6865	0.007	-0.0346	-
CLEC5A	7021	0.004	-0.045	-
CD82	7412	-0.001	-0.0717	-
STAB1	7585	-0.004	-0.0832	-
HIF1A	7602	-0.004	-0.0841	-
TNFRSF9	7632	-0.005	-0.0859	-
NFKB1	7641	-0.005	-0.0862	-
ADORA2B	7749	-0.007	-0.0932	-
MXD1	7865	-0.009	-0.1007	-
NLRP3	7968	-0.01	-0.1072	-
PTAFR	7996	-0.011	-0.1085	-
MYC	8050	-0.011	-0.1116	-
EIF2AK2	8121	-0.012	-0.1158	-
ATP2A2	8171	-0.013	-0.1185	-
PTPRE	8327	-0.016	-0.1284	-
EREG	8473	-0.018	-0.1375	-
RASGRP1	8549	-0.019	-0.1417	-
EDN1	8578	-0.019	-0.1427	-

ABI1	8595	-0.02	-0.1428	-
IL18RAP	8805	-0.023	-0.1561	-
GNAI3	8842	-0.024	-0.1574	-
HAS2	8910	-0.025	-0.1608	-
EMP3	8938	-0.026	-0.1614	-
IFITM1	9014	-0.027	-0.1653	-
ADRM1	9086	-0.028	-0.1688	-
KIF1B	9172	-0.029	-0.1732	-
KLF6	9243	-0.031	-0.1765	-
HRH1	9400	-0.034	-0.1856	-
SLC31A1	9681	-0.039	-0.2029	-
TIMP1	9842	-0.042	-0.2119	-
ITGB8	9852	-0.042	-0.2105	-
PSEN1	9967	-0.045	-0.2162	-
IRF7	10013	-0.045	-0.2171	-
FPR1	10380	-0.053	-0.2396	-
SLC31A2	10536	-0.056	-0.2475	-
TNFSF10	10673	-0.059	-0.254	-
F3	10688	-0.06	-0.2521	-
IL1B	10804	-0.063	-0.257	-
ACVR1B	10963	-0.066	-0.2646	-
NFKBIA	11241	-0.074	-0.2801	-
IL15RA	11312	-0.076	-0.2812	-
IFNGR2	11349	-0.077	-0.28	-
SERPINE1	11444	-0.079	-0.2827	-
CX3CL1	11777	-0.09	-0.3011	-
CDKN1A	11864	-0.094	-0.3025	-
GNA15	12066	-0.1	-0.3115	-
IL6	12105	-0.101	-0.3093	-
CXCL8	12306	-0.108	-0.3178	↑ GDM
OSM	12346	-0.109	-0.3152	↑ GDM
ATP2C1	12439	-0.112	-0.3162	↑ GDM
FZD5	12484	-0.114	-0.3137	↑ GDM
CSF1	12486	-0.114	-0.3083	↑ GDM
CXCR6	12602	-0.119	-0.3105	↑ GDM
CD48	12654	-0.121	-0.3082	↑ GDM
CXCL10	12712	-0.125	-0.3062	↑ GDM
MMP14	12904	-0.134	-0.3129	↑ GDM
BST2	13001	-0.14	-0.3128	↑ GDM
LCP2	13010	-0.14	-0.3066	↑ GDM
C3AR1	13064	-0.143	-0.3034	↑ GDM

IL12B	13136	-0.148	-0.3012	↑ GDM
IL4R	13149	-0.149	-0.2949	↑ GDM
PVR	13328	-0.16	-0.2995	↑ GDM
IFNAR1	13561	-0.179	-0.3068	↑ GDM
HBEGF	13759	-0.199	-0.3108	↑ GDM
P2RX4	13831	-0.208	-0.3057	↑ GDM
LDLR	13841	-0.209	-0.2964	↑ GDM
LIF	13862	-0.211	-0.2876	↑ GDM
DCBLD2	13908	-0.218	-0.2803	↑ GDM
TLR2	13938	-0.222	-0.2717	↑ GDM
TNFSF9	13987	-0.227	-0.2641	↑ GDM
LPAR1	14001	-0.229	-0.254	↑ GDM
CCL2	14039	-0.234	-0.2454	↑ GDM
CD14	14067	-0.238	-0.2358	↑ GDM
ICAM4	14117	-0.247	-0.2274	↑ GDM
IL10RA	14145	-0.253	-0.2171	↑ GDM
P2RX7	14197	-0.264	-0.208	↑ GDM
FFAR2	14213	-0.268	-0.1962	↑ GDM
PCDH7	14277	-0.285	-0.1869	↑ GDM
IL18R1	14298	-0.292	-0.1743	↑ GDM
SELE	14342	-0.303	-0.1628	↑ GDM
IL1R1	14414	-0.331	-0.1518	↑ GDM
RIPK2	14457	-0.349	-0.138	↑ GDM
INHBA	14463	-0.352	-0.1215	↑ GDM
OSMR	14513	-0.376	-0.1069	↑ GDM
CXCL6	14521	-0.381	-0.0892	↑ GDM
ABCA1	14569	-0.415	-0.0725	↑ GDM
TNFAIP6	14669	-0.544	-0.0533	↑ GDM
IL15	14672	-0.55	-0.0271	↑ GDM
ICAM1	14721	-0.704	0.0032	↑ GDM

Table S16. Gene set enrichment analysis details for HALLMARK_TNFA_SIGNALING_VIA_NFKB gene set comparing healthy Ctrl and GDM human umbilical vein endothelial cells (HUVECs).

Gene symbol	Rank in gene list	Rank metric score	Running enrichment score	Enrichment
CCRL2	23	0.599	0.0233	-
SLC2A3	169	0.357	0.0281	-
CFLAR	186	0.348	0.0414	-
B4GALT1	211	0.335	0.0537	-
KDM6B	251	0.313	0.064	-
IRS2	447	0.256	0.0612	-
CD69	470	0.25	0.0701	-

GPR183	546	0.234	0.0747	-
NFAT5	701	0.207	0.0727	-
SERPINB2	848	0.189	0.0705	-
DUSP4	960	0.178	0.0703	-
DUSP5	990	0.175	0.0755	-
NAMPT	1103	0.164	0.0747	-
SIK1	1250	0.155	0.0711	-
BTG1	1300	0.152	0.074	-
RNF19B	1355	0.149	0.0764	-
SOD2	1567	0.137	0.0677	-
KYNU	1589	0.136	0.0718	-
STAT5A	1637	0.133	0.0741	-
CD80	1742	0.128	0.0723	-
IL7R	2005	0.114	0.0591	-
PLPP3	2061	0.112	0.0599	-
FOSL1	2335	0.1	0.0454	-
TGIF1	2411	0.098	0.0443	-
TRIB1	2494	0.095	0.0426	-
BTG2	2518	0.094	0.0449	-
SPHK1	2574	0.092	0.045	-
RIGI	2606	0.091	0.0466	-
ACKR3	2638	0.09	0.0482	-
PER1	2662	0.089	0.0503	-
PLEK	2675	0.089	0.0532	-
SLC16A6	2691	0.089	0.0559	-
FUT4	2900	0.083	0.045	-
IL23A	2986	0.081	0.0425	-
GCH1	3026	0.079	0.0432	-
OLR1	3216	0.075	0.0333	-
ATP2B1	3375	0.07	0.0253	-
TAP1	3449	0.068	0.0231	-
IRF1	3478	0.067	0.024	-
B4GALT5	4009	0.054	-0.0101	-
TRIP10	4124	0.052	-0.0158	-
BCL2A1	4400	0.046	-0.0327	-
SERPINB8	4437	0.046	-0.0333	-
ID2	4588	0.043	-0.0418	-
IL18	4652	0.042	-0.0444	-
YRDC	4668	0.041	-0.0437	-
DUSP1	4676	0.041	-0.0425	-
SNN	4877	0.038	-0.0546	-

BMP2	4968	0.036	-0.0593	-
ZFP36	4995	0.036	-0.0596	-
PTGER4	5054	0.035	-0.0621	-
EGR1	5342	0.03	-0.0806	-
PNRC1	5370	0.03	-0.0812	-
F2RL1	5401	0.029	-0.082	-
EHD1	5488	0.028	-0.0868	-
PDE4B	5813	0.022	-0.1081	-
RELA	6075	0.018	-0.1252	-
CCL5	6086	0.018	-0.1252	-
PLAUR	6166	0.017	-0.1299	-
DNAJB4	6199	0.016	-0.1314	-
MARCKS	6243	0.016	-0.1337	-
EIF1	6287	0.015	-0.136	-
TNF	6343	0.014	-0.1392	-
SPSB1	6558	0.011	-0.1534	-
RHOB	6568	0.011	-0.1536	-
IER2	6772	0.008	-0.1672	-
NR4A1	7368	-0.001	-0.2079	-
KLF10	7593	-0.004	-0.2231	-
TNFRSF9	7632	-0.005	-0.2255	-
NFKB1	7641	-0.005	-0.2259	-
KLF4	7771	-0.007	-0.2344	-
IL6ST	7821	-0.008	-0.2375	-
MXD1	7865	-0.009	-0.2401	-
TUBB2A	7946	-0.01	-0.2451	-
CLCF1	7988	-0.01	-0.2475	-
MYC	8050	-0.011	-0.2512	-
PDLIM5	8081	-0.012	-0.2528	-
HES1	8165	-0.013	-0.258	-
BIRC2	8246	-0.014	-0.2628	-
PTPRE	8327	-0.016	-0.2677	-
CCL4	8397	-0.017	-0.2717	-
TSC22D1	8427	-0.017	-0.273	-
BTG3	8468	-0.018	-0.275	-
EDN1	8578	-0.019	-0.2817	-
TNFAIP2	8620	-0.02	-0.2837	-
DRAM1	8681	-0.021	-0.2869	-
NFKBIE	8779	-0.023	-0.2926	-
SDC4	9163	-0.029	-0.3177	-
KLF6	9243	-0.031	-0.3218	-

TANK	9291	-0.032	-0.3237	-
REL	9325	-0.032	-0.3247	-
CCND1	9452	-0.035	-0.3319	-
JUN	9455	-0.035	-0.3306	-
SGK1	9458	-0.035	-0.3293	-
LITAF	9479	-0.035	-0.3292	-
FOS	9568	-0.037	-0.3337	-
CCN1	9587	-0.037	-0.3334	-
PTX3	9711	-0.04	-0.3402	-
AREG	9862	-0.043	-0.3487	-
SMAD3	9885	-0.043	-0.3484	-
CEBPB	10053	-0.046	-0.358	-
MAFF	10067	-0.047	-0.3569	-
MAP2K3	10072	-0.047	-0.3553	-
TNIP2	10205	-0.05	-0.3622	-
IER5	10384	-0.053	-0.3722	-
MCL1	10393	-0.053	-0.3706	-
SQSTM1	10527	-0.056	-0.3774	-
NFE2L2	10571	-0.057	-0.3779	-
F3	10688	-0.06	-0.3834	-
JUNB	10732	-0.061	-0.3838	-
IL1B	10804	-0.063	-0.3861	-
EFNA1	10946	-0.066	-0.393	-
KLF2	11085	-0.069	-0.3996	-
PTGS2	11225	-0.073	-0.4061	-
NFKBIA	11241	-0.074	-0.4041	-
IL15RA	11312	-0.076	-0.4058	-
SOCS3	11336	-0.077	-0.4042	-
PLK2	11347	-0.077	-0.4017	-
IFNGR2	11349	-0.077	-0.3985	-
PPP1R15A	11361	-0.077	-0.3961	-
SERPINE1	11444	-0.079	-0.3984	-
TRAF1	11636	-0.085	-0.408	-
PHLDA2	11709	-0.088	-0.4093	-
NFKB2	11731	-0.088	-0.4071	-
IER3	11773	-0.09	-0.4062	-
BIRC3	11804	-0.091	-0.4044	-
CDKN1A	11864	-0.094	-0.4046	-
BCL6	11891	-0.094	-0.4025	-
DENND5A	12092	-0.101	-0.412	-
IL6	12105	-0.101	-0.4086	-

ZC3H12A	12208	-0.105	-0.4113	-
BCL3	12270	-0.107	-0.411	-
ZBTB10	12356	-0.11	-0.4123	-
ETS2	12441	-0.113	-0.4134	-
CSF1	12486	-0.114	-0.4117	-
SAT1	12711	-0.125	-0.4219	↑ GDM
CXCL10	12712	-0.125	-0.4167	↑ GDM
MSC	12716	-0.125	-0.4118	↑ GDM
NFIL3	12742	-0.126	-0.4083	↑ GDM
TNFAIP8	12796	-0.128	-0.4066	↑ GDM
JAG1	12828	-0.13	-0.4033	↑ GDM
TIPARP	12864	-0.132	-0.4003	↑ GDM
TNIP1	12901	-0.134	-0.3972	↑ GDM
TNC	12926	-0.135	-0.3932	↑ GDM
RCAN1	12934	-0.136	-0.3881	↑ GDM
VEGFA	13124	-0.147	-0.3949	↑ GDM
IL12B	13136	-0.148	-0.3896	↑ GDM
CCNL1	13370	-0.162	-0.3988	↑ GDM
CD44	13384	-0.164	-0.3929	↑ GDM
IFIH1	13387	-0.164	-0.3863	↑ GDM
ATF3	13407	-0.166	-0.3807	↑ GDM
GADD45A	13549	-0.178	-0.383	↑ GDM
BHLHE40	13584	-0.181	-0.3778	↑ GDM
PFKFB3	13624	-0.184	-0.3729	↑ GDM
PMEPA1	13698	-0.192	-0.3699	↑ GDM
RELB	13739	-0.196	-0.3645	↑ GDM
HBEGF	13759	-0.199	-0.3576	↑ GDM
LDLR	13841	-0.209	-0.3545	↑ GDM
TNFAIP3	13851	-0.21	-0.3464	↑ GDM
LIF	13862	-0.211	-0.3383	↑ GDM
CXCL3	13895	-0.216	-0.3315	↑ GDM
LAMB3	13897	-0.216	-0.3226	↑ GDM
NINJ1	13902	-0.216	-0.3139	↑ GDM
KLF9	13909	-0.218	-0.3053	↑ GDM
DUSP2	13912	-0.218	-0.2964	↑ GDM
PLAU	13932	-0.221	-0.2885	↑ GDM
TLR2	13938	-0.222	-0.2797	↑ GDM
IFIT2	13941	-0.222	-0.2706	↑ GDM
TNFSF9	13987	-0.227	-0.2643	↑ GDM
GADD45B	13993	-0.229	-0.2552	↑ GDM
CCL2	14039	-0.234	-0.2485	↑ GDM

CD83	14073	-0.239	-0.2409	↑ GDM
NR4A2	14256	-0.278	-0.2419	↑ GDM
FOSB	14261	-0.279	-0.2306	↑ GDM
FOSL2	14391	-0.322	-0.2261	↑ GDM
RIPK2	14457	-0.349	-0.216	↑ GDM
GEM	14458	-0.35	-0.2016	↑ GDM
INHBA	14463	-0.352	-0.1873	↑ GDM
CXCL2	14469	-0.354	-0.1729	↑ GDM
NR4A3	14483	-0.361	-0.1589	↑ GDM
CXCL6	14521	-0.381	-0.1456	↑ GDM
EGR3	14525	-0.384	-0.1299	↑ GDM
CXCL1	14558	-0.404	-0.1153	↑ GDM
ABCA1	14569	-0.415	-0.0988	↑ GDM
GFPT2	14599	-0.442	-0.0825	↑ GDM
MAP3K8	14615	-0.464	-0.0642	↑ GDM
TNFAIP6	14669	-0.544	-0.0453	↑ GDM
G0S2	14671	-0.55	-0.0226	↑ GDM
ICAM1	14721	-0.704	0.0032	↑ GDM

Table S17. Gene set enrichment analysis details for HALLMARK_INFLAMMATORY_RESPONSE gene set comparing healthy Ctrl and GDM amniocytes.

Gene symbol	Rank in gene list	Rank metric score	Running enrichment score	Enrichment
CYBB	655	0.479	-0.0204	-
IL18	1027	0.433	-0.0287	-
CALCRL	1440	0.393	-0.0396	-
PTAFR	1761	0.367	-0.0469	-
SRI	1776	0.366	-0.04	-
SCN1B	1844	0.36	-0.0356	-
APLNR	1866	0.359	-0.0292	-
LPAR1	1974	0.352	-0.0268	-
AHR	2471	0.324	-0.0431	-
LCK	2561	0.319	-0.0406	-
AXL	2633	0.315	-0.0374	-
SLC4A4	2742	0.309	-0.036	-
SEMA4D	2842	0.304	-0.0343	-
GABBR1	2908	0.301	-0.0311	-
PTGER2	2983	0.298	-0.0284	-
PTGER4	3219	0.288	-0.0333	-
CX3CL1	4284	0.248	-0.0775	-
GNA15	4404	0.244	-0.078	-
NDP	4828	0.231	-0.0928	-

GPC3	5009	0.224	-0.0965	-
PCDH7	5101	0.221	-0.0961	-
HBEGF	5210	0.217	-0.0966	-
MYC	5383	0.212	-0.1002	-
SELL	5462	0.209	-0.0995	-
SLC28A2	5923	0.195	-0.1168	-
HPN	6167	0.189	-0.1241	-
IFNGR2	6174	0.189	-0.1205	-
MET	6630	0.175	-0.138	-
C5AR1	6850	0.169	-0.1446	-
IL1R1	6883	0.168	-0.1426	-
HRH1	7011	0.164	-0.1451	-
GNAI3	7050	0.163	-0.1435	-
DCBLD2	7062	0.162	-0.1407	-
ADORA2B	7150	0.16	-0.1414	-
CHST2	7172	0.159	-0.1391	-
RAF1	7612	0.147	-0.1564	-
ACVR1B	7839	0.142	-0.1639	-
TNFSF10	7859	0.141	-0.1619	-
TNFRSF1B	8164	0.134	-0.1732	-
KIF1B	8313	0.13	-0.1774	-
HIF1A	8952	0.112	-0.2046	-
GP1BA	9397	0.101	-0.2231	-
SGMS2	9532	0.098	-0.2273	-
CD70	9662	0.094	-0.2313	-
EBI3	9694	0.093	-0.2308	-
OSMR	10352	0.074	-0.2598	-
MSR1	10364	0.074	-0.2587	-
TACR1	10558	0.068	-0.2663	-
GPR132	10754	0.062	-0.274	-
PTPRE	10836	0.059	-0.2766	-
ICOSLG	11092	0.052	-0.2873	-
PVR	11161	0.049	-0.2894	-
GPR183	11209	0.048	-0.2906	-
CCR7	11430	0.042	-0.3	-
INHBA	11663	0.034	-0.31	-
IL4R	11713	0.033	-0.3116	-
IFNAR1	11737	0.032	-0.312	-
F3	11993	0.024	-0.3233	-
MMP14	12285	0.015	-0.3365	-
ATP2A2	12437	0.011	-0.3432	-

IL7R	12441	0.011	-0.3432	-
IRAK2	12516	0.008	-0.3464	-
SLC7A1	12674	0.003	-0.3536	-
ADRM1	12723	0.001	-0.3558	-
IL10	12817	-0.002	-0.3601	-
CD40	13352	-0.019	-0.3845	-
TAPBP	13471	-0.022	-0.3895	-
BST2	13750	-0.031	-0.4017	-
ITGB8	13776	-0.033	-0.4022	-
NPFFR2	13910	-0.037	-0.4076	-
CXCR6	13912	-0.037	-0.4069	-
FZD5	13997	-0.04	-0.4099	-
ITGB3	14053	-0.042	-0.4116	-
OSM	14320	-0.051	-0.4229	-
CXCL6	14323	-0.051	-0.4219	-
CD69	14352	-0.052	-0.4221	-
CCL20	14608	-0.062	-0.4327	-
SCARF1	14857	-0.072	-0.4427	-
ACVR2A	14871	-0.073	-0.4418	-
RIPK2	14941	-0.076	-0.4434	-
EDN1	15440	-0.095	-0.4645	-
RNF144B	15458	-0.096	-0.4633	-
P2RX4	15742	-0.107	-0.4742	-
RASGRP1	15812	-0.11	-0.4751	-
RHOG	16066	-0.121	-0.4844	-
TNFAIP6	16140	-0.123	-0.4852	-
EREG	16241	-0.128	-0.4872	-
ICAM4	16258	-0.129	-0.4852	-
CD14	16280	-0.13	-0.4835	-
ATP2C1	16343	-0.133	-0.4836	-
PSEN1	16353	-0.134	-0.4813	-
PLAUR	16473	-0.14	-0.4839	-
AQP9	16523	-0.143	-0.4832	-
LDLR	16562	-0.145	-0.482	-
PDE4B	16761	-0.155	-0.4879	-
IRF1	16805	-0.157	-0.4867	-
ABI1	16870	-0.159	-0.4864	-
CD55	16919	-0.162	-0.4852	-
TNFRSF9	17015	-0.166	-0.4862	-
CXCL8	17168	-0.173	-0.4897	↑ GDM
CD82	17247	-0.176	-0.4896	↑ GDM

CSF1	17252	-0.177	-0.4862	↑ GDM
NFKBIA	17290	-0.179	-0.4842	↑ GDM
HAS2	17315	-0.18	-0.4816	↑ GDM
ADM	17459	-0.187	-0.4843	↑ GDM
KLF6	17636	-0.197	-0.4884	↑ GDM
RELA	17715	-0.202	-0.4879	↑ GDM
NOD2	17750	-0.204	-0.4852	↑ GDM
IL15	17786	-0.205	-0.4826	↑ GDM
SLC31A1	17788	-0.206	-0.4784	↑ GDM
NLRP3	17837	-0.208	-0.4763	↑ GDM
ATP2B1	17892	-0.211	-0.4744	↑ GDM
KCNJ2	18195	-0.231	-0.4836	↑ GDM
BDKRB1	18223	-0.232	-0.4801	↑ GDM
CCL7	18249	-0.234	-0.4764	↑ GDM
RTP4	18260	-0.235	-0.472	↑ GDM
PDPN	18376	-0.242	-0.4723	↑ GDM
CLEC5A	18461	-0.247	-0.4711	↑ GDM
RGS16	18565	-0.254	-0.4706	↑ GDM
P2RY2	18612	-0.257	-0.4674	↑ GDM
NAMPT	18626	-0.258	-0.4627	↑ GDM
CXCL10	18637	-0.259	-0.4578	↑ GDM
LCP2	18666	-0.261	-0.4537	↑ GDM
FPR1	18702	-0.263	-0.4499	↑ GDM
EMP3	18707	-0.264	-0.4446	↑ GDM
STAB1	18967	-0.283	-0.4508	↑ GDM
SELENOS	19019	-0.287	-0.4472	↑ GDM
BTG2	19024	-0.287	-0.4415	↑ GDM
RGS1	19111	-0.294	-0.4394	↑ GDM
SLC7A2	19138	-0.296	-0.4345	↑ GDM
LIF	19274	-0.306	-0.4344	↑ GDM
IL12B	19354	-0.313	-0.4316	↑ GDM
MEFV	19383	-0.315	-0.4263	↑ GDM
PTGIR	19520	-0.327	-0.4259	↑ GDM
LTA	19590	-0.334	-0.4222	↑ GDM
GCH1	19595	-0.334	-0.4155	↑ GDM
PIK3R5	19634	-0.338	-0.4102	↑ GDM
LYN	19651	-0.339	-0.404	↑ GDM
NMI	19685	-0.342	-0.3984	↑ GDM
CSF3R	19784	-0.352	-0.3957	↑ GDM
SERPINE1	19829	-0.357	-0.3903	↑ GDM
LY6E	19863	-0.359	-0.3844	↑ GDM

TNFSF9	19886	-0.362	-0.378	↑ GDM
IL2RB	19961	-0.37	-0.3737	↑ GDM
IL15RA	19971	-0.371	-0.3665	↑ GDM
TIMP1	20054	-0.378	-0.3625	↑ GDM
CSF3	20114	-0.384	-0.3573	↑ GDM
C3AR1	20137	-0.386	-0.3503	↑ GDM
VIP	20169	-0.389	-0.3437	↑ GDM
ABCA1	20197	-0.392	-0.3368	↑ GDM
IL10RA	20212	-0.394	-0.3293	↑ GDM
CCRL2	20275	-0.403	-0.3239	↑ GDM
ITGA5	20282	-0.403	-0.3158	↑ GDM
TPBG	20293	-0.405	-0.3079	↑ GDM
ICAM1	20363	-0.414	-0.3025	↑ GDM
TACR3	20390	-0.416	-0.2951	↑ GDM
IL6	20395	-0.417	-0.2867	↑ GDM
TLR1	20405	-0.418	-0.2785	↑ GDM
MARCO	20412	-0.419	-0.2701	↑ GDM
IL18R1	20471	-0.426	-0.264	↑ GDM
NFKB1	20480	-0.426	-0.2555	↑ GDM
KCNMB2	20494	-0.429	-0.2473	↑ GDM
CDKN1A	20507	-0.43	-0.2389	↑ GDM
CMKLR1	20657	-0.45	-0.2365	↑ GDM
P2RX7	20660	-0.451	-0.2273	↑ GDM
IL1B	20939	-0.501	-0.2298	↑ GDM
CXCL11	20976	-0.511	-0.2209	↑ GDM
IFITM1	21018	-0.521	-0.212	↑ GDM
SLC11A2	21028	-0.523	-0.2016	↑ GDM
ROS1	21123	-0.549	-0.1946	↑ GDM
TLR2	21134	-0.552	-0.1837	↑ GDM
IL1A	21160	-0.559	-0.1733	↑ GDM
BEST1	21246	-0.587	-0.1651	↑ GDM
IL18RAP	21249	-0.588	-0.153	↑ GDM
CCL2	21252	-0.589	-0.1409	↑ GDM
EIF2AK2	21280	-0.599	-0.1298	↑ GDM
IRF7	21299	-0.607	-0.1181	↑ GDM
MXD1	21324	-0.615	-0.1065	↑ GDM
TLR3	21391	-0.639	-0.0963	↑ GDM
CCL5	21429	-0.659	-0.0844	↑ GDM
SPHK1	21441	-0.666	-0.0711	↑ GDM
TNFSF15	21454	-0.671	-0.0578	↑ GDM
LAMP3	21648	-0.858	-0.049	↑ GDM

SLC1A2	21650	-0.86	-0.0312	↑ GDM
OLR1	21661	-0.877	-0.0136	↑ GDM
SLC31A2	21664	-0.88	0.0045	↑ GDM

Table S18. Gene set enrichment analysis details for HALLMARK_TNFA_SIGNALING_VIA_NFKB gene set comparing healthy Ctrl and GDM amniocytes.

Gene symbol	Rank in gene list	Rank metric score	Running enrichment score	Enrichment
DUSP4	755	0.465	-0.0249	-
IL18	1027	0.433	-0.028	-
SPSB1	1215	0.413	-0.0276	-
MARCKS	1355	0.4	-0.0253	-
BCL3	1450	0.392	-0.0211	-
TNFAIP8	1573	0.382	-0.0184	-
DUSP2	1775	0.366	-0.0198	-
PFKFB3	2515	0.321	-0.047	-
PLK2	2662	0.314	-0.047	-
PTGER4	3219	0.288	-0.0665	-
GADD45B	3683	0.269	-0.082	-
PHLDA2	3998	0.258	-0.091	-
ID2	4068	0.255	-0.0886	-
B4GALT5	4079	0.255	-0.0835	-
BMP2	4493	0.242	-0.0974	-
JAG1	4505	0.241	-0.0926	-
RHOB	4912	0.228	-0.1065	-
SOCS3	5099	0.221	-0.1103	-
HBEGF	5210	0.217	-0.1106	-
MYC	5383	0.212	-0.114	-
IER2	5747	0.2	-0.1265	-
SNN	5759	0.2	-0.1226	-
JUNB	5793	0.199	-0.1198	-
PER1	6030	0.193	-0.1265	-
IFNGR2	6174	0.189	-0.129	-
FJX1	6612	0.176	-0.1454	-
ZBTB10	6663	0.174	-0.144	-
KLF2	6799	0.171	-0.1465	-
JUN	6991	0.165	-0.1518	-
TUBB2A	7004	0.164	-0.1487	-
CEBPD	7346	0.155	-0.1612	-
KDM6B	7403	0.153	-0.1604	-
CLCF1	7674	0.146	-0.1697	-
TRIP10	7779	0.143	-0.1714	-

SGK1	7845	0.142	-0.1714	-
F2RL1	8059	0.136	-0.1783	-
PDLIM5	8534	0.124	-0.1975	-
IER3	8558	0.123	-0.1959	-
FOSB	8707	0.119	-0.2002	-
FOS	8890	0.115	-0.2061	-
TSC22D1	9165	0.107	-0.2165	-
SLC16A6	9653	0.094	-0.237	-
EGR1	9666	0.094	-0.2355	-
MCL1	9958	0.085	-0.2471	-
BTG3	9994	0.084	-0.2469	-
ZFP36	10011	0.084	-0.2458	-
B4GALT1	10162	0.08	-0.251	-
TNFAIP2	10198	0.078	-0.251	-
PMEPA1	10234	0.077	-0.2509	-
YRDC	10355	0.074	-0.2548	-
CCN1	10514	0.069	-0.2607	-
FOSL2	10539	0.068	-0.2603	-
KLF4	10699	0.064	-0.2663	-
EFNA1	10769	0.061	-0.2681	-
PTPRE	10836	0.059	-0.2699	-
EHD1	10892	0.058	-0.2712	-
FOSL1	10967	0.055	-0.2734	-
ICOSLG	11092	0.052	-0.278	-
SDC4	11098	0.051	-0.2771	-
GPR183	11209	0.048	-0.2812	-
TIPARP	11360	0.044	-0.2872	-
INHBA	11663	0.034	-0.3005	-
SLC2A3	11974	0.025	-0.3143	-
F3	11993	0.024	-0.3146	-
CCL4	12004	0.024	-0.3145	-
NR4A1	12226	0.017	-0.3244	-
IL7R	12441	0.011	-0.3341	-
HES1	12490	0.009	-0.3361	-
MAP2K3	12770	0	-0.3491	-
PLAU	12893	-0.004	-0.3546	-
LAMB3	12901	-0.005	-0.3548	-
PHLDA1	13369	-0.019	-0.3761	-
TNIP2	13566	-0.025	-0.3846	-
TNF	13873	-0.036	-0.398	-
TNC	14010	-0.041	-0.4034	-

BHLHE40	14036	-0.041	-0.4037	-
TRAF1	14083	-0.043	-0.4049	-
PANX1	14093	-0.043	-0.4044	-
KLF10	14130	-0.044	-0.4051	-
STAT5A	14153	-0.045	-0.4051	-
G0S2	14157	-0.045	-0.4043	-
BIRC2	14171	-0.045	-0.4039	-
CXCL6	14323	-0.051	-0.4098	-
CFLAR	14331	-0.051	-0.409	-
CD69	14352	-0.052	-0.4088	-
RCAN1	14421	-0.055	-0.4107	-
CCL20	14608	-0.062	-0.418	-
SMAD3	14637	-0.064	-0.4179	-
NFAT5	14817	-0.071	-0.4247	-
RIPK2	14941	-0.076	-0.4287	-
BIRC3	14944	-0.076	-0.4271	-
RELB	15004	-0.078	-0.4282	-
IL23A	15031	-0.079	-0.4277	-
CD80	15054	-0.079	-0.4269	-
CD44	15059	-0.08	-0.4254	-
CCND1	15299	-0.089	-0.4345	-
PNRC1	15304	-0.089	-0.4328	-
EDN1	15440	-0.095	-0.437	-
NR4A3	15499	-0.097	-0.4375	-
KLF9	15614	-0.102	-0.4406	-
TRIB1	15622	-0.103	-0.4387	-
SLC2A6	15664	-0.104	-0.4383	-
CSF2	15795	-0.109	-0.4419	-
DUSP1	15952	-0.116	-0.4466	-
DNAJB4	15958	-0.116	-0.4443	-
DUSP5	16061	-0.12	-0.4464	-
BCL2A1	16125	-0.123	-0.4467	-
TNFAIP6	16140	-0.123	-0.4446	-
ATF3	16272	-0.13	-0.4479	-
TNIP1	16333	-0.133	-0.4477	-
TANK	16419	-0.138	-0.4487	-
PLAUR	16473	-0.14	-0.4481	-
LDLR	16562	-0.145	-0.449	↑ GDM
BTG1	16580	-0.146	-0.4466	↑ GDM
BCL6	16590	-0.146	-0.4438	↑ GDM
CXCL2	16606	-0.147	-0.4413	↑ GDM

PDE4B	16761	-0.155	-0.4451	↑ GDM
REL	16764	-0.155	-0.4418	↑ GDM
EGR3	16800	-0.156	-0.44	↑ GDM
IRF1	16805	-0.157	-0.4367	↑ GDM
EIF1	16872	-0.16	-0.4363	↑ GDM
SAT1	16915	-0.162	-0.4347	↑ GDM
ZC3H12A	16922	-0.162	-0.4315	↑ GDM
TNFRSF9	17015	-0.166	-0.4321	↑ GDM
CXCL3	17196	-0.174	-0.4367	↑ GDM
NFKB2	17249	-0.177	-0.4352	↑ GDM
CSF1	17252	-0.177	-0.4314	↑ GDM
SERPINB2	17270	-0.178	-0.4284	↑ GDM
NFKBIA	17290	-0.179	-0.4253	↑ GDM
FUT4	17366	-0.182	-0.4248	↑ GDM
NFKBIE	17445	-0.187	-0.4244	↑ GDM
CXCL1	17603	-0.196	-0.4274	↑ GDM
KLF6	17636	-0.197	-0.4246	↑ GDM
RELA	17715	-0.202	-0.4238	↑ GDM
ATP2B1	17892	-0.211	-0.4273	↑ GDM
MAFF	17913	-0.212	-0.4236	↑ GDM
NINJ1	17916	-0.213	-0.4191	↑ GDM
CCNL1	18010	-0.219	-0.4186	↑ GDM
NFE2L2	18046	-0.222	-0.4154	↑ GDM
NFIL3	18047	-0.222	-0.4105	↑ GDM
LITAF	18193	-0.231	-0.4122	↑ GDM
MAP3K8	18265	-0.235	-0.4104	↑ GDM
TGIF1	18304	-0.238	-0.4069	↑ GDM
NR4A2	18436	-0.245	-0.4076	↑ GDM
NAMPT	18626	-0.258	-0.4108	↑ GDM
PLPP3	18627	-0.258	-0.4052	↑ GDM
TNFAIP3	18632	-0.258	-0.3997	↑ GDM
CXCL10	18637	-0.259	-0.3942	↑ GDM
EGR2	18655	-0.26	-0.3894	↑ GDM
SOD2	18760	-0.267	-0.3884	↑ GDM
VEGFA	18780	-0.269	-0.3834	↑ GDM
IER5	18980	-0.284	-0.3864	↑ GDM
ETS2	18983	-0.284	-0.3803	↑ GDM
BTG2	19024	-0.287	-0.3759	↑ GDM
PLEK	19104	-0.294	-0.3731	↑ GDM
LIF	19274	-0.306	-0.3743	↑ GDM
IL12B	19354	-0.313	-0.3711	↑ GDM

GFPT2	19565	-0.331	-0.3736	↑ GDM
DRAM1	19584	-0.333	-0.3672	↑ GDM
GCH1	19595	-0.334	-0.3603	↑ GDM
KYNU	19637	-0.338	-0.3549	↑ GDM
PPP1R15A	19768	-0.351	-0.3532	↑ GDM
SERPINE1	19829	-0.357	-0.3482	↑ GDM
RNF19B	19836	-0.357	-0.3407	↑ GDM
CD83	19874	-0.361	-0.3346	↑ GDM
TNFSF9	19886	-0.362	-0.3272	↑ GDM
IL15RA	19971	-0.371	-0.323	↑ GDM
IL6ST	20065	-0.379	-0.319	↑ GDM
DENND5A	20075	-0.38	-0.3111	↑ GDM
GEM	20132	-0.385	-0.3053	↑ GDM
ABCA1	20197	-0.392	-0.2997	↑ GDM
CCRL2	20275	-0.403	-0.2945	↑ GDM
ICAM1	20363	-0.414	-0.2895	↑ GDM
IL6	20395	-0.417	-0.2818	↑ GDM
IRS2	20478	-0.426	-0.2763	↑ GDM
NFKB1	20480	-0.426	-0.2671	↑ GDM
CDKN1A	20507	-0.43	-0.2589	↑ GDM
GADD45A	20612	-0.444	-0.254	↑ GDM
IL1B	20939	-0.501	-0.2582	↑ GDM
PTX3	20949	-0.503	-0.2476	↑ GDM
ACKR3	20973	-0.51	-0.2375	↑ GDM
CXCL11	20976	-0.511	-0.2265	↑ GDM
CEBPB	21024	-0.522	-0.2172	↑ GDM
TLR2	21134	-0.552	-0.2103	↑ GDM
IL1A	21160	-0.559	-0.1992	↑ GDM
SERPINB8	21172	-0.563	-0.1874	↑ GDM
CCL2	21252	-0.589	-0.1782	↑ GDM
TAP1	21281	-0.599	-0.1664	↑ GDM
MXD1	21324	-0.615	-0.1549	↑ GDM
AREG	21408	-0.649	-0.1446	↑ GDM
CCL5	21429	-0.659	-0.1312	↑ GDM
SPHK1	21441	-0.666	-0.1171	↑ GDM
PTGS2	21531	-0.722	-0.1055	↑ GDM
MSC	21586	-0.774	-0.0911	↑ GDM
RIGI	21644	-0.854	-0.0751	↑ GDM
IFIH1	21656	-0.867	-0.0567	↑ GDM
OLR1	21661	-0.877	-0.0377	↑ GDM
IFIT2	21672	-0.895	-0.0186	↑ GDM

SQSTM1	21730	-1.041	0.0015	↑ GDM

Table S19. Gene set enrichment analysis details for HALLMARK_INFLAMMATORY_RESPONSE gene set comparing healthy Ctrl and GDM cord blood mononuclear cells (CBMCs).

Gene symbol	Rank in gene list	Rank metric score	Running enrichment score	Enrichment
CCL22	234	0.303	0.0038	-
ICOSLG	404	0.264	0.0083	-
NDP	885	0.203	-0.0003	-
SLC7A1	1240	0.175	-0.0057	-
RGS16	1467	0.162	-0.0072	-
LPAR1	1548	0.157	-0.0038	-
SLC1A2	1790	0.146	-0.0064	-
IL2RB	2134	0.131	-0.0132	-
KCNMB2	2232	0.128	-0.0115	-
SLC11A2	2459	0.12	-0.0146	-
SLC28A2	2588	0.115	-0.0145	-
CD40	2878	0.105	-0.0204	-
CHST2	3091	0.099	-0.0238	-
CDKN1A	3181	0.097	-0.0231	-
ITGB3	3249	0.095	-0.0217	-
TNFSF9	3583	0.086	-0.0299	-
LCK	3640	0.085	-0.0285	-
PTGIR	3648	0.085	-0.0254	-
SLC31A1	3764	0.082	-0.0261	-
ATP2A2	3840	0.08	-0.0256	-
CXCL9	4005	0.076	-0.0283	-
P2RY2	4172	0.072	-0.0312	-
VIP	4329	0.068	-0.0339	-
CMKLR1	4421	0.066	-0.0345	-
FZD5	4693	0.061	-0.0415	-
CCR7	4719	0.061	-0.04	-
CSF1	5147	0.053	-0.0527	-
SLC4A4	5266	0.051	-0.0548	-
APLNR	5383	0.05	-0.0568	-
CCL24	5719	0.044	-0.0667	-
GABBR1	5836	0.042	-0.0691	-
HAS2	5843	0.041	-0.0677	-
TACR1	6272	0.035	-0.0812	-
LTA	6850	0.027	-0.1001	-
IL15RA	7146	0.023	-0.1095	-
CCRL2	7539	0.018	-0.1224	-

SEMA4D	7644	0.017	-0.1253	-
BDKRB1	7788	0.015	-0.1297	-
PVR	7955	0.013	-0.135	-
CCL7	8306	0.009	-0.1467	-
NLRP3	8307	0.009	-0.1464	-
IL1A	8931	0.004	-0.1678	-
RELA	9352	0.001	-0.1823	-
TACR3	9399	0.001	-0.1838	-
GP1BA	12540	-0.001	-0.2927	-
P2RX7	13024	-0.003	-0.3093	-
IL7R	13192	-0.005	-0.3149	-
NMUR1	13586	-0.008	-0.3282	-
ATP2C1	14192	-0.012	-0.3487	-
SCN1B	14209	-0.013	-0.3488	-
IL15	14499	-0.015	-0.3582	-
IRAK2	14538	-0.016	-0.3589	-
CCL17	14551	-0.016	-0.3587	-
MYC	14720	-0.018	-0.3638	-
RASGRP1	14820	-0.019	-0.3665	-
C3AR1	14974	-0.02	-0.371	-
IL10RA	15046	-0.021	-0.3727	-
ADGRE1	15052	-0.021	-0.372	-
PTGER4	15104	-0.021	-0.373	-
SLAMF1	15813	-0.029	-0.3963	-
AXL	16057	-0.032	-0.4035	-
EDN1	16121	-0.032	-0.4044	-
CLEC5A	16684	-0.038	-0.4224	-
ITGA5	16973	-0.04	-0.4308	-
CXCR6	17340	-0.044	-0.4418	-
LDLR	18115	-0.053	-0.4665	-
P2RX4	18185	-0.054	-0.4668	-
AHR	18419	-0.056	-0.4727	-
CALCRL	18574	-0.057	-0.4758	-
CD70	18579	-0.058	-0.4737	-
CSF3	18614	-0.058	-0.4726	-
NFKB1	18749	-0.059	-0.4749	-
CCL2	18849	-0.06	-0.4759	-
GCH1	19045	-0.062	-0.4802	-
STAB1	19850	-0.071	-0.5053	-
SGMS2	20101	-0.073	-0.5111	-
ACVR2A	20205	-0.074	-0.5118	-

RIPK2	20527	-0.078	-0.5198	-
KCNA3	20582	-0.079	-0.5186	-
OLR1	20658	-0.079	-0.5181	-
PIK3R5	20810	-0.081	-0.5202	-
CYBB	20863	-0.082	-0.5187	-
F3	21251	-0.087	-0.5288	-
MSR1	21611	-0.091	-0.5376	↑ GDM
ACVR1B	21634	-0.091	-0.5348	↑ GDM
HBEGF	21705	-0.093	-0.5336	↑ GDM
NPFFR2	21887	-0.095	-0.5361	↑ GDM
INHBA	21906	-0.095	-0.533	↑ GDM
ICAM4	22081	-0.098	-0.5352	↑ GDM
CCL20	22160	-0.099	-0.534	↑ GDM
RGS1	22208	-0.1	-0.5317	↑ GDM
LAMP3	22243	-0.1	-0.529	↑ GDM
MEFV	22297	-0.101	-0.5268	↑ GDM
PSEN1	22311	-0.101	-0.5233	↑ GDM
EIF2AK2	22345	-0.101	-0.5205	↑ GDM
EREG	22452	-0.103	-0.5201	↑ GDM
ABCA1	22656	-0.106	-0.523	↑ GDM
IL1R1	22683	-0.106	-0.5197	↑ GDM
MARCO	22687	-0.106	-0.5157	↑ GDM
RAF1	22737	-0.107	-0.5132	↑ GDM
KIF1B	22746	-0.107	-0.5092	↑ GDM
HRH1	22749	-0.107	-0.5051	↑ GDM
SELENOS	22831	-0.108	-0.5037	↑ GDM
BTG2	22870	-0.109	-0.5007	↑ GDM
PTAFR	22914	-0.109	-0.4979	↑ GDM
TNFRSF1B	22930	-0.11	-0.4941	↑ GDM
GPR183	23107	-0.112	-0.4958	↑ GDM
TAPBP	23247	-0.114	-0.4962	↑ GDM
MEP1A	23320	-0.115	-0.4941	↑ GDM
SLC31A2	23369	-0.116	-0.4912	↑ GDM
TNFRSF9	23520	-0.119	-0.4918	↑ GDM
ATP2B1	23627	-0.12	-0.4907	↑ GDM
ADORA2B	23715	-0.121	-0.4889	↑ GDM
TNFSF15	23927	-0.125	-0.4914	↑ GDM
GNA15	23938	-0.125	-0.4868	↑ GDM
SRI	23961	-0.125	-0.4827	↑ GDM
CXCL11	23970	-0.125	-0.478	↑ GDM
NOD2	24170	-0.128	-0.4799	↑ GDM

LCP2	24245	-0.13	-0.4773	↑ GDM
PTGER2	24337	-0.131	-0.4753	↑ GDM
IL18	24697	-0.138	-0.4823	↑ GDM
HIF1A	24748	-0.139	-0.4786	↑ GDM
IL18R1	24824	-0.14	-0.4757	↑ GDM
GNAI3	24902	-0.142	-0.4728	↑ GDM
ABI1	24917	-0.142	-0.4677	↑ GDM
IL4R	25014	-0.144	-0.4654	↑ GDM
SELL	25043	-0.145	-0.4607	↑ GDM
CXCL6	25067	-0.145	-0.4558	↑ GDM
PTPRE	25082	-0.145	-0.4505	↑ GDM
RTP4	25098	-0.146	-0.4453	↑ GDM
LY6E	25115	-0.146	-0.4401	↑ GDM
OSMR	25125	-0.146	-0.4347	↑ GDM
IFNGR2	25209	-0.148	-0.4318	↑ GDM
DCBLD2	25260	-0.149	-0.4277	↑ GDM
TLR2	25273	-0.149	-0.4222	↑ GDM
IFNAR1	25343	-0.15	-0.4187	↑ GDM
ITGB8	25416	-0.152	-0.4152	↑ GDM
IL10	25466	-0.153	-0.4109	↑ GDM
LYN	25533	-0.155	-0.4071	↑ GDM
PCDH7	25585	-0.156	-0.4027	↑ GDM
MXD1	25624	-0.157	-0.3979	↑ GDM
CX3CL1	25634	-0.157	-0.392	↑ GDM
KLF6	25720	-0.159	-0.3887	↑ GDM
MMP14	25754	-0.16	-0.3836	↑ GDM
MET	25941	-0.165	-0.3836	↑ GDM
PLAUR	25975	-0.165	-0.3782	↑ GDM
PDE4B	25983	-0.166	-0.3719	↑ GDM
SPHK1	26006	-0.166	-0.3661	↑ GDM
HPN	26015	-0.167	-0.3599	↑ GDM
EMP3	26047	-0.167	-0.3544	↑ GDM
KCNJ2	26059	-0.167	-0.3482	↑ GDM
CD48	26165	-0.17	-0.3452	↑ GDM
TIMP1	26400	-0.176	-0.3464	↑ GDM
EBI3	26417	-0.176	-0.34	↑ GDM
PDPN	26441	-0.177	-0.3338	↑ GDM
RNF144B	26455	-0.177	-0.3273	↑ GDM
LIF	26492	-0.178	-0.3216	↑ GDM
IRF1	26578	-0.181	-0.3174	↑ GDM
CSF3R	26612	-0.181	-0.3114	↑ GDM

TLR1	26623	-0.182	-0.3046	↑ GDM
OPRK1	26812	-0.187	-0.3038	↑ GDM
RHOG	26877	-0.189	-0.2985	↑ GDM
SLC7A2	26928	-0.191	-0.2928	↑ GDM
SELE	26957	-0.192	-0.2862	↑ GDM
C5AR1	26965	-0.192	-0.2789	↑ GDM
GPR132	27040	-0.195	-0.2738	↑ GDM
IL18RAP	27139	-0.198	-0.2694	↑ GDM
ICAM1	27170	-0.199	-0.2626	↑ GDM
CD69	27281	-0.204	-0.2584	↑ GDM
CD82	27435	-0.21	-0.2554	↑ GDM
GPC3	27489	-0.212	-0.2489	↑ GDM
IL1B	27525	-0.213	-0.2418	↑ GDM
NMI	27527	-0.213	-0.2334	↑ GDM
BST2	27554	-0.214	-0.2259	↑ GDM
CD14	27615	-0.217	-0.2195	↑ GDM
IFITM1	27706	-0.22	-0.214	↑ GDM
CD55	27900	-0.229	-0.2117	↑ GDM
OSM	27936	-0.231	-0.2038	↑ GDM
FFAR2	28073	-0.239	-0.1991	↑ GDM
NFKBIA	28087	-0.239	-0.1902	↑ GDM
ADRM1	28101	-0.24	-0.1812	↑ GDM
TNFSF10	28132	-0.242	-0.1727	↑ GDM
FPR1	28185	-0.245	-0.1649	↑ GDM
CCL5	28212	-0.247	-0.1561	↑ GDM
IL12B	28333	-0.256	-0.1502	↑ GDM
AQP9	28338	-0.256	-0.1403	↑ GDM
TLR3	28393	-0.26	-0.1319	↑ GDM
IRF7	28398	-0.261	-0.1218	↑ GDM
PROK2	28414	-0.262	-0.112	↑ GDM
NAMPT	28449	-0.266	-0.1027	↑ GDM
SERPINE1	28460	-0.267	-0.0926	↑ GDM
BEST1	28544	-0.276	-0.0846	↑ GDM
SCARF1	28660	-0.29	-0.0772	↑ GDM
ADM	28705	-0.295	-0.0671	↑ GDM
TNFAIP6	28723	-0.299	-0.056	↑ GDM
TPBG	28824	-0.319	-0.0469	↑ GDM
CXCL10	28833	-0.322	-0.0345	↑ GDM
CXCL8	28849	-0.325	-0.0223	↑ GDM
IL6	28962	-0.366	-0.0118	↑ GDM
ROS1	28971	-0.372	0.0026	↑ GDM

Table S20. Gene set enrichment analysis details for HALLMARK_TNFA_SIGNALING_VIA_NFKB gene set comparing healthy Ctrl and GDM cord blood mononuclear cells (CBMCs).

Gene symbol	Rank in gene list	Rank metric score	Running enrichment score	Enrichment
SPSB1	144	0.334	0.0074	-
ICOSLG	404	0.264	0.0082	-
EGR3	1017	0.191	-0.006	-
NR4A1	1463	0.162	-0.0155	-
EGR1	2434	0.121	-0.0446	-
SMAD3	2462	0.12	-0.0411	-
FJX1	2668	0.113	-0.0441	-
TUBB2A	2730	0.111	-0.0421	-
CD83	3015	0.102	-0.0482	-
CDKN1A	3181	0.097	-0.0503	-
PER1	3292	0.093	-0.0507	-
TNFSF9	3583	0.086	-0.0576	-
PMEPA1	4208	0.071	-0.0766	-
NR4A3	4346	0.068	-0.0788	-
TRIP10	4582	0.063	-0.0846	-
CSF1	5147	0.053	-0.1022	-
ACKR3	5240	0.052	-0.1035	-
BTG3	5344	0.05	-0.1052	-
SIK1	5518	0.047	-0.1095	-
MAP2K3	5571	0.046	-0.1096	-
TSC22D1	5633	0.045	-0.11	-
DNAJB4	6080	0.038	-0.1241	-
FOSL1	6335	0.034	-0.1316	-
FOSB	6604	0.03	-0.1398	-
LAMB3	6685	0.029	-0.1415	-
IL15RA	7146	0.023	-0.1566	-
CCRL2	7539	0.018	-0.1696	-
CSF2	8173	0.011	-0.1911	-
IL1A	8931	0.004	-0.2172	-
BIRC2	9206	0.002	-0.2266	-
RELA	9352	0.001	-0.2316	-
TRAF1	13023	-0.003	-0.3587	-
IL7R	13192	-0.005	-0.3644	-
ZBTB10	14475	-0.015	-0.4083	-
YRDC	14661	-0.017	-0.4141	-
FUT4	14671	-0.017	-0.4137	-
MYC	14720	-0.018	-0.4147	-
PTGER4	15104	-0.021	-0.4272	-

CD44	15119	-0.022	-0.4269	-
EFNA1	15432	-0.025	-0.4368	-
HES1	15679	-0.028	-0.4443	-
EDN1	16121	-0.032	-0.4584	-
B4GALT1	16328	-0.034	-0.4643	-
TANK	16446	-0.036	-0.467	-
TNFAIP8	17069	-0.041	-0.4871	-
REL	17071	-0.041	-0.4856	-
FOS	17214	-0.043	-0.4889	-
SQSTM1	17310	-0.044	-0.4906	-
CLCF1	17376	-0.045	-0.4912	-
EGR2	17414	-0.045	-0.4908	-
NFAT5	17750	-0.049	-0.5006	-
BIRC3	17944	-0.051	-0.5054	-
PANX1	18025	-0.052	-0.5063	-
LDLR	18115	-0.053	-0.5074	-
FOSL2	18412	-0.056	-0.5156	-
PTX3	18556	-0.057	-0.5185	-
NFKB1	18749	-0.059	-0.5229	-
CCL2	18849	-0.06	-0.5241	-
SERPINB8	18896	-0.061	-0.5235	-
KYNU	18928	-0.061	-0.5223	-
GCH1	19045	-0.062	-0.524	-
CCND1	19105	-0.063	-0.5237	-
STAT5A	19325	-0.065	-0.5289	-
SNN	19453	-0.067	-0.5309	-
ID2	19580	-0.068	-0.5327	-
ZC3H12A	20202	-0.074	-0.5515	-
RIPK2	20527	-0.078	-0.5599	-
OLR1	20658	-0.079	-0.5614	-
IL6ST	20726	-0.08	-0.5608	-
DENND5A	20755	-0.08	-0.5588	-
MAP3K8	20959	-0.083	-0.5628	↑ GDM
IER5	20999	-0.083	-0.561	↑ GDM
IL23A	21078	-0.084	-0.5606	↑ GDM
RCAN1	21154	-0.085	-0.5601	↑ GDM
F3	21251	-0.087	-0.5602	↑ GDM
GADD45A	21348	-0.088	-0.5603	↑ GDM
G0S2	21424	-0.089	-0.5596	↑ GDM
B4GALT5	21472	-0.089	-0.5579	↑ GDM
EIF1	21575	-0.091	-0.5581	↑ GDM

BHLHE40	21665	-0.092	-0.5578	↑ GDM
HBEGF	21705	-0.093	-0.5557	↑ GDM
NFKBIE	21847	-0.094	-0.5571	↑ GDM
CXCL3	21862	-0.095	-0.5541	↑ GDM
INHBA	21906	-0.095	-0.5521	↑ GDM
JAG1	22130	-0.098	-0.5562	↑ GDM
IRS2	22136	-0.099	-0.5527	↑ GDM
CCL20	22160	-0.099	-0.5499	↑ GDM
TIPARP	22431	-0.102	-0.5554	↑ GDM
DUSP2	22540	-0.104	-0.5553	↑ GDM
TGIF1	22601	-0.105	-0.5535	↑ GDM
KDM6B	22610	-0.105	-0.5499	↑ GDM
ABCA1	22656	-0.106	-0.5476	↑ GDM
BTG2	22870	-0.109	-0.551	↑ GDM
KLF9	23023	-0.111	-0.5521	↑ GDM
GPR183	23107	-0.112	-0.5509	↑ GDM
DRAM1	23184	-0.113	-0.5493	↑ GDM
CD80	23188	-0.113	-0.5452	↑ GDM
TNC	23229	-0.114	-0.5424	↑ GDM
KLF4	23289	-0.115	-0.5402	↑ GDM
PDLIM5	23349	-0.116	-0.538	↑ GDM
TNFRSF9	23520	-0.119	-0.5395	↑ GDM
ATP2B1	23627	-0.12	-0.5387	↑ GDM
BTG1	23735	-0.122	-0.5379	↑ GDM
CXCL11	23970	-0.125	-0.5414	↑ GDM
MCL1	23988	-0.126	-0.5374	↑ GDM
AREG	24195	-0.129	-0.5398	↑ GDM
RIGI	24258	-0.13	-0.5371	↑ GDM
TNIP1	24304	-0.131	-0.5338	↑ GDM
PNRC1	24338	-0.131	-0.5301	↑ GDM
JUN	24393	-0.132	-0.5271	↑ GDM
NR4A2	24441	-0.133	-0.5238	↑ GDM
SGK1	24478	-0.134	-0.5201	↑ GDM
IL18	24697	-0.138	-0.5226	↑ GDM
CFLAR	24805	-0.14	-0.5211	↑ GDM
RNF19B	24819	-0.14	-0.5164	↑ GDM
TNFAIP3	24853	-0.141	-0.5124	↑ GDM
PLPP3	25035	-0.144	-0.5133	↑ GDM
CXCL6	25067	-0.145	-0.509	↑ GDM
TNFAIP2	25073	-0.145	-0.5038	↑ GDM
PTPRE	25082	-0.145	-0.4987	↑ GDM

ETS2	25159	-0.147	-0.4959	↑ GDM
EHD1	25194	-0.148	-0.4917	↑ GDM
IFNGR2	25209	-0.148	-0.4867	↑ GDM
KLF10	25231	-0.148	-0.4819	↑ GDM
PLAU	25254	-0.148	-0.4772	↑ GDM
TLR2	25273	-0.149	-0.4723	↑ GDM
BCL6	25445	-0.153	-0.4726	↑ GDM
RHOB	25461	-0.153	-0.4675	↑ GDM
PTGS2	25477	-0.154	-0.4623	↑ GDM
PLK2	25481	-0.154	-0.4567	↑ GDM
NFKB2	25519	-0.155	-0.4523	↑ GDM
PPP1R15A	25522	-0.155	-0.4467	↑ GDM
CXCL2	25554	-0.155	-0.442	↑ GDM
F2RL1	25566	-0.156	-0.4366	↑ GDM
BMP2	25597	-0.156	-0.4319	↑ GDM
MXD1	25624	-0.157	-0.427	↑ GDM
KLF6	25720	-0.159	-0.4244	↑ GDM
PLAUR	25975	-0.165	-0.4271	↑ GDM
PDE4B	25983	-0.166	-0.4212	↑ GDM
CCNL1	25988	-0.166	-0.4152	↑ GDM
SPHK1	26006	-0.166	-0.4097	↑ GDM
SOD2	26103	-0.169	-0.4068	↑ GDM
VEGFA	26118	-0.169	-0.401	↑ GDM
IFIH1	26394	-0.176	-0.4041	↑ GDM
DUSP5	26462	-0.177	-0.3998	↑ GDM
SLC16A6	26464	-0.178	-0.3933	↑ GDM
LIF	26492	-0.178	-0.3877	↑ GDM
TRIB1	26524	-0.179	-0.3821	↑ GDM
DUSP4	26535	-0.18	-0.3758	↑ GDM
GEM	26554	-0.18	-0.3698	↑ GDM
NFE2L2	26573	-0.181	-0.3638	↑ GDM
IRF1	26578	-0.181	-0.3572	↑ GDM
PLEK	26774	-0.186	-0.3571	↑ GDM
KLF2	26827	-0.188	-0.352	↑ GDM
RELB	26860	-0.189	-0.3461	↑ GDM
TNIP2	26919	-0.191	-0.341	↑ GDM
CCN1	26930	-0.191	-0.3343	↑ GDM
PFKFB3	26932	-0.191	-0.3273	↑ GDM
CCL4	26952	-0.192	-0.3208	↑ GDM
MARCKS	26962	-0.192	-0.3141	↑ GDM
ZFP36	27019	-0.194	-0.3088	↑ GDM

ICAM1	27170	-0.199	-0.3067	↑ GDM
TAP1	27240	-0.203	-0.3016	↑ GDM
CD69	27281	-0.204	-0.2954	↑ GDM
SLC2A6	27358	-0.207	-0.2904	↑ GDM
IER2	27382	-0.208	-0.2835	↑ GDM
SLC2A3	27415	-0.209	-0.2769	↑ GDM
IL1B	27525	-0.213	-0.2728	↑ GDM
LITAF	27673	-0.218	-0.2698	↑ GDM
NINJ1	27736	-0.221	-0.2638	↑ GDM
BCL2A1	27966	-0.233	-0.2632	↑ GDM
DUSP1	28058	-0.238	-0.2575	↑ GDM
NFIL3	28077	-0.239	-0.2493	↑ GDM
SERPINB2	28083	-0.239	-0.2407	↑ GDM
NFKBIA	28087	-0.239	-0.2319	↑ GDM
CCL5	28212	-0.247	-0.2271	↑ GDM
GADD45B	28247	-0.25	-0.2191	↑ GDM
SOCS3	28252	-0.25	-0.21	↑ GDM
SAT1	28267	-0.251	-0.2012	↑ GDM
IL12B	28333	-0.256	-0.194	↑ GDM
BCL3	28336	-0.256	-0.1846	↑ GDM
MAFF	28365	-0.258	-0.176	↑ GDM
SDC4	28432	-0.265	-0.1685	↑ GDM
NAMPT	28449	-0.266	-0.1592	↑ GDM
ATF3	28452	-0.267	-0.1494	↑ GDM
IFIT2	28458	-0.267	-0.1398	↑ GDM
SERPINE1	28460	-0.267	-0.1299	↑ GDM
CEBPB	28488	-0.269	-0.1209	↑ GDM
JUNB	28490	-0.27	-0.111	↑ GDM
IER3	28675	-0.292	-0.1066	↑ GDM
PHLDA2	28692	-0.294	-0.0963	↑ GDM
CEBPD	28706	-0.295	-0.0858	↑ GDM
TNFAIP6	28723	-0.299	-0.0753	↑ GDM
TNF	28766	-0.306	-0.0655	↑ GDM
GFPT2	28790	-0.312	-0.0548	↑ GDM
CXCL10	28833	-0.322	-0.0443	↑ GDM
CXCL1	28880	-0.332	-0.0337	↑ GDM
MSC	28894	-0.336	-0.0217	↑ GDM
PHLDA1	28948	-0.36	-0.0102	↑ GDM
IL6	28962	-0.366	0.0029	↑ GDM

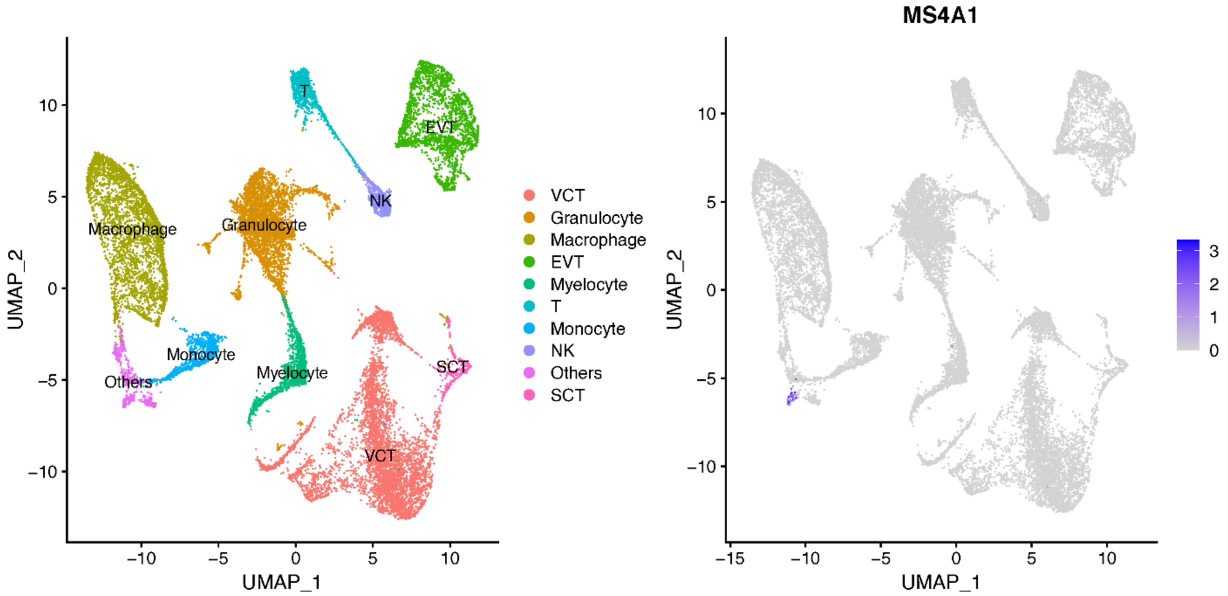

Figure S1. scRNA-seq of placenta from healthy and gestational diabetes mellitus (GDM)-impacted pregnancy. Uniform manifold approximation and projection (UMAP) plot visualizing the cellular compositions of healthy and GDM placenta analyzed by scRNA-seq (left) and feature plot for the expression of B cell marker *MS4A1* (right).

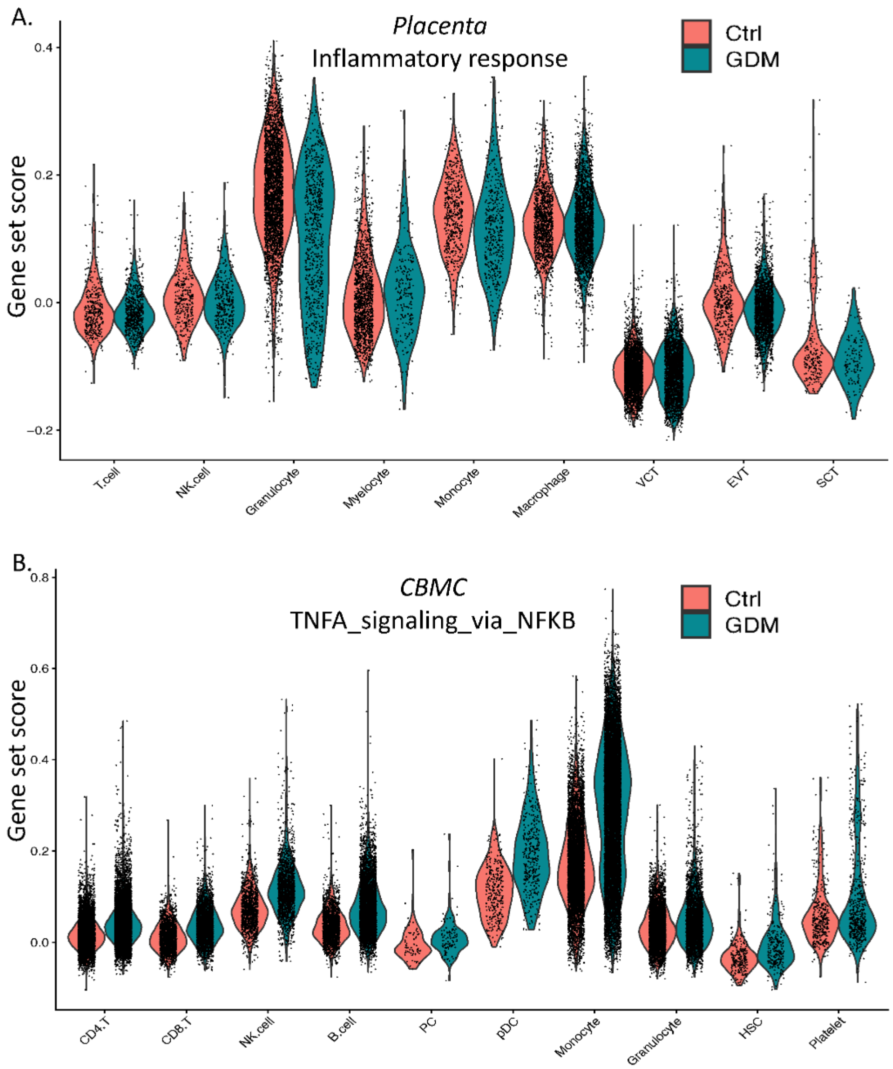

Figure S2. Gene set score analyses for healthy and gestational diabetes mellitus (GDM)-impacted placenta and cord blood mononuclear cell (CBMC). A. Violin plots for the gene set scores for inflammatory response gene set among different cell types in placenta from healthy control (red) and GDM-affected (dark green) pregnancies. **B.** Violin plots for the gene set scores for TNFA_signaling_via_NFKB gene set among different cell types in CBMCs from healthy control (red) and GDM-affected (dark green) pregnancies.

References

[1] Love MI, Huber W, Anders S (2014) Moderated estimation of fold change and dispersion for RNA-seq data with DESeq2. Genome Biol 15(12): 550. 10.1186/s13059-014-0550-8

[2] Hao Y, Hao S, Andersen-Nissen E, et al. (2021) Integrated analysis of multimodal single-cell data. Cell 184(13): 3573-3587 e3529. 10.1016/j.cell.2021.04.048

[3] Welch JD, Kozareva V, Ferreira A, Vanderburg C, Martin C, Macosko EZ (2019) Single-Cell Multi-omic Integration Compares and Contrasts Features of Brain Cell Identity. Cell 177(7): 1873-1887 e1817. 10.1016/j.cell.2019.05.006

[4] Subramanian A, Tamayo P, Mootha VK, et al. (2005) Gene set enrichment analysis: a knowledge-based approach for interpreting genome-wide expression profiles. Proc Natl Acad Sci U S A 102(43): 15545-15550. 10.1073/pnas.0506580102

[5] Yang Y, Guo F, Peng Y, et al. (2021) Transcriptomic Profiling of Human Placenta in Gestational Diabetes Mellitus at the Single-Cell Level. Front Endocrinol (Lausanne) 12: 679582. 10.3389/fendo.2021.679582

[6] Yin M, Zhang Y, Li X, et al. (2024) Adverse effects of gestational diabetes mellitus on fetal monocytes revealed by single-cell RNA sequencing. iScience 27(1): 108637. 10.1016/j.isci.2023.108637

[7] Salcedo-Tacuma D, Bonilla L, Montes MCG, et al. (2022) Transcriptome dataset of omental and subcutaneous adipose tissues from gestational diabetes patients. Sci Data 9(1): 344. 10.1038/s41597-022-01457-5

[8] Stirm L, Huypens P, Sass S, et al. (2018) Maternal whole blood cell miRNA-340 is elevated in gestational diabetes and inversely regulated by glucose and insulin. Sci Rep 8(1): 1366. 10.1038/s41598-018-19200-9

[9] Lu S, Wang J, Kakongoma N, et al. (2022) DNA methylation and expression profiles of placenta and umbilical cord blood reveal the characteristics of gestational diabetes mellitus patients and offspring. Clin Epigenetics 14(1): 69. 10.1186/s13148-022-01289-5

[10] Ambra R, Manca S, Palumbo MC, et al. (2014) Transcriptome analysis of human primary endothelial cells (HUVEC) from umbilical cords of gestational diabetic mothers reveals candidate sites for an epigenetic modulation of specific gene expression. Genomics 103(5-6): 337-348. 10.1016/j.ygeno.2014.03.003

[11] Pinney SE, Joshi A, Yin V, et al. (2020) Exposure to Gestational Diabetes Enriches Immune-Related Pathways in the Transcriptome and Methylome of Human Amniocytes. J Clin Endocrinol Metab 105(10): 3250-3264. 10.1210/clinem/dgaa466
